# Elimination of myotonia improves myopathy in a muscleblind-like knockout model of myotonic dystrophy

**DOI:** 10.1038/s41467-026-75243-x

**Published:** 2026-07-08

**Authors:** Matthew T. Sipple, Sakura A. Hamazaki, Vanessa Todorow, Lily A. Cisco, Katherine M. Lupia, Christina S. Heil, Peter Meinke, Charles A. Thornton, John D. Lueck

**Affiliations:** 1 Medical Scientist Training Program, University of Rochester School of Medicine and Dentistry, Rochester, NY USA; 2 Department of Pharmacology and Physiology, University of Rochester School of Medicine and Dentistry, Rochester, NY USA; 3 Department of Biology, University of Rochester, Rochester, NY USA; 4 Yale University, New Haven, CT USA; 5 Department of Neurology, University of Rochester School of Medicine and Dentistry, Rochester, NY USA; 6 Department of Neurology, Friedrich-Baur-Institute, LMU Clinics, Munich, Germany; 7 Center for RNA Biology, University of Rochester School of Medicine and Dentistry, Rochester, NY USA

**Keywords:** Neuromuscular disease, Physiology, Transcriptomics

## Abstract

A cardinal sign of myotonic dystrophy type 1 (DM1) is myotonia, slow muscle relaxation after voluntary contraction. Myotonia results from mis-regulated splicing of chloride channel 1 (ClC-1), leading to loss of channel function and runs of involuntary action potentials in muscle fibers. Preceding the onset of weakness, myotonia is often the first symptom of DM1, and thus this raises the possibility that muscle hyperexcitability contributes to the subsequent weakness and myopathy. Here, we show that genomic deletion of ClC-1 exon 7a (E7a), a cryptic exon abnormally regulated in DM1, completely rescues of ClC-1 function and yields permanent elimination of myotonia in the muscleblind-like 1 (Mbnl1) knockout mouse model of DM1. The restoration of normal excitability results in normalization of muscle force generation, correction of fiber-type distribution, and improvement of muscle histology. E7a deletion also partially corrects the muscle transcriptome, including changes of differential gene expression and alternative splicing. These results indicate that E7a inclusion is a lynchpin splice event that contributes to myotonic myopathy, and support myotonia reduction as a therapeutic objective in DM1.

## Abstract (web summary)

Myotonia is a hallmark symptom of myotonic dystrophy (DM1). Eliminating myotonia in a DM1 mouse model improved muscle function and corrected transcriptome dysregulation, supporting myotonia as a driver of myopathy and a potential therapeutic target.

## Introduction

Myotonic dystrophy type 1 (DM1) is an autosomal dominant degenerative disorder affecting nearly every organ system, with prominent muscle involvement that accounts for the majority of mortality^1^. The genetic cause is a trinucleotide repeat (CTG)_n_ expansion (*n* > 50) in the DM1 protein kinase (DMPK) gene that when transcribed, exerts a toxic gain-of-function. DMPK transcripts carrying expanded (CUG)_n_ repeats (CUG^exp^) form nuclear foci that sequester RNA-binding proteins in the muscleblind-like (MBNL) family^2^. Depletion of these crucial proteins disrupts developmentally regulated alternative splicing and polyadenylation for a subset of transcripts and results in cellular dysfunction that manifests as myopathy in skeletal muscle. Direct knockout of Mbnl1 in mice (Mbnl1^−/−^) recapitulates much of the dysregulation in splicing seen in DM1 and results in skeletal muscle phenotypes, including myotonia and weakness, and is generally regarded as a model for mild to moderate DM1^3–5^.

DM1 was initially recognized and named for its most evident and distinctive features: myotonia, slow relaxation after muscle contraction, and muscle degeneration which preferentially affects oropharyngeal, hand, and forearm muscles^6^. Myotonia is generally the first symptom and is used in the clinic to distinguish DM1 from other forms of muscular dystrophy^7^. Recently, studies have also shown that myotonia is a DM1 feature that responds rapidly to molecularly targeted therapy^8^. Consequently, it is used in clinical studies as a physiological indicator of whether RNA toxicity is reduced.

Ion channels and transporters that participate in muscle excitation-contraction coupling (ECC) are particularly affected by splicing dysregulation in DM1^9–12^. These include, *CLCN*1 that encodes the main voltage-sensitive chloride channel (ClC-1) in skeletal muscle. Several *CLCN*1 splice events are altered in DM1 mouse models and patients, including abnormal inclusion of cryptic exons, intron retention, or a combination of several of these events. All produce a frameshift in Clcn1 mRNA that results in truncation of the ClC-1 protein, loss of Cl- conductance, and myotonia^13–16^. In contrast, mis-splicing of *CACNA*1*S*, encoding the Ca_V_1.1 calcium channel required for ECC, is more representative of splicing defects affecting other transcripts, a single cassette exon (exon 29), which when skipped leads to loss of an extracellular domain in the channel protein and increased calcium entry during muscle activity^9,17–19^. The transcriptomic changes stemming from MBNL sequestration are extensive, creating an ongoing challenge to determine which transcripts are direct targets of MBNL and which downstream effects are primarily responsible for muscle dysfunction and degeneration, the most disabling aspect of DM1. For example, loss of ClC-1 is known to cause myotonia and forced exclusion of exon 29 in Ca_V_1.1 causes late-onset myopathy in mice, but neither condition in isolation resembles the progressive myopathy observed in DM1^16,18^.

Recently, we used exon deletion to force several ECC-related splicing defects in mice, and through combinatorial breeding showed that loss of ClC-1 combined with Ca_V_1.1 E29 inclusion was highly deleterious, resulting in aggravated muscle excitability, loss of muscle force generation, and premature death. Interestingly, the mice with chloride/calcium bi-channelopathy showed marked improvement of survival when treated with calcium channel blockers^9^. These results indicated that myotonic runs of action potentials are highly deleterious when combined with Ca_V_1.1 E29 inclusion, likely due to calcium overload, and raised the possibility that myotonia may interact adversely with other splicing defects, perhaps as a function of mechanical stress, metabolic demand, transcriptome remodeling, or other factors. Furthermore, this concept fits with the clinical observation that muscles selectively affected by DM1 exhibit myotonia initially and subsequent weakness and wasting^20^.

Accordingly, the status of myotonia as a driver of myopathy in DM1 and the value of myotonia suppression as a disease-modifying strategy requires further investigation. Therefore, to further evaluate the role of myotonia in DM1, we sought here to remove myotonia permanently and specifically in a DM1 mouse model that otherwise exhibits transcriptome changes and clinical features of DM1. To this end we deleted exon 7a (E7a) of *Clcn*1 (ClC-1^∆E7a^) in Mbnl1^−/−^ mice. Notably, we found that genomic deletion of this single exon restored splicing of all exons in the *Clcn*1 transcript to wild-type patterns, probably due to correction of the reading frame and restoration of nonsense-mediated decay surveillance. The sustained elimination of *Clcn*1 E7a caused complete rescue of chloride conductance and myotonia, leading to muscular function closer to WT mice with significant reduction in aberrant global transcript expression and splicing and DM1 muscle histopathology. Accordingly, our results provide further support for the concept that myotonia, through inclusion of *CLCN*1 E7a, is a driver of DM1 myopathy progression.

## Results

### Generation of a Clcn1 exon 7a deletion mouse model to assess the impact of forced exon exclusion

**Fig. 1:**
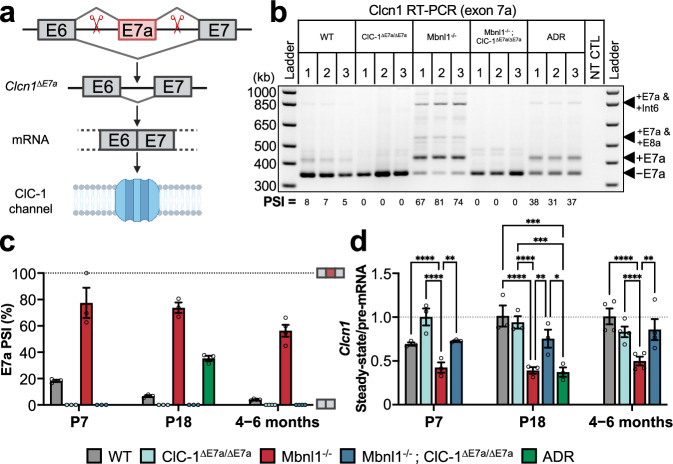
Generating a forced exon 7a exclusion mouse model that corrects changes in *Clcn*1 splicing and RNA stability in Mbnl1^−/−^ mice. **a** Schematic of CRISPR/Cas9 approach to develop a myotonia-resistant mouse model by excision of the exon 7a sequence from the *Clcn*1 gene. Scissors represent approximate location of the gRNA sequences (sequences provided in Supplementary Fig. 1). Created in BioRender. Sipple, M. (2026) https://BioRender.com/t30q877. **b** RT-PCR products generated with primers flanking exon 7a from RNA isolated from quadriceps muscle from WT, ClC-1^∆E7a/∆E7a^, Mbnl1^−/−^, Mbnl1^−/−^; ClC-1^∆E7a/∆E7a^, and ADR (NMD control) mice (P18). Each lane represents an individual mouse, with biological replicates denoted by number. Ladder (1 kb Plus ladder, Invitrogen) and no template control (NT CTL) shown. Transcript isoforms indicated on the right with –E7a representing the adult, WT isoform. **c** Quantitation of exon 7a percent spliced in (PSI) based on relative densitometry of the amplicons shown in **b**, as well as for samples at P7 and 4–6-months of age, with bars shown mean ± SEM and individual biological replicates indicated by dots (*n* = 3 mice/genotype/timepoint at P7 and P18, *n* = 4 mice/genotype at 4–6-mth-postnatal, additional agarose gels shown in Supplementary Fig. 2). **d** To determine relative RNA stability, the ratio of relative *Clcn*1 steady-state mRNA to pre-mRNA was determined with the use of RT-qPCR (*n* = 3 mice/genotype/timepoint at P7 and P18, *n* = 4 mice/genotype at 4–6-mth-postnatal; completed in technical triplicate). Dots represent biological replicates and bars plotted mean ± SEM. Statistical comparisons were completed with two-way ANOVAs with two-sided Šídák’s multiple comparisons test (**c**) and Tukey’s multiple comparisons (**d**) with only significant intra-timepoint comparisons shown. Additional statistical details provided in Source Data file. Significant comparisons indicated as adjusted *p*-value: ^*^p < 0.05, ^**^p < 0.01, ^***^p < 0.001, and ^****^p < 0.0001.

ClC-1 (encoded by *CLCN*1) is the main chloride channel in skeletal muscle that plays a critical role in stabilizing the membrane potential during muscle activity. Loss-of-function mutations in *CLCN*1 cause generalized myotonia in many mammalian species, including humans with myotonia congenita and mice with arrested development of righting response (ADR, slow recovery of upright position after being placed supine). In DM1, however, the myotonia is associated with misregulated alternative splicing of *Clcn*1, and splice-shifting ASOs that blocked inclusion of a single exon, E7a, were sufficient to transiently restore Cl- currents and eliminate myotonia in mice. Accordingly, to study long-term impacts of myotonia on skeletal muscle in a mouse model with DM1, we used CRISPR/Cas9 to genomically excise E7a and proximal flanking introns, including the cognate element for Mbnl1 binding and targeting site for the splice-shifting ASOs (Fig. 1a)^21,22^. Excision of E7a was confirmed by sequencing of genomic DNA (Supplementary Fig. 1a). Then, we crossed ClC-1^∆E7a^ mice with Mbnl1 knockout (Mbnl1^−/−^) mice^3^. We chose Mbnl1^−/−^ mice for this study because they model a key mechanism of DM1, and present a consistent and well-characterized muscle phenotype, including robust myotonia, without confounding effects from differential *ACTA*1-driven transgene expression of toxic repeats^3,23^. RT-PCR analysis of Mbnl1^−^ muscle showed a marked increase of E7a inclusion (73.7 ± 4.0%) compared to WT controls (6.7 ± 0.7%, Fig. 1b, c, and Supplementary Fig. 2), as expected, whereas no products with E7a inclusion were observed in ClC-1^∆E7a/∆E7a^ and Mbnl1^−/−^; ClC-1^∆E7a/∆E7a^ mice. Importantly, splicing of flanking exons 6 and 7 was not perturbed in these exon excision models (Fig. 1b).

Mis-regulated splicing of *Clcn*1 in DM1 is associated with decreased accumulation of *Clcn*1 mRNA, potentially as a result of nonsense-mediated mRNA decay (NMD), as E7a inclusion causes a frameshift that yields a downstream PTC^13^. To assess the impact of E7a splicing on RNA stability, we examined levels of processed mRNA relative to primary Clcn1 transcripts in skeletal muscle (Supplementary Fig. 1b, c). Compared to WT controls, the ratio of Clcn1 mRNA to pre-mRNA in Mbnl1^−/−^ samples was decreased at all timepoints (*p* < 0.0001), similar to effects of a direct frame-shift mutation (ADR mice homozygous for a single nucleotide deletion in *Clcn*1 exon 18, Fig. 1d). In contrast, Clcn1 mRNA stability was restored to WT levels in Mbnl1^−/−^; ClC-1^∆E7a/∆E7a^ mice at P7, P18 and the 4–6-month timepoints (*p*-value of 0.0018, 0.022, and 0.0018, respectively, for Mbnl1^−/−^ vs. Mbnl1^−/−^; ClC-1^∆E7a/∆E7a^). Across timepoints and models without forced exclusion, *Clcn*1 stability was inversely correlated to E7a inclusion, further supporting the impact of this poison exon in controlling expression (Supplementary Fig. 2b). Moreover, these results indicate that E7a deletion is sufficient to stabilize *Clcn*1 mRNA in the absence of Mbnl1.

### Whole-transcript sequencing revealed changes in Clcn1 transcript isoform utilization in the setting of exon 7a deletion, Mbnl1 depletion, and frameshift mutation

**Fig. 2:**
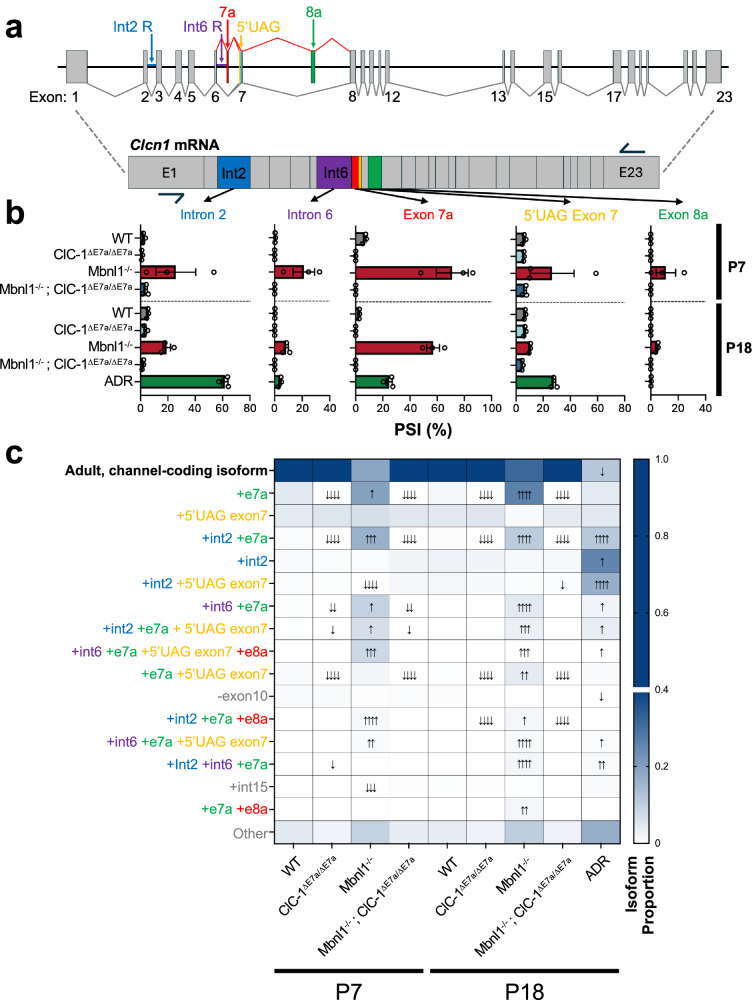
Whole-transcript sequencing illustrates Mbnl1 dependent and independent splicing events in the setting of NMD causing splicing changes. Whole-*Clcn*1 amplicon sequencing libraries were generated for both early (P7) and late (P18) postnatal mice with RNA isolated from quadriceps muscle of WT, ClC-1^∆E7a/∆E7a^, Mbnl1^−/−^, and Mbnl1^−/−^; ClC-1^∆E7a/∆E7a^ genotypes as well as late post-natal ADR samples used as the NMD control (n = 3 mice/genotype/timepoint). **a** Representation of the murine *Clcn*1 gene with constitutive exons (gray) and common alternative splice elements seen in DM1 annotated in distinct colors. Downward projection represents a schematic of post-splicing *Clcn*1 mRNA containing all the above annotated alternative splicing features. Approximate forward and reverse primer locations used for generating the whole-*Clcn*1 coding region amplicons for long-read sequencing libraries shown as arrows. **b** Quantification of prominent individual splice elements expressed as percent spliced in (PSI), based on whole-length reads. Bars shown mean ± SEM with biological replicates shown as dots. **c** Assessment of transcript isoform utilization with FLAIR. Reads quantified for the adult, protein-coding isoform, and the 15 most abundant alternate isoforms seen in the library, shown as a heat map; rows indicate transcript isoform labeled based on the elements distinct from the adult, protein-coding isoform and columns indicate the different genotype/timepoint conditions assessed. Color intensity of each box represents the average proportion of that isoform for each group (*n* = 3 mice/genotype/timepoint), as per the scale bar to right (note, the broken scale skewed to lower isoform proportions to visually highlight differences in the lower abundance alternative transcript isoforms). Arrows superimposed on boxes represent statistical comparisons between each genotype/timepoint and the age-matched wild-type samples using DESeq2 Wald test; number of arrows indicates Benjamini and Hochberg adjusted (FDR < 0.05) significance level (*p* < 0.05 ↑, *p* < 0.01 ↑↑, *p* < 0.001 ↑↑↑, or *p* < 0.0001 ↑↑↑↑) and direction indicates up/down regulated compared to age-matched wild-type samples.

RT-PCR of *Clcn*1 from exon 5 to 8 confirmed several splicing changes in Mbnl1^−/−^ samples, including inclusion of intron 6 and exon 8a, that appeared to be reduced in Mbnl1^−/−^; ClC-1^∆E7a/∆E7a^ samples (Fig. 1b). This suggested that removal of E7a impacted isoform expression beyond exclusion of a single exon, and raised the possibility that E7a deletion affected the interdependence of intron splicing in *Clcn*1 transcripts *in cis*^24–27^. To further examine these effects, we generated *Clcn*1 cDNAs extending from the first to final (23rd) exon by RT-PCR and then used long-read sequencing (PacBio) to examine all splice junctions (Supplementary Fig. 3a). Quadriceps samples were collected from early (P7) and later (P18) postnatal ages, representing developmental stages with lower or higher intrinsic Mbnl1 activity, respectively, from WT, ClC-1^∆E7a/∆E7a^, Mbnl1^−/−^, and Mbnl1^−/−^; ClC-1^∆E7a/∆E7a^ mice^28^. To analyze the full-length *Clcn*1 reads, we used an established pipeline (FLAIR) to quantify the frequency of individual splice events (Fig. 2a, b) as well as differential transcript usage (Fig. 2c)^29^. The heatmap in Fig. 2c shows the mean fractional representation of each isoform in mice with the specified genotype and age (P7 or P18, 3 mice per genotype/timepoint).

The spliceosome output in WT mice is normally surveilled by NMD, with aberrant splice isoforms likely subject to more rapid degradation than the canonical protein-coding product. Under this condition, the steady-state frequency of E7a inclusion, intron 2 retention, and other splice variants was low at P7 and P18, and the canonical splice product encoding functional ClC-1 channel was highly predominant (top row in Fig. 2c).

In Mbnl1^−/−^ mice, the *Clcn*1 transcript repertoire was markedly different. The frequency of E7a inclusion and intron 2 retention was much higher than WT, and values obtained by long-read sequencing were highly correlated with short-amplicon RT-PCR (Supplementary Fig. 3b, c). More specifically, Mbnl1^−/−^ mice showed predominant expression of isoforms that include E7a at both P7 and P18, including some that exclusively feature E7a inclusion as well as others that couple E7a inclusion with intron 2 retention, intron 6 retention, and/or exon 8a inclusion. However, isoforms with isolated intron 2 retention were rarely observed, and were not upregulated compared to WT controls (Fig. 2c). Thus, a key finding from long-read sequencing, which was not appreciated previously in short-read sequencing, was that intron 2 retention co-segregated with E7a inclusion in Mbnl1^−/−^ mice (Supplementary Fig. 3d, e).

In contrast, in homozygous ADR mice, all *Clcn*1 transcripts are eligible for NMD, due to the frameshift mutation in exon 18^13^. Under this condition, turnover across all *Clcn*1 splice products is expected to normalize, and transcript populations at steady state should more closely mirror spliceosome output. Despite the presence of normal Mbnl1, ADR mice exhibited high levels of intron 2 retention and moderate E7a inclusion relative to WT controls (Fig. 2b, c). However, unlike Mbnl1^−/−^ samples where these events mainly co-occurred, the frequency of these inclusion or retention events was largely independent of each other (Supplementary Fig. 3d, e).

Together, these findings support a model where basal output of mRNAs with intron 2 retention is surprisingly high, estimated at ~60% from ADR data, but strongly suppressed at steady state in WT mice by NMD. In Mbnl1^−/−^ mice the relative frequency of transcripts retaining intron 2 is strongly suppressed in transcripts that skip E7a, analogous to WT, but not in transcripts that include E7a, analogous to ADR. Consistent with this model, homozygous deletion of E7a in Mbnl1^−/−^ mice reverted all splice events to WT patterns, presumably by restoring normal patterns of NMD surveillance (Fig. 2b, c). These results substantiate the role of E7a splicing as a lynchpin splice event regulating Clcn1 expression.

### Exon 7a deletion restored ClC-1 channel function and eliminated myotonia in Mbnl1^−/−^mice

**Fig. 3:**
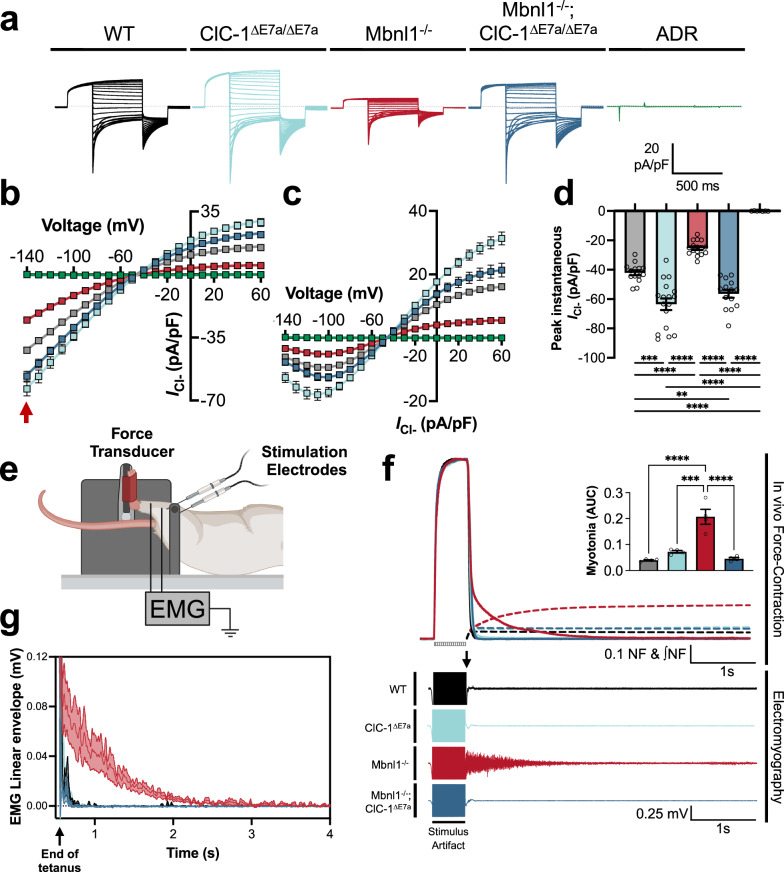
Forced exclusion of exon 7a in Mbnl1^−/−^ mice corrects ClC-1 channel function to WT levels and eliminates myotonia. **a** Whole-cell patch-clamp recordings of ClC-1 currents from FDB myofibers isolated from P18 WT (*n* = 14 fibers), ADR (*n* = 7), ClC-1^∆E7a/∆E7a^ (*n* = 17), Mbnl1^−/−^ (*n* = 16), and Mbnl1^−/−^; ClC-1^∆E7a/∆E7a^ mice (*n* = 14), representative traces shown. **b** IV-plot of instantaneous current-density, shown mean (square symbol) ± SEM. Connecting line represents fit to modified-Boltzmann equation. **c** IV-plot of steady-state current-density, shown mean (square symbol) ± SEM. **d** Peak inward instantaneous current-density at -140  mV (red arrow in **b**). Bars presented mean ± SEM with each myofiber value overlying as dots. Statistical comparison with Brown–Forsythe ANOVA test with two-sided post-hoc Dunnett’s T3 multiple comparisons test. **e** Schematic of experimental setup for collection of paired plantarflexion force recordings and electromyograms (EMGs) from anesthetized mice. Created in BioRender. Sipple, M. (2026) https://BioRender.com/ynwugne. **f** Representative normalized force traces from WT, ClC-1^∆E7a/∆E7a^, Mbnl1^−/−^, and Mbnl1^−/−^; ClC-1^∆E7a/∆E7a^ mice are shown in response to a 500 ms (150 Hz) stimulus (represented by the box with vertical lines). Integration of the force-time curve after stimulus shown by dashed lines. Area under the curve was compared for each group (*n* = 4 mice/genotype, inset). Statistical comparison with a one-way ANOVA with two-sided Tukey’s post-hoc test. Bars shown mean ± SEM with replicates overlying as dots. Representative EMG recordings shown below the force traces with stimulus signal indicated on graph and magnitude maximum at ±0.5 mV to provide adequate scale for visualization of post-stimulus electrical activity. Scales as shown. **g** Averaged linear envelope of EMG (n = 4 mice/genotype). Arrow indicates the approximate alignment in time as indicated by the arrow on the representative traces in **f**, time starting at 0.5 ms (the end of the tetanic stimuli). Data shown as mean (solid line) ± SEM as shown by (dotted lines with shading within ± 1 SEM). Significance on all graphs indicated as multiplicity adjusted *p*-value: ^*^p < 0.05, ^**^p < 0.01, ^***^p < 0.001, and ^****^p < 0.0001. Linear properties and fitting parameters summarized in Supplementary Table 2. Additional statistical details provided in Source Data file.

To determine if genetic removal of E7a was sufficient to recover WT levels of ClC-1 channel function, we collected whole-cell voltage-clamp recordings of ClC-1 currents from myofibers isolated from WT, ClC-1^∆E7a/∆E7a^, Mbnl1^−/−^, and Mbnl1^−/−^; ClC-1^∆E7a/∆E7a^ *flexor digitorum brevis* (FDB) muscles. Representative traces of current densities (current magnitude normalized to size of the surface membrane) collected from fibers at postnatal day 18 are shown in Fig. 3a in response to a standard voltage-clamp protocol^14,30^. The traces display the characteristic voltage-dependent deactivation pattern and inward rectification of ClC-1 channels^13,14,30,31^. Furthermore, by inspecting of both the peak inward instantaneous current as well as the average steady-state current, we were able to assess the current-voltage (I-V) relationships (Fig. 3b, c). Peak instantaneous inward current density at the onset of the hyperpolarization voltage step to −140 mV in Mbnl1^−/−^ myofibers had a significantly reduced current density (−25.3 ± 1.2 pA/pF; *n* = 16 fibers) compared to WT (−42.1 ± 1.7 pA/pF; *n* = 14 fibers; *p* < 0.0001). Whereas the current density of Mbnl1^−/−^; ClC-1^∆E7a/∆E7a^ mice (−56.4 ± 2.7 pA/pF; *n* = 14 fibers) was greater than WT, representing a complete restoration of ClC-1 function. ADR controls displayed negligible ClC-1 current densities (0.03 ± 0.06  pA/pF; *n* = 7 fibers), as expected (Fig. 3d). These findings were consistent with those seen at post-natal day 12 and voltage-dependence of activation was largely independent of genotype^13^. Moreover, ClC-1^∆E7a/∆E7a^ displayed current densities greater than WT across the entire early postnatal period assessed (P7-P18), supporting a “super-rescue” effect of genomic excision of this lynchpin exon (Supplementary Fig. 4).

Observations of cage behavior showed that Mbnl1^−/−^ mice uniformly displayed the characteristic hind limb stiffness of action myotonia when physical stimulation was applied. In contrast, action myotonia was never observed in Mbnl1^−/−^; ClC-1^∆E7a/∆E7a^ mice (qualitative observations during husbandry). Consistent with established literature in myotonia congenita and animals with genetic myotonia, greater than half of ClC-1 function must be lost to induce myotonia; the monoallelic deletion of E7a was sufficient to eliminate all evidence of myotonia in Mbnl1^−/−^; ClC-1^∆E7a/+^ mice. Therefore, subsequent physiologic experiments used heterozygote and homozygote ClC-1^∆E7a^ mice (identified as ClC-1^∆E7a^ or Mbnl1^−/−^; ClC-1^∆E7a^)^32–35^.

To quantify myotonia in vivo, we examined force generation by ankle plantar flexors in anesthetized mice paired with electromyography (EMG). This approach allowed for direct comparison of mechanical myotonia (delayed relaxation following muscle contraction) with electrical myotonia (sustained runs of action potentials in muscle fibers), when both were elicited by stimulating the innervating nerve. Force recordings were collected in response to tetanic stimulation of the tibial nerve, and then a second tetanic stimulus was delivered while recording from subcutaneous electrodes inserted near the surface of the gastrocnemius muscle, as shown by the schematic in Fig. 3e. Mechanical myotonia was quantified by integrating the area under the curve (AUC) of normalized force versus time trace immediately after the cessation of the stimulus. Mbnl1^−/−^ muscle displayed the continued generation of force after the tetani stimulation was terminated, as shown by the representative trace in Fig. 3f, as well as significantly increased AUC values compared to WT, consistent with the presence of mechanical myotonia (Fig. 3f inset, *p* < 0.0001). Mbnl1^−/−^; ClC-1^∆E7a^ force traces swiftly returned to baseline following tetanic stimulation with AUC values equivalent to WT, indicating the complete absence of mechanical myotonia. Continued firing of action potentials after tetani was observed in the Mbnl1^−/−^ muscles, but no activity following the stimulation was seen in Mbnl1^−/−^; ClC-1^∆E7a^ muscles (Fig. 3f lower panel). To better appreciate the differences in the EMG signal amplitudes and patterns, demodulation with a linear envelope approach was completed. There was a clear delay in return to baseline for the Mbnl1^−/−^ recordings and swift return to baseline for Mbnl1^−/−^; ClC-1^∆E7a^ recordings, indicating the presence and absence of electrical myotonia, respectively (Fig. 3g). Overall, the recordings of ClC-1 currents and the paired in vivo force-contraction/EMG experiments indicated that E7a deletion was sufficient to ensure full recovery of ClC-1 channel function and sustained elimination of myotonia in Mbnl1^−/−^ mice.

### Elimination of myotonia improved specific-force production in Mbnl1^−/−^ soleus muscle

**Fig. 4:**
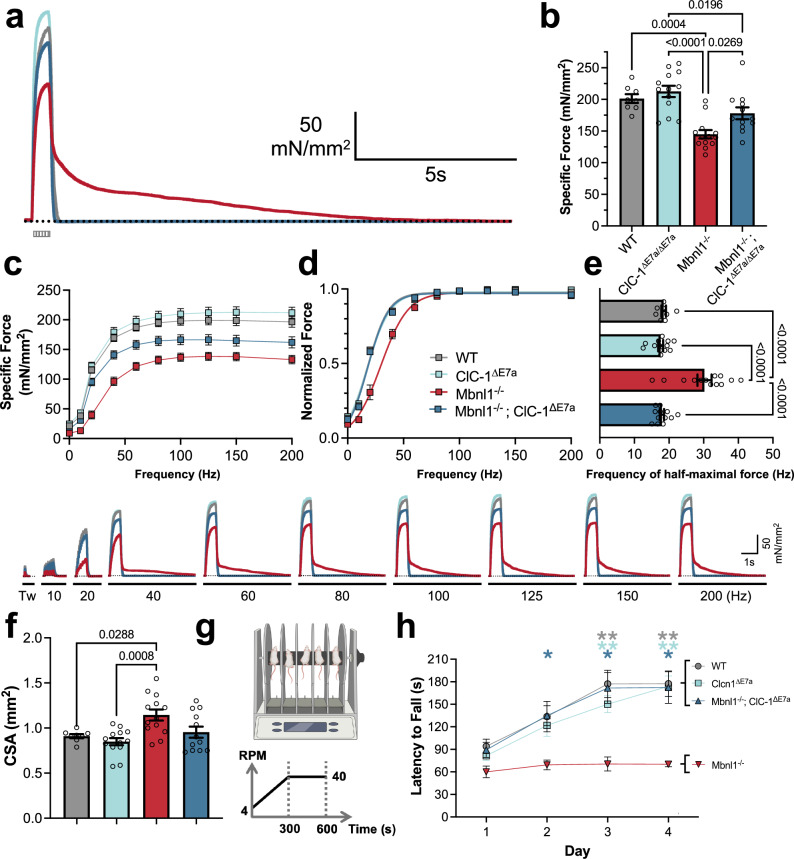
Non-myotonic Mbnl1^−/−^ mice produce increased contractile force compared to myotonic mice. In vitro force-contraction of soleus muscles isolated from 4–6-month-old WT (*n* = 8 muscles), ClC-1^∆E7a/∆E7a^ (*n* = 13), Mbnl1^−/−^ (*n* = 13), and Mbnl1^−/−^; ClC-1^∆E7a/∆E7a^ (*n* = 12) mice. **a** Representative specific force traces in response to a tetanic stimulus (150 Hz; 500 ms), scale as shown. Genotypes represented by colors consistent with the shading in **b**. **b** Peak specific force generated during tetanic stimulation. Data plotted mean ± SEM with individual replicates overlying as dots. **c** Specific force versus frequency curve. Data presented mean ± SEM with connecting lines. Representative specific force traces across stimulation frequencies included below force-frequency graphs. Tw = twitch. Scale as shown. **d** Normalized force versus frequency curve. Data presented mean ± SEM with line representing average fit of the data to a Boltzmann sigmoidal curve. **e** Frequency of half maximal force generation as determined by the Boltzmann sigmoidal fits in **d**. Data presented mean ± SEM with replicates overlying as dots. **f** Cross-sectional areas (CSA) of soleus muscles isolated for force contraction. Data plotted mean ± SEM with individual replicates as dots. **g** Rotarod experiments with a linear acceleration protocol were completed, as previously utilized for the assessment of DM1 mouse models. Created in BioRender. Sipple, M. (2026) https://BioRender.com/u93v863. **h** Average latency to fall during the linear acceleration protocol across four sequential experimental days for WT (*n* = 6 mice), ClC-1^∆E7a/∆E7a^ (*n* = 6), Mbnl1^−/−^ (*n* = 6), and Mbnl1^−/−^; ClC-1^∆E7a/∆E7a^ (*n* = 5). Data shown Mean ± SEM. Analyzed with a repeated measures two-way ANOVA with Tukey’s multiple comparisons. Colored stars above each trial day indicate the multiplicity adjusted *p*-value for comparisons of Mbnl1^−/−^ versus each of the other respective genotypes (as per color), represented as ^*^p < 0.05, ^**^p < 0.01. Other statistical comparisons in **b**, **e**, and **f** represent one-way ANOVA tests with Tukey’s post-hoc test with multiplicity adjusted *p*-values as stated. Additional statistical details provided in Source Data file.

The contribution of myotonia to impaired muscle performance in Mbnl1^−/−^ mice was assessed by ex vivo force-contraction recordings from *solei* isolated from adult (4–6-month-old) WT, ClC-1^∆E7a^, Mbnl1^−/−^, and Mbnl1^−/−^; ClC-1^∆E7a^ mice. Soleus was selected for these experiments, as it has been shown to exhibit more severe myotonia than other hindlimb muscles in the absence of ClC-1^36,37^. Muscles were subjected to various stimulation protocols, including isolated 150 Hz tetanic stimuli (500 ms) designed for maximal activation, as shown by representative traces in Fig. 4a. Peak specific force generated by Mbnl1^−/−^ muscles (144.9 ± 6.7 mN/mm^2^) was 28% less than WT (201.3 ± 7.0 mN/mm^2^; *p* < 0.0004) with a partial recovery in force seen in the Mbnl1^−/−^; ClC-1^∆E7a^ samples (178.0 ± 9.3 mN/mm^2^). Mbnl1^−/−^; ClC-1^∆E7a^ recordings were not significantly different than WT (*p* = 0.29). Additionally, we observed no weakness in the ClC-1^∆E7a^ mice alone (*p* = 0.81; Fig. 4b).

Mirroring in vivo plantarflexion, Mbnl1^−/−^ muscles displayed continued force generation after stimulus termination, consistent with myotonia, that was absent in Mbnl1^−/−^; ClC-1^∆E7a^ samples (Supplementary Fig. 5a, b). Additionally, peak specific force elicited by a single 0.2 ms (twitch) stimulus displayed a similar reduction in specific force output for Mbnl1^−/−^ muscles that was partially recovered Mbnl1^−/−^; ClC-1^∆E7a^ recordings (Supplementary Fig. 5c, d). This indicates that the partial recovery in specific force seen in Mbnl1^−/−^; ClC-1^∆E7a^ muscles is independent of the presence or absence of myotonia.

Moreover, similar trends in specific force output were observed across stimulation frequencies, with a generalized reduction in Mbnl1^−/−^ muscle compared to WT that was partially recovered in Mbnl1^−/−^; ClC-1^∆E7a^ muscle (Fig. 4c). However, when force was normalized, the peak tetanic force produced across stimulation frequencies clearly showed a rightward shift of force-frequency in Mbnl1^−/−^ recordings, indicating a decreased sensitivity to stimulation frequency (i.e., an increased stimulation frequency is required to generate the same fraction of specific force for a given muscle; Fig. 4d). This was quantified by the stimulation frequency required for half maximal force production, which was significantly larger in Mbnl1^−/−^ muscle compared to all other groups (Fig. 4e). Mbnl1^−/−^; ClC-1^∆E7a^ muscles also did not display any evidence of transient weakness during repetitive stimulation protocols, consistent with this being a myotonia dependent phenomenon (Supplementary Fig. 5e).

Overall, the deficits in maximum specific force generation seen in Mbnl1^−/−^ recordings were largely driven by a 26% increase in cross-sectional area (CSA) of Mbnl1^−/−^ solei compared to WT (*p* = 0.029) with similar absolute force output in response to tetanic stimulation (Supplementary Fig. 5f). Interestingly, peak absolute force generated in response to twitch stimulation was decreased in Mbnl1^−/−^ muscles and significantly improved in Mbnl1^−/−^; ClC-1^∆E7a^ muscles (Supplementary Fig. 5g). Furthermore, the change in CSA was not seen in Mbnl1^−/−^; ClC-1^∆E7a^ muscles, and were not significantly different from WT (*p*-adj. = 0.95; Fig. 4f). This indicates that the deficits seen are largely as a result of a decrease in contractile efficiency (force generation per unit area) consistent with altered intrinsic muscle function (e.g., excitation–contraction coupling mechanisms) and/or fiber-type remodeling. Furthermore, these findings align with our prior study Ca_V_1.1^∆E29^/ADR mice, which displayed significant specific force deficits in hindlimb muscles that were improved with longitudinal treatment with verapamil, a drug that functioned as a potent anti-myotonic agent in these mice^9^.

### Absence of myotonia improved rotarod performance in Mbnl1^−/−^ mice

Overall in vivo muscle function was assessed by rotarod testing with a linear acceleration protocol. This approach is expected to integrate the effects of ClC-1 restoration on overall mobility, as previously utilized to assess Mbnl knockout models (Fig. 4g)^38^. Across the 4 days of trials, WT, ClC-1^∆E7a^, and Mbnl1^−/−^; ClC-1^∆E7a^ mice significantly improved in their latency to fall, remaining on the rotarod for a significantly extended duration compared to myotonic Mbnl1^−/−^ mice. The Mbnl1^−/−^ mice failed to improve and showed a latency to fall that was significantly shorter than WT, ClC-1^∆E7a^, and Mbnl1^−/−^; ClC-1^∆E7a^ mice (Fig. 4h), which highlights the functional impairment caused by myotonia in these animals. Notably, Mbnl1^−/−^; ClC-1^∆E7a^ mice performed equivalent to WT in this assay, indicating that deficits seen in Mbnl1^−/−^ mice and the consequent improvement in Mbnl1^−/−^; ClC-1^∆E7a^ mice were due to changes in intrinsic muscle function by elimination of myotonia alone.

### Absence of myotonia reduced DM1 histopathological alterations in muscle

Skeletal muscle in DM1 mouse models exhibits numerous histopathologic features similar to those seen in DM1 patients, including pronounced alterations in fiber type composition and increased central nucleation, albeit milder than what is observed in DM1 patients^3,23,39^. Mbnl1^−/−^ mice exhibit several histopathologic changes in skeletal muscle, including increased central nuclei and changes of fiber type distribution^3,36^.

**Fig. 5:**
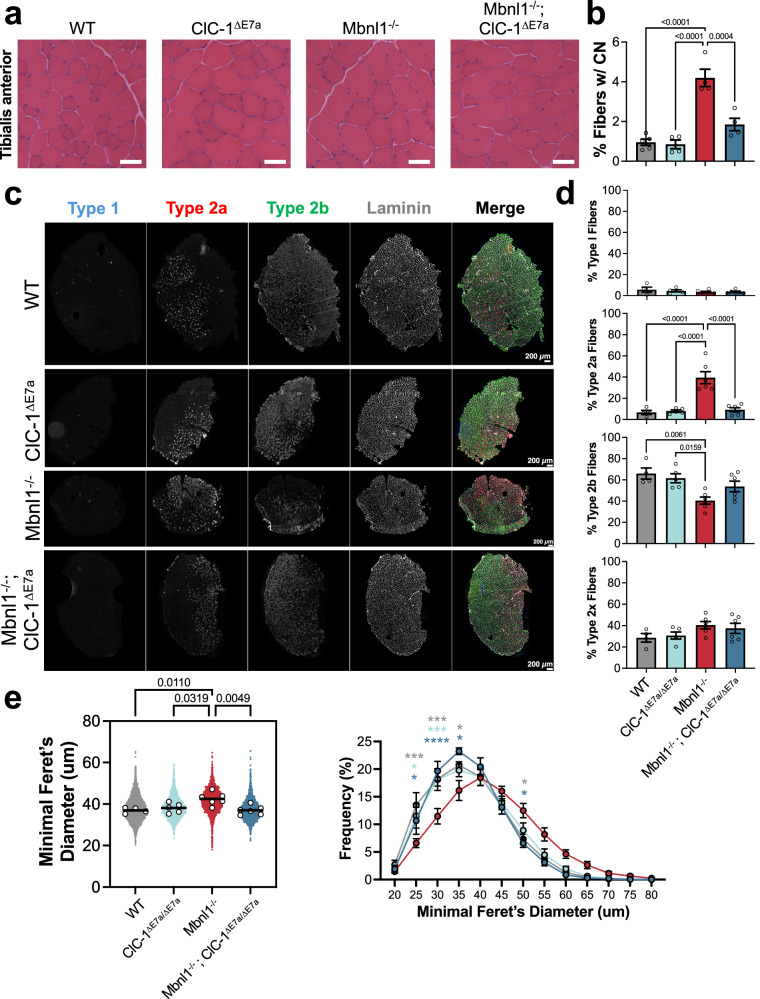
Myotonia is a driver of histopathologic changes in Mbnl1^−/−^ skeletal muscle. **a** Representative images of *tibialis anterior* transverse sections stained with hematoxylin and eosin (H&E) from WT (*n* = 5 mice), ClC-1^∆E7a/∆E7a^ (*n* = 4), Mbnl1^−/−^ (*n* = 4), and Mbnl1^−/−^; ClC-1^∆E7a/∆E7a^ (*n* = 4) mice (4–6-month-old). Scale bars shown represent 50 micrometers. **b** Percent of fibers with central nuclei present on H&E stained tibialis anterior samples. Data presented as mean ± SEM with individual values. **c** Fiber-typing by myosin heavy chain (MyHC) isoform was completed on WT (*n* = 4 mice), ClC-1^∆E7a/∆E7a^ (*n* = 5), Mbnl1^−/−^ (*n* = 6), and Mbnl1^−/−^; ClC-1^∆E7a/∆E7a^ (*n* = 6) mice by immunofluorescence, representative images shown, with each MyHC isoform channel and laminin counterstain shown in grayscale with the merged image pseudo-colored (based on color of channel labels). **d** Percent of fibers staining for each of the respective MyHC isoforms. Data presented as mean ± SEM with individual values. **e** Fiber morphometry was completed based on laminin staining with minimum Feret’s diameter determined for each fiber plotted as a scatter plot (left) and histogram (right). For the scatter plot, small dots represent all fibers analyzed across biological replicates, whereas larger dots filled in white represent mean of each biological replicate. Bar represents mean. Statistical comparisons in **b**, **d**, and (**e**, left) represent one-way ANOVAs with Tukey’s multiple comparison test. Multiplicity adjusted *p*-values of significant comparisons as stated. Comparisons in (**e**, right) represents a two-way ANOVA assessing the effects of genotype and minimal Feret’s diameter on the frequency distribution with Tukey’s multiple comparisons. Stars above histograms represent multiplicity adjusted *p*-values of significant comparisons between the genotype associated with the star color and Mbnl1^−/−^ mice for that bin, as no comparisons between the other groups were found to be significant: ^*^p < 0.05, ^***^p < 0.001, and ^****^p < 0.0001. Additional statistical details provided in Source Data file.

To assess the role of myotonia as a driver for histologic alterations, we examined samples from hindlimb and diaphragm muscles of 4–6-month-old mice. Representative sections of tibialis anterior (TA) muscle are shown in Fig. 5a. Compared to WT controls, the percentage of muscle fibers with central nuclei was higher in Mbnl1^−/−^ (4.2%) than Mbnl1^−/−^; ClC-1^∆E7a^ mice (1.8%, 56% reduction, *p* = 0.0004, Fig. 5b), and similar effects were observed in quadriceps (Supplementary Fig. 6a, b). To examine fiber type distributions, we performed immunofluorescence (IF) and quantified minimum Feret’s diameter for type I (Myh7), type 2a (Myh2), and type 2b (Myh4) myofibers. Representative IF of TA transverse sections are shown in Fig. 5c. As recently reported, Mbnl1^−/−^ muscles showed a significant increase in type 2a (fast-twitch oxidative) and concomitant decrease of type 2b (fast-twitch glycolytic) fibers^36^. Importantly, this shift in fiber type distribution did not occur in Mbnl1^−/−^; ClC-1^∆E7a^ samples. Furthermore, no changes in the percentage of type 1 (slow twitch) or type 2x (fast glycolytic) fibers were observed (Fig. 5d). Examination of fiber type distribution in gastrocnemius and quadriceps showed similar results (Supplementary Figs. 6 and 7). In agreement with a recent study, shifts in fiber type distribution were not observed in diaphragm (Supplementary Fig. 7a)^36^, a muscle that displays less severe chloride channel myotonia compared to appendicular skeletal muscles^9,37^. These findings also parallel those seen in hindlimb muscles of non-dystrophic ADR mice^9^.

Additionally, we observed a significant increase in the Feret’s minimum diameter in the Mbnl1^−/−^ TA, whereas Mbnl1^−/−^; ClC-1^∆E7a^ samples were similar to WT (Fig. 5e). When muscle fiber morphometry was analyzed in specific fiber-types, it was shown that the increase in the Feret’s minimum diameter was predominantly seen across type 2 fiber subtypes (Supplementary Fig. 7b). These findings align with the myotonia-dependent and reversable myofiber hypertrophy previously seen *HSA*^LR^; Mbnl1^−/−^ muscles^23^. Additionally, ClC-1^∆E7a^ control samples were near WT for all parameters assessed, including percent of fibers with central nuclei, fiber-type distributions, and fiber minimum Feret’s diameter, across all muscle groups tested (Fig. 5, and Supplementary Figs. 6 and  7).

### Myotonia drives changes in transcript expression in skeletal muscle of Mbnl1^−/−^ mice

**Fig. 6:**
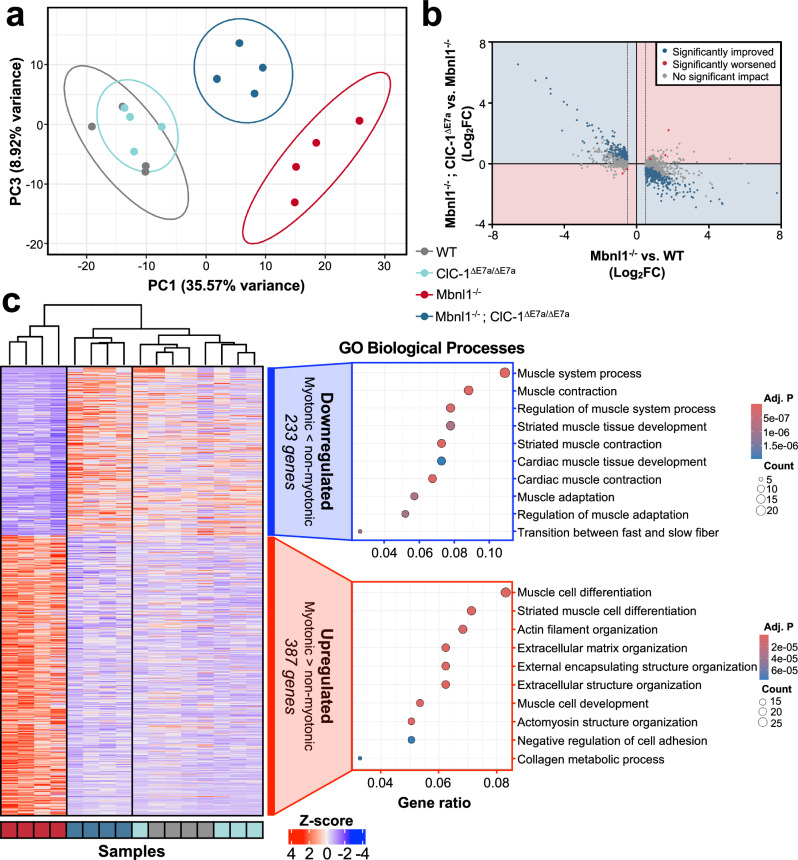
Myotonia alters transcript expression in Mbnl1^−/−^ mice. Deep RNA sequencing completed on quadriceps muscle isolated from 4–6-month-old WT, ClC-1^∆E7a/∆E7a^, Mbnl1^−/−^, and Mbnl1^−/−^; ClC-1^∆E7a/∆E7a^ mice (*n* = 4 mice/genotype). **a** Principal component analysis (PCA) of gene expression was completed based on the top 500 genes with the greatest variance with samples plotted against PC1 and PC3 (PC2 excluded as top loading genes represented predominantly those localized to sex chromosomes). Ellipses represent 95% confidence boundary of group clusters. **b** Genes with altered expression in Mbnl1^−/−^ compared to WT samples (log_2_FC ⎸> 0.5 and DESeq2 adj. *p* < 0.05), defined the subset of genes with altered expression in the absence of Mbnl1 (1983 genes). Direct comparison between Mbnl1^−/−^; ClC-1^∆E7a/∆E7a^ and Mbnl1^−/−^ was then utilized to determine if expression was significantly (adj. *p* < 0.05) improved or worsened in the absence of myotonia. For visualization, genes with altered expression in Mbnl1^−/−^ compared to WT samples, were plotted by log_2_FC of Mbnl1^−/−^ vs. WT against log_2_FC of Mbnl1^−/−^; ClC-1^∆E7a/∆E7a^ vs. Mbnl1^−/−^, and thus genes in the second and forth quadrants represent those with expression moved closer to WT in Mbnl1^−/−^; ClC-1^∆E7a/∆E7a^ compared to Mbnl1^−/−^ samples. Gray dots represent genes without significant improvement or worsening of expression in Mbnl1^−/−^; ClC-1^∆E7a/∆E7a^ compared to Mbnl1^−/−^ samples. Blue dots represent those with significantly improved expression (464 genes) and red dots represent those with significantly farther from WT (5 genes). Differential expression assessed by DESeq2 with BF adjusted *P*-values (<0.05). **c** Direct comparison of the differential expression between Mbnl1^−/−^ and Mbnl1^−/−^; ClC-1^∆E7a/∆E7a^ mice yielded 620 differentially expressed genes (log_2_FC ⎸> 0.5 and DESeq2 adj. *p* < 0.05) with a heatmap of the normalized expression of these genes shown for all samples. Based on the expression of these genes, unsupervised sorting of the samples was completed with the Mbnl1^−/−^ and Mbnl1^−/−^; ClC-1^∆E7a/∆E7a^ clustering in isolated groups and control samples (WT and ClC-1^∆E7a/∆E7a^) co-clustering. Gene ontology analysis of biological processes represented in the downregulated and upregulated genes in Mbnl1^−/−^ versus Mbnl1^−/−^; ClC-1^∆E7a/∆E7a^ samples was completed. Scales and significance levels as shown. Additional gene ontology results provided in Source Data file.

Emphasis is often placed on splicing dysregulation in DM1 muscle, but there are also extensive changes in gene expression^40–43^. Mechanistic hypotheses have ranged from direct interaction of transcripts with toxic RNA repeats to RNA interference and secondary effects of mis-regulation of genome-organizing nuclear envelope transmembrane proteins^40,44,45^. It is noteworthy, however, that a previous study of DM1 models showed that 24% of changes in gene expression also occurred in ADR mice^40^, raising the possibility that some changes in gene expression in DM1 are driven by myotonia itself. We tested this directly by comparing changes of transcript expression between myotonic (Mbnl1^−/−^) and non-myotonic (Mbnl1^−/−^; ClC-1^∆E7a/∆E7a^) DM1 mice using deep RNAseq (150 bp paired-end reads; 66.3 ± 8.3 million unique reads (mean ± IQR) of total RNA isolated from quadriceps muscle from 4–6-month-old WT, ClC-1^∆E7a/∆E7a^, Mbnl1^−/−^, and Mbnl1^−/−^; ClC-1^∆E7a/∆E7a^ mice (*n* = 4 mice/genotype). Normalized counts of edited genes (*Mbnl*1 and *Clcn*1) were inspected to provide internal validity of sequencing results (Supplementary Fig. 8a, b). Principal component analysis (PCA) of 500 genes with the highest variance across all samples revealed co-clustering of WT and ClC-1^∆E7a/∆E7a^ samples (circles represent 95% Confidence Index), as well as two distinct clusters for Mbnl1^−/−^ and Mbnl1^−/−^; ClC-1^∆E7a/∆E7a^ samples. Mbnl1^−/−^ samples were localized furthest from WT along PC1, with Mbnl1^−/−^; ClC-1^∆E7a/∆E7a^ samples being intermediate. PC1 was plotted against PC3, as this provided the best clustering across our experimental groups, as sex distributions were unbalanced in Mbnl1^−/−^ and Mbnl1^−/−^; ClC-1^∆E7a/∆E7a^ groups and PC2 was predominantly loaded with genes on sex chromosomes (Fig. 6a, Supplementary Fig. 8c–e). Gene ontology (GO) analysis of genes with highest representation in PC1 was strongly enriched for the skeletal muscle contraction pathway (Supplementary Fig. 8e, f).

Differential expression analysis revealed 1983 genes with altered expression in Mbnl1^−/−^ muscle compared to WT (∣log_2_FC∣ > 0.5, *p*-adj. < 0.05). Of these genes, expression was significantly closer to WT in Mbnl1^−/−^; ClC-1^∆E7a/∆E7a^ samples for 464 genes (Mbnl1^−/−^; ClC-1^∆E7a/∆E7a^ vs Mbnl1^−/−^, *p*-adj. < 0.05), and thus indicates that myotonia plays a significant role in driving expression changes for 23% of genes dysregulated in Mbnl1^−/−^ mice.

The top biological process in genes that were downregulated in Mbnl1^−/−^ muscle compared to Mbnl1^−/−^; ClC-1^∆E7a/∆E7a^ was related to skeletal muscle contraction. The main driver of this alteration was reduced expression of genes that are markers for the type 1 fiber program, comprised mainly of myosin heavy or light chains and other components of the contractile apparatus (*Myh6, Myh7, Myh7b, Myl2, Tnnc*1*, Tnnt*1*, Tnni*1*, Tpm3*) in myotonic muscle^46,47^. Notably, Mbnl1^−/−^ muscle showed concurrent upregulation of the type 2x fiber marker, *Myh*1, which was also closer to WT in Mbnl1^−/−^; ClC-1^∆E7a/∆E7a^ mice. Together with results of fiber typing above, these findings support the concept that myotonia drives calcium-mediated remodeling of muscle fibers. Furthermore, this aligns with the previous observation in ADR mice that reprogramming of muscle by myotonia is not simply a unidirectional shift from fast to slow fiber types but rather a convergence on the 2a/2x program from both ends of the fiber type spectrum (type 1 and type 2b)^48^.

In contrast, the biological process most upregulated in Mbnl1^−/−^ compared to Mbnl1^−/−^; ClC-1^∆E7a/∆E7a^ samples was for skeletal muscle differentiation (*Cacnb4*), genes involved in cellular signaling (*Camk2d* and *Popdc3*), and several genes encoding proteins important for skeletal muscle cytoskeletal, extracellular matrix, and sarcomere structure (*Flnc*, *Myom2*, *Col*1*4a*1, and *Tmod2*). GO analysis also identified dysregulation of genes encoding many ion channels, indicating possible compensatory regulation of skeletal muscle excitability in the setting of ClC-1 loss (included in Source Data). Taken together, these results provide direct confirmation that myotonia plays an important role in the dysregulation of gene expression in Mbnl1^−/−^ muscle, with implications for muscle function and differentiation.

### Removal of myotonia reverses mis-splicing of a specific set of genes in muscle

**Fig. 7:**
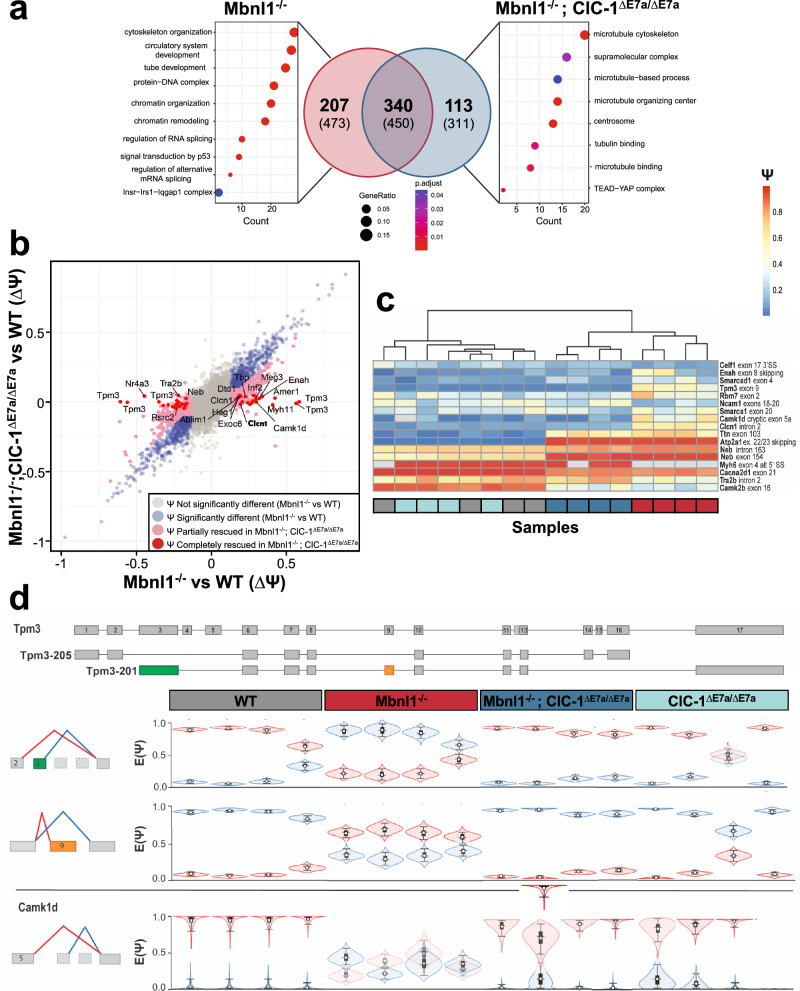
Myotonia regulates specific splicing events. Splicing analysis of deep RNA sequencing data collected for quadriceps muscle isolated from 4–6-month-old WT, ClC-1^∆E7a/∆E7a^, Mbnl1^−/−^, and Mbnl1^−/−^; ClC-1^∆E7a/∆E7a^ mice (*n* = 4 mice/genotype). **a** Overlap of genes mis-spliced in muscle of Mbnl1^−/−^ and Mbnl1^−/−^; ClC-1^∆E7a/∆E7a^ mice compared to WT mice (MAJIQ output FDR < 0.1, DPSI > 0.1 & < -0.1). Unique genes were analyzed regarding biological pathway (dot plot, top 10 GO terms). **b** Splicing events in Mbnl1^−/−^ and Mbnl1^−/−^; ClC-1^∆E7a/∆E7a^ were each compared to WT mice and the PSI-values were plotted as a scatter plot (dots represents alternative splice events (ASEs) with some transcripts having multiple ASEs). ASEs that were equivalent in Mbnl1^−/−^ and WT are displayed in grey, while ASEs with DPSI-value above 0.1 and below -0.1 and an FDR < 0.2 are shown in blue. Pink ASEs were partially rescued, defined as more than a 0.15 PSI shift in Mbnl1^−/−^; ClC-1^∆E7a/∆E7a^ towards WT. ASEs completely rescued (altered in Mbnl1^−/−^ vs WT and non-altered in Mbnl1^−/−^; ClC-1^∆E7a/∆E7a^ vs WT at an FDR < 0.05) are shown in red with gene labels. **c** PSI heatmap of selected genes ranging from complete rescue to unaffected. Rows represent individual ASEs and columns represent biological replicates arranged by unsupervised sorting (genotype denoted by colored box below). PSI per scale bar shown. Gray represents ASEs not detected in the sample. **d** Tpm3 shows the strongest rescue in mice without myotonia. In Mbnl1^−/−^ mice, exon 3 and exon 9 are included with a PSI-value of 0.65 and 0.7, respectively. Camk1d inclusion of a cryptic exon 5a was also observed at a higher PSI in Mbnl1^−/−^ samples and was reduced in Mbnl1^−/−^; ClC-1^∆E7a/∆E7a^ samples. The Camk1d splice event was only detected in 3 of the ClC-1^∆E7a/∆E7a^ samples. Shown as violin plot of E(PSI) colored based on schematic left with superimposed box plot with a box that extends from the 25 to the 75 percentiles, a horizontal line at the median, tails that represent the 10 and 90 percentiles, and a white circle at the mean for each biological replicate.

Previously, we used targeted RNA-seq to examine a group of splice events that are strongly dysregulated DM1 models and observed that 20% also occurred in ADR mice^49^. Accordingly, we wanted to obtain a transcriptome-wide view of the impact of myotonia on alternative splicing specifically in the absence of Mbnl1. We used a splicing analysis tool, MAJIQ, that detects simple (e.g., exon skipping, intron retention) and complex splicing events (e.g., triple exon skipping versus intron retention)^50^, to analyze the RNA-seq data described above. Pairwise comparisons of Mbnl1^−/−^ and Mbnl1^−/−^; ClC-1^∆E7a/∆E7a^ muscle with WT controls identified a higher number of dysregulated splicing events in myotonic (923 events across 547 genes) than non-myotonic Mbnl1^−/−^; ClC-1^∆E7a/∆E7a^ muscle (761 events across 453 genes), applying thresholds of a change in “percent splicing in” (ΔPSI) > 0.1 or < -0.1 with a false discovery rate (FDR) < 0.05. Comparative analysis of affected genes and splicing events revealed that 52% of genes were shared between both conditions (Fig. 7a). However, Mbnl1^−/−^ mice exhibited a greater number of unique (unshared) splicing events (473 across 207 genes) than Mbnl1^−/−^; ClC-1^∆E7a//∆E7a^ mice (311 unique events across 113 genes).

Pathway analysis indicated that the splice events affected in myotonic but not in non-myotonic Mbnl1^−/−^ mice were enriched for splicing regulators, such as *Srsf6*, *Prpf39*, *Rbm7*, *Tra2b*, *Rbfox2*, and *Hnrnpu*, and pathways involved in cytoskeleton and chromatin organization (Fig. 7a, left dot plot, and Fig. 7b). In contrast, the genes uniquely mis-spliced in non-myotonic Mbnl1^−/−^ mice were predominantly associated with cytoskeleton organization and the Yap-Tead complex, the latter comprising the transcription factor YAP1 and its coactivator TEAD1 that are implicated in cell proliferation, survival, and tissue growth (Fig. 7a, right dot plot, and Fig. 7b)^51^.

### Rescue of mis-splicing in Mbnl1^−/−^; ClC-1^∆E7a/∆E7a^ mice

We classified splice events as completely rescued by E7a deletion in cases where splicing events in Mbnl1^−/−^; ClC-1^∆E7a/∆E7a^ muscle were not different from WT and Mbnl1^−/−^ muscle exhibited a shift of at least ± 0.1 ΔPSI (shown in red in Fig. 7b, FDR < 0.05), or as partially rescued when ΔPSI shifted towards WT by at least 0.15 (shown in pink in Fig. 7b, FDR < 0.2). The heatmap in Fig. 7c illustrates PSI values for selected splicing events that remain unchanged between myotonic and non-myotonic Mbnl1^−/−^ muscle (*Atp2a*1*, Neb, Ttn, Cacna2d*1*, Camk2b*), as well as some that exhibit partial or complete rescue. Despite our previous work demonstrating synthetic effects of myotonia with Ca_V_1.1 exon 29 exclusion, Mbnl1^−/−^ muscle displays minimal mis-splicing of *Cacna*1*s* (>90% PSI); thus, limiting our ability to probe the role of myotonia on this specific splice event (Supplementary Fig. 9). However, unsupervised clustering of these events further supports the concept that Mbnl1^−/−^; ClC-1^∆E7a/∆E7a^ mice represent an intermediate state between myotonic Mbnl1^−/−^ and WT mice (Fig. 7c). An interesting example of rescue concerns tropomyosin 3 (Tpm3), whose expression was downregulated (-2.7 log_2_FC) in Mnbl1^−/−^ mice and exhibited three splicing events that differed between Mbnl1^−/−^ and WT muscle with large effects (ΔPSI up to 0.7) and strong confidence (FDR < 0.05). *Tpm3* exons 3 and 9 were preferentially included in Mbnl1^−/−^ mice, whereas exons 1, 2, and 14 were predominantly used in WT. All splicing changes in Tpm3 were fully rescued by E7a deletion, as shown for selected exons in the upper panel of Fig. 7d. Camk1d also showed strong rescue (Fig. 7d, lower panel). Similarly, the genes *Enah* and *Ablim*1, which are frequently mis-spliced in DM1 patients^10,52^, displayed complete rescue of at least one misregulated splicing event (Supplementary Fig. 9). Taken together, these findings support the idea that myotonia contributes to splicing dysregulation in DM1 models but also raises the possibility that myotonia may mitigate dysregulation for a limited number of particular events.

## Discussion

We demonstrated that CRISPR-mediated excision of the E7a sequence from the *Clcn*1 gene was sufficient to restore ClC-1 channel function and eliminate myotonia from the Mbnl1^−/−^ model of DM1. Elimination of myotonia resulted in a significant decrease in the overt manifestations of myopathy, including and potentially most clinically relevant, significant improvements in force production. Moreover, we also presented (i) the utility of targeted long-read sequencing to assess Mbnl1-dependent transcript isoform changes for a gene with complex splice patterning, (ii) a method for the assessment of mechanical and electrical myotonia in DM1 mouse models longitudinally, and (iii) a comprehensive analysis of the histopathological and transcriptomic impact of myotonia in skeletal muscle of DM1 mouse models.

Targeted long-read (PacBio/ONT) sequencing is emerging as useful technique for probing RNA processing and unique transcript isoforms for genes with complex splice patterning^53^. In the field of neuromuscular diseases and specifically in DM1, third-generation sequencing has primarily focused on genomic DNA and used to improve genetic diagnosis and characterization of expanded repeats; for example, efforts to better understand interruptions in the repetitive sequence^54,55^. Here, we leveraged long-read sequencing to better characterize changes in *Clcn*1 transcript isoform expression in the absence of Mbnl1 protein.

Over the past two decades since the implication of ClC-1 loss as the cause of myotonia in DM1, it has been shown that several variant *Clcn*1 isoforms, in addition to splice products that include E7a, were upregulated in DM1 patients and disease models, including transcripts with intron 2 retention^15^. In fact, a cell-based screen was developed, and a high throughput screen carried out, with the sole objective of identifying small molecules that directly repress intron 2 inclusion for the treatment of DM1 myotonia. This prompted the question, are these other splicing events independently regulated by (1) multiple cognate elements for Mbnl1 binding along the primary *Clcn*1 transcript, (2) cis-acting elements of E7a splicing or (3) byproducts of changes in the overall stability of *Clcn*1 transcripts that unmask the fidelity of *Clcn*1 transcript outputs from the spliceosome?

In this study, and in agreement with previous studies, we demonstrate that loss of Mbnl1 activity leads to decreased steady-state *Clcn*1 mRNA. This is thought to be driven largely by decreased mRNA stability stemming from the aberrant inclusion of E7a, causing a frameshift, and ultimately NMD of transcripts^13^. As E7a inclusion rises, the fraction *Clcn*1 splice products that are NMD substrates increases, thereby normalizing the turnover rate across all variant isoforms and unmasking the proportional outputs from the spliceosome. Moreover, the presence of a second NMD-causing splicing change (e.g., the simultaneous intron 2 retention and E7a inclusion in a single transcript) does not significantly alter the stability of the transcript more than a single frameshifting aberrant splice-event. This was supported by both the large increase in intron 2 retention seen in ADR samples, in which all transcripts possess at least one NMD-yielding event, as well as the relatively more abundant intron 2 retention in E7a-containing transcripts in Mbnl1^−/−^ samples (Supplementary Fig. 3d, e). Overall, these results support the conclusion that E7a inclusion is the primary event regulated by Mbnl1 of mouse *Clcn*1 transcripts. Other observed events are likely the consequence of changes in overall transcript stability, with limited evidence in support of either multiple sites of Mbnl1 activity or E7a playing a role as a cis-acting element directing distant splicing events in the transcript^27^. There is some evidence that elements near E7a may impact neighboring exon/intron splicing that needs further interrogation. Ultimately, this raises the importance of sequencing transcripts with complex splicing patterning end-to-end, rather than individual splice events in isolation, when interrogating the effects of splice-shifting therapeutics.

Furthermore, with clear links between transcript splicing and phenotypic consequences, myotonia remains a key symptom and phenotype used to assess the severity of the DM1 disease state as well as evaluate the success of therapeutics in patients and disease models^8,56–58^. However, many commonly used assays lack methods of objective quantification and longitudinal monitoring and do not fully assess both mechanical and electrical myotonia despite many efforts to develop assays that reliably quantify the severity of myotonia^59^. Here, we utilized both in vivo plantarflexion and paired posterior compartment subcutaneous EMG to provide methods for quantifying both mechanical and electrical force production. This technique provides an opportunity for further longitudinal studies tracking responses to therapeutic interventions as well as potentially providing further insight into emerging pathophysiological phenomenon, such as silent myotonia, sustained after-depolarizations and muscle weakness, in myotonic disorders^60^.

Through deep RNA-seq, we were able to directly assess the impact of myotonia on transcript expression and splicing in mice lacking functional Mbnl1. We observed a significant shift in overall transcript expression between myotonic and non-myotonic Mbnl1^−/−^ mice with ~25% of transcripts with altered expression in Mbnl1^−/−^ muscles having expression levels closer to WT in Mbnl1^−/−^; ClC-1^∆E7a^ samples. Moreover, of the genes that featured significantly different expression levels between myotonic and non-myotonic Mbnl1^−/−^ muscles, the biological pathways most significantly enriched were muscle contraction and differentiation, further substantiating the role of myotonia in muscle DM1 weakness. Interestingly, another molecular function that featured substantial differences was in other ion channels on the sarcolemma, including several potassium channels and their subunits (*Kcnab*1/*Kcnh7*/*Kcnk3*/*Kcng4*/*Kcnb*1/*Kcnk2*). This suggests the potential role of compensatory changes in the expression of other ion channels to modulate or dampen myotonia in DM1. These findings are important for consideration when studying alternative targets for the treatment of myotonic disorders, such as previously completed with the use of potassium channel agonists in myotonia congenita mouse models^61–63^. We also observed changes in splicing for several transcripts in the setting of the absence of myotonia in Mbnl1^−/−^ mice, including tropomyosin isoforms, indicating a potential role of myotonia for regulating splicing changes in DM1. The mechanism for this remains to be elucidated; however, as these experiments were completed in mice deficient in functional Mbnl1 and absent of toxic-CUG repeats, these findings represent a process independent of functional Mbnl1 or interactions with the toxic RNA. Notably, broader mis-regulation of splicing factors has been demonstrated in primary muscle cells isolated from people with DM1^64^ and in Mbnl1^−/−^ muscle with, but not in those without myotonia.

In this study, myotonia was found to play a previously undescribed role in orchestrating changes in splicing for several transcripts in DM1 mouse models. Tropomyosins are two-stranded, α-helical coiled-coil proteins that polymerize head-to-tail along actin filaments, regulating actin stability and function (reviewed in ref. ^65^). Each *Tpm* gene (*Tpm*1*-4*) encodes multiple isoforms with distinct expression profiles, localization, and functions^66^. Specifically, *Tpm* genes produce both high-molecular-weight (HMW) and low-molecular-weight (LMW) isoforms, where HMW isoforms serve as muscle-specific variants, while LMW isoforms function primarily in non-muscle cells. *Tpm3* is the predominant slow muscle fiber isoform among tropomyosins, with *Tpm-205* being the major HMW isoform critical for muscle contraction, whereas LMW *Tpm3* isoforms regulate transverse-tubule function and glucose uptake^67,68^. Interestingly, in Mbnl1^−/−^ muscle, *Tpm3* is not only mis-spliced but also significantly downregulated (−2.7 log_2_FC). In Mbnl1^−/−^; ClC-1^∆E7a^ muscle, both splicing and transcriptional expression of *Tpm3* were largely restored (−0.2 log_2_FC). Despite clear ties between cellular excitability and transcript expression in cells such as neurons through the process known as excitation-transcription coupling^69^, the mechanism by which cellular excitability exerts changes in splice patterning remains unknown and thus warrants further studies into the process of excitation-splicing coupling.

*Limitations* of our study revolve around the use of a mouse model with mild to moderate DM1 phenotype. While the elimination of myotonia had striking effects on mRNA expression and processing as well as muscle histology that resulted in muscle function that resembled that of a healthy control, it remains unknown whether targeting myotonia would have as pronounced of a benefit in a model for severe DM1. Moreover, this highlights a limitation of studying myopathy, specifically muscle atrophy, in mouse models of DM1, as even more severe models such as the Mbnl1^−/−^; Mbnl2 ^−/+^ display relative muscle hypertrophy, not seen in DM1 patients^36^.

We chose Mbnl1^−/−^ mice for this study as it was the most straightforward model to study the effect of myotonia on DM1 myopathy without complex breeding schemes or concerns that myotonia could influence CUG^exp^ transcript expression, confounding results, as seen previously^23^. Further, Mbnl1 loss has been shown to account for the majority of alternative splicing changes and most of the effects of gene expression when compared to the *HSA*^LR20b^ mouse model^49,70^. Future studies should be performed in DM1 models that fully recapitulate the mechanism through expression of larger CUG^exp^ containing transcripts, such as DMSXL mice, or more severe muscle phenotype, such as mice with compound loss of Mbnl1 and Mbnl2, to determine the extent that myotonia shapes the myopathy^23,49,71^.

Our findings also illuminate a limitation of extrapolating findings from murine models to DM1 patients, as many of the existing models regardless of apparent severity of phenotypic presentation feature findings, such as muscle hypertrophy, that do not fully recapitulate the skeletal muscle sequelae of DM1^36^. Despite these limitations, the numerous murine models of DM1 have served integral roles in interrogating disease mechanisms and preclinical studies investigating novel therapeutic approaches to DM1, and thus insights gained through these approaches warrant further clinical studies.

Overall, the work presented here lays the foundation for establishing a pathologic role of myotonia in the development of myopathy in myotonic dystrophy (type 1 and 2) and provide initial indications that long-term removal of myotonia from myotonic dystrophy may prevent some of the progressive myopathy seen in the disease. Accordingly, further pre-clinical studies utilizing clinically available anti-myotonic agents or even clinical trials with longer-term follow-up are needed to fully ascertain whether these changes will hold true in settings more tangible to clinical practice. Several sodium-channel blockers, including mexiletine and ranolazine, have been utilized clinically across myotonic disorders, as they are generally well tolerated and provide significant improvements in myotonia^72–75^. However, patients with DM1 were the least likely to have trialed anti-myotonic therapies across the genetic myotonias surveyed, with only 27% of DM1 patients surveyed having attempted treatment with any anti-myotonic agent^76^. Although, this study did not provide direct evidence to account for this discrepancy, it remains plausible that if a disease modifying role of anti-myotonic medications is established, further use of these available medications could provide added benefits to DM1 patients.

Furthermore, in the era of RNA directed therapeutic development, designed to directly target the pathologic processes underlying DM1, myotonia has also emerged as a key trial endpoint due its well described link from splice event to disease symptom. The success of these trials is in part gauged based on the ability for treatments to improve myotonia as the primary endpoint. Evidence presented here suggests abating myotonia has the potential to directly improve myopathy. Further, myotonia may interact with several DM1 cellular processes that may potentiate their influence on muscle health. Indeed, this was recently observed with the synthetic effect of Ca_V_1.1 alternative splicing and myotonia^9^. Thus, clinical outcomes from treatments decreasing toxic RNA burden could be further improved as ClC-1 function is corrected. It remains unknown whether complete remission in myotonia will be achieved by these novel agents, and thus there likely will still be an important role for these anti-myotonic drugs with the potential for combinatorial therapy with additive or even synergistic benefit.

## Methods

### Mouse lines

A transgenic mouse line that prevents the inclusion of the alternate exon 7a (E7a) in Clcn1 transcripts was generated with CRISPR/Cas9 designed for targeted excision of the E7a sequence from the *Clcn*1 gene. Single guide RNAs (sgRNAs) targeted the intronic sequences flanking this alternative exon; this was to ensure complete removal of the splice donor/acceptor sites, as this has been previously shown to be successful to force the exclusion of a targeted exons while not perturbing other splice events in the transcripts^9,77^. Sequences of sgRNAs were 5′- GGAATCGACACAGTTCATGTGGG -3′ and 5′- GAACTTATGGGTGGGCAACCAGG-3′. The mouse line was generated on a C57BL/6J background and extensively backcrossed (>6 generations) with wild-type mice to eliminate possible off-target mutations. Standard PCR with primers flanking the excised region (Supplementary Table 1a) were used for genotyping from genomic DNA and the forward primer was used for Sanger sequencing (Eurofins Scientific) to verify the correct excision from the *Clcn*1 gene. Subsequently, this line was extensively backcrossed (>6 generations) with FVB/N mice, as the model Mbnl1^−/−^ used here is all maintained on a congenic FVB/N background. All other experiments included in this manuscript were completed on mice with a congenic FVB/N background. Homozygous mice lacking exon 7a from both *Clcn*1 alleles are denoted as ClC-1^∆E7a/∆E7a^. For some behavioral and functional experiments, both heterozygote (as ClC-1^∆E7a/+^ and homozygote (as ClC-1^∆E7a/∆E7a^) mice were used, as the excision of only a single allele was shown to be successful in eliminating myotonia; this was denoted as ClC-1^∆E7a^. The Mbnl1-null mouse that was used featured the targeted excision of exon 3 and was extensively characterized previously as a moderate DM1 model featuring skeletal muscle myotonia and increased central nuclei^3^. For simplicity, these mice are indicated as Mbnl1^−/−^. Additionally, for a Clcn1 nonsense-mediated decay (NMD) control mouse model, *adr-mto2J* mice (ADR) were obtained from Jackson Laboratories (Bar Harbor, MA) and extensively backcrossed to the FVB/N background. These mice feature a frameshift mutation in exon 18 of the *Clcn*1 gene that causes a premature termination codon in exon 19—previously shown to induce significant NMD^13^. Standard genotyping methods utilizing genomic DNA isolation and PCR with agarose gel electrophoresis (ClC-1^∆E7a^, Mbnl1^∆E3^) or Sanger sequencing (ADR) were used to confirm all exon deletions and/or mutations (Supplementary Table 1a). Both male and female mice were used for all experiments, as no sex-based differences were shown with these strains in both the previous literature and preliminary experiments with these mice^3,78^. All experiments were completed as outlined by an animal protocol approved by the University of Rochester Committee on Animal Resources (UCAR; equivalent to IACUC) and conform to ARRIVE guidelines. Animals were housed in the university’s vivarium in climate-controlled rooms with micro-isolation employed until the time of experiments with welfare monitoring. Approved forms of euthanasia were used for all terminal experiments.

### RNA isolation

RNA was isolated from quadriceps muscle excised from mice that was immediately frozen in liquid nitrogen and stored at -80˚C. Approximately 50–100 mg of tissue was homogenized using a bead mill (Bead Ruptor, Omni International) with ceramic beads (Fisherbrand) in TRI-reagent (Molecular Research Center) for initial RNA isolation. RNA was then purified (re-isolated) with RNeasy mini columns (Qiagen) with on-column DNase I-treatment (RNase-free DNase I, Qiagen), as previously described^49^. Yield and purity were assayed with a NanoDrop (Thermo Scientific), and only high-quality samples were used for RT-(q)PCR and sequencing.

### Reverse transcription polymerase chain reaction

To assay transcript splicing as well as confirm E7a exclusion in the novel ClC-1^∆E7a^ line, standard RT-PCR protocols with primers located in exons flanking alternatively spliced regions were utilized. Briefly, reverse transcription to create cDNA libraries from each muscle total RNA sample was completed using *Superscript II* (Invitrogen) primed with oligo(dT)_15_ (Promega) using the standard manufacturer’s protocol with 1ug total RNA reverse transcribed per 20 μL reaction. cDNA used for long-read sequencing (PacBio) were treated with RNase H following reverse transcription, discussed further in long-read sequencing methods. For targeted assessment of alternative splicing, PCR products were amplified with Phusion polymerase (NEB) with primers in exons flank alternative spliced regions (Supplementary Table 1a) and visualized using standard gel electrophoresis with 2% agarose gels stained with ethidium bromide (EtBr). Gels were imaged using either a GelDoc or ChemiDoc gel imaging system (BioRad) equipped with a UV tray. Images were processed with Image Lab (BioRad) and band densitometry was determined with ImageJ (NIH). For comparing cassette exon and retained intron splicing, band intensities were normalized to the adult isoform splice isoform to account for differences in EtBr intercalation for different length amplicons. Percent Spliced In (PSI) values were calculated for all alternative splicing events assayed per the following equation:

1 $${{\rm{PSI}}}=\frac{{{\rm{inclusion}}}\; {{\rm{band}}}\; {{\rm{intensity}}}}{{{\rm{inclusion}}}\; {{\rm{band}}}\; {{\rm{intensity}}}+{{\rm{exclusion}}}\; {{\rm{band}}}\; {{\rm{intensity}}}}$$

To determine relative steady-state and pre-mRNA abundance of *Clcn*1, probe-based quantitative RT-PCR (RT-qPCR) was used with primer/probe sets either pre-designed to target exon-exon borders for assaying *Clcn*1 steady-state mRNA (IDT) or adapted to target intron-exon borders for assaying pre-mRNA levels. A pre-designed primer/probe set (IDT) for the TATA box binding protein (*Tbp*) was used as the reference/housekeeping gene for all reactions. All primer/probes are listed in Supplementary Table 1a with efficiencies greater than 90% (Supplementary Fig. 1). The Luna Universal Probe One-step RT-qPCR kit was used with 100 ng total RNA input from each sample (isolation as described above) in 20 μL reactions run with the Quantstudio 3 (Applied Biosystems, Thermo Fisher Scientific). The 2^-ΔΔ*CT*^ method was used to determine relative expression^79^. For all RT-PCR experiments, at least 3 biological replicates were used for splicing analysis. All RT-qPCR experiments were completed with at least 3 biological replicates per genotype/timepoint in technical triplicate. Samples were standardized to the mean P18 WT cycle threshold (Ct) for samples on each plate.

### Long-read whole-Clcn1 sequencing library preparation

To analyze transcript isoform utilization and splicing interactions at multiple locations in *Clcn*1, we used long-read whole-transcript sequencing with the Pacific Biosciences Single Molecule Real Time (SMRT) system (Supplementary Fig. 3a). This allowed for the generation of thousands of contiguous “whole transcript” reads from exon 1 to exon 23 of *Clcn*1 transcripts. Sequencing libraries were generated based on the PacBio protocol for multiplexing with barcoded universal primers (Pacific Biosciences). To start, RNA from quadriceps tissue from postnatal day 7 and 18 mice (3 biological replicates per genotype/timepoint) were reverse transcribed as discussed earlier. Two-microliters of each cDNA library was then used for target specific amplification (Phusion high-fidelity polymerase; NEB) of *Clcn*1 transcripts with primers designed to bind within exon 1 and exon 23 with overhanging sequences and 5’AmMC6 blocking (Supplementary Table 1a). Samples run in parallel were amplified for 30 cycles and visualized on a 1% agarose gel (EtBr staining) for verification of amplicon identity and specificity. An annealing temperature of 63 °C and 3% DMSO was used for first-step amplification. Samples were amplified for 20 cycles to progress in library preparation. These products were purified with AMPure XP beads (Beckman Coulter) at a volumetric ratio of 0.6 beads: 1 PCR product to select for the ~3 kb expected product. Next, 1.5 μL of purified product was used for barcoding PCRs with Pacbio Universal Barcoding Primers (Pacific Biosciences). Barcoded products were then purified again with AMPure XP beads, quantified with Qubit high-sensitivity DNA assays (Invitrogen), and then equimolar pooled to enter the PacBio DNA repair and adapter ligation protocol (SMRTbell Express Template Prep Kit 2.0, Pacific Biosciences). The pooled library was diluted and submitted for sequencing on the PacBio Sequel system at the Molecular Biology and Genomics Core (Washington State University, Pullman, WA).

### Long-read whole-Clcn1 sequencing analysis

Initial processing of circular-consensus sequences (ccs), quality control, and demultiplexing was completed with the SMRT Link software package (Pacific Biosciences). Q20 reads were then processed with the pbBioconda (Pacific Biosciences) distribution using the lima package to remove overhangs and Isoseq to refine reads. Reads then entered the FLAIR analysis pipeline to align reads to chromosome 6 (mm39), define transcript isoforms present, and quantify isoform utilization^29^. The Refseq annotation of mm39 was used for determining known isoforms (UCSC distribution). Next, isoform utilization was quantified for each sample. A total of 275,185 barcoded Q20 reads were generated, with an average of 8227 ± 4709 reads per sample (mean ± SD) with a median quality of Q38. To compare differential utilization of the various *Clcn*1 isoforms in the different sample groups DESeq2 on R was utilized for isoforms with greater than 100 reads mapped across all samples^80^.

### Whole-cell voltage-clamp recordings of ClC-1 currents

To assay functional expression of ClC-1 channels in these mice, whole-cell patch-clamp experiments on isolated *flexor digitorum brevis* (FDB) muscle fibers were completed with solutions designed to differentiate ClC-1 chloride currents. Experiments were completed as previously outlined^14^. Briefly, mice were euthanized by cervical dislocation and decapitation, the FDB was excised from both hindlimbs, digested with 2 mg/mL collagenase A (Roche) dissolved in standard Ringer’s solution for 45–60 min, and then dissociated by mechanical agitation with fire-polished pasture pipettes of decreasing bore diameter. Isolated fibers were plated on 35 mm cell-culture dishes (Falcon) and stored at room temperature in Ringer’s solution until used within 8 h of dissociation. Prior to experiments, Ringer’s solution was exchanged for external recording solution (in mM): 145 TEA-Cl, 10 CaCl_2_, 10 HEPES, 0.25 CdCl_2_, 0.003 nifedipine (pH 7.4 w/ TEA-OH). Then, whole-cell patch-clamp experiments were completed with low-resistance thin-walled pipettes (approximately 0.5–1.0 MOhms) were filled with (in mM): 130 Cs-aspartate, 10 CsCl, 5 MgCl_2_, 10 Cs_2_-EGTA, and 10 HEPES (pH 7.4 w/ CsOH). Solutions were designed to isolate Cl^-^ currents (TEA/Cs to block K^+^ currents; no Na^+^ present; and nifedipine and Cd^2+^ to block Ca^2+^ currents) with a reversal potential for Cl- (V_Cl-_) of approximately −53 mV. Recordings were collected with an Axopatch 200B (Molecular Devices) amplifier with voltage protocols and data acquisition controlled by pCLAMP 11 software (Molecular Devices). After formation of a gigaseal, whole-cell configuration was achieved, and fibers were held at −50 mV for greater than 5-min for equilibration. Series resistance was compensated (>90%), and currents were collected in response to voltage protocols featuring a pre-pulse to +60 mV (250 ms) for maximal activation of ClC-1 channels followed by a family of voltage-steps from −140 mV to +60 mV (∆10 mV; 500 ms; 0.1 Hz) to produce current-voltage (IV)-curves and tail currents for calculating relative open probability. To isolate ClC-1 currents, the external solution was then exchanged to also include 500 μM 9-Anthracenecarboxylic acid (9-AC) to block ClC-1. Currents from the same protocol were then collected, representing all non-9-AC-sensitive currents (e.g., leak, capacitive, and non-ClC-1 Cl^-^ currents). Offline subtraction of traces collected before and after the addition of 9-AC yielded just 9-AC sensitive currents, corresponding to ClC-1 currents. To standardize across fiber sizes, a capacitive transient will be recorded from a 10 mV depolarization from holding and integrated to calculate capacitance. Peak instantaneous and steady-state currents will then be divided by capacitance to yield current-densities (CD; pA/pF). Linear properties, including capacitance, access resistance, and membrane resistance were tracked with the Clampex software (Molecular Devices) throughout the collection of recordings. Preliminary analysis was completed with Clampfit software (Molecular Devices) with final analysis and plotting completed with Microsoft excel and GraphPad Prism 10/11 (Dotmatics). Representative traces were blanked to remove artifacts for presentation. Instantaneous currents were measured from voltage-step recordings immediately after the transition from holding at +60 mV, whereas steady-state current was measured at the end of the 500 ms voltage step. Instantaneous currents plotted versus voltage was fit with a modified Boltzmann equation for current at a specific membrane potential *I*(V_m_):

2 $$I\left({V}_{m}\right)=\frac{{I}_{\max }-{I}_{o}}{1+\exp \left({V}_{1/2}-{V}_{m}\right)/{k}_{v}}+{I}_{o}$$

*V*_*m*_ is membrane potential, *V*_1/2_ voltage of half-maximal activation, *k*_*v*_ is slope factor, *I*_*max*_ maximal current, and *I*_o_ is a constant offset current. Relative open probabilities based on normalized tail currents were fit to the equation of relative *P*_o_ at a given membrane potential: P_o,rel_(*V*_*m*_):

3 $${P}_{o,{rel}}\left({V}_{m}\right)=\frac{I\left({V}_{m}\right)}{{I}_{\max }}=\frac{1-{P}_{\min }}{1+\exp \left({V}_{1/2}-{V}_{m}\right)/{k}_{v}}+{P}_{\min }$$

*V*_*m*_ is membrane potential, *I*_max_ is the absolute maximal tail current, *V*_1/2_ voltage of half-maximal activation, *P*_min_ is the minimal value of *P*_o_ and *k*_*v*_ is slope factor.

### Rotarod

To assay the presence of myotonia and general muscle function rotarod experiments with a linear acceleration protocol were completed, as previously described^38^. Briefly, on day-1 of experiments, mice 10-to-12 weeks-postnatal were first acclimated to the rotarod (Columbus Instruments) spinning at 4 rotations-per-minute (rpm) for three 1-min trials separated by at least 15 min. Mice were then subjected to four-trials of linear-acceleration protocols starting at 4 rpm and accelerating to 40 rpm over 5-min at which time the rotarod continues to spin at a constant 40 rpm for an additional 5-min. Time-to-fall (seconds) and rotation speed (rpm) at which the mouse fell was recorded for each trial. At least 15-min of rest was provided between trials. The mice were then trained for another 2-days with 4-trials of linear acceleration protocol (3-days training total). Formal data collection was completed on the fourth day with 4-trials of linear acceleration protocol. The average of the 4-trials for each mouse was used for analysis.

### In vivo muscle-contraction with paired electromyography (EMG)

To assay in vivo force production, myotonia, and underlying electrical activity, a standard in vivo force contraction assay of murine plantarflexion was used with the addition of EMG recordings^81^. Mice were anesthetized with inhaled isoflurane, hair on the hind-limb assayed was shaved, and the mouse was placed on a heated (37 °C) platform with the knee fixed to immobile posts. Next, the footpad was securely attached to a footplate connected to a force-transducer to measure plantarflexion force (Aurora). Two stimulating electrodes were then placed on the medial aspect of the upper hindlimb to stimulate the tibial nerve and produce contraction of the posterior compartment of the lower hindlimb (primarily the gastrocnemius). Stimulating electrode placement and current amplitude were optimized to produce the peak force in response to single 1.25 millisecond (ms) test pulses. Next, a protocol run was delivered that featured 3 twitch contractions (0.2 ms) and a single tetanus (150 Hz for 500 ms). Following the acquisition of these recordings, monopolar EMG electrodes (iWorx Systems) were placed subcutaneously as close to the gastrocnemius muscle belly as possible at proximal and distal locations for the collection of EMG data; electrical activity in response to the same contraction protocol was collected secondary to the initial force recordings, as the presence of additional EMG electrodes suspended in the distal hindlimb provided artifacts in the force recordings. Additionally, a ground electrode was placed subcutaneously in the abdominal region. Data were collected and amplified with an IX-BIO 4 isolated biopotential recorder, then acquired with an IX-RA-5x attached to a workstation running the LabScribe software for data collection (iWorx Systems). The same protocol of three twitches followed by a single tetanus was delivered by the tibial nerve stimulating electrodes, and electrical activity was recorded with the LabScribe software (iWorx Systems). At least 5 min rest was provided between tetanus stimulations to prevent warm-up. Force recordings from the initial tetanus were measured for peak force calculations (normalized to mouse bodyweight) and myotonia (impulse of normalized force trace; time integral of normalized force). EMG signals were aligned based on the initial takeoff of the voltage signal in response to the stimulus artifact. To summarize the overall electrical activity of the muscle a linear envelope was calculated by rectifying and applying a second-order low-pass Butterworth filter with MATLAB (MathWorks)^82^. Fast Fourier Transform was utilized to evaluate amplitude/power spectra.

### Ex vivo muscle-contraction

Force measurements were collected from the soleus excised from 4 to 6-month postnatal mice. Established protocols were used for assaying muscle isometric force generation, frequency dependence, and myotonia, as previously described^9,83^. First, mice were anesthetized with inhaled isoflurane (~2%). *Soleus* muscles were carefully dissected and excised with suture loops tied to both proximal and distal tendons for later attachment to the immobile post and force transducer, respectively. Muscles were then submerged in a bath between two platinum stimulating electrodes in oxygenated (95% O_2_ and 5% CO_2_) and warmed Ringer’s solution. Ringer’s solution contained (in mM): 1.2 NaH_2_PO_4_, 1 MgSO_4_, 4.83 KCl, 137 NaCl, 2 CaCl_2_, 10 glucose, 24 NaHCO_3_ at pH 7.4. Force was measured with an Aurora Scientific 1200A ex vivo system equipped with an 809B muscle testing system with a 300C-LR force transducer and a 701C stimulator (Aurora Scientific). Muscles were equilibrated in the bath for at least 10 min prior to recordings. Optimal length (*L*_o_) of the muscle and stimulation current was determined for each muscle. The first stimulation protocol was for tetanic contractile properties (peak force and myotonia); three twitches (0.2 ms stimulation; 20 s between stimulations) and three tetani (500 ms of 0.2 ms stimuli delivered at 150 Hz) were recorded. Next, frequency dependence of force production was assayed with a standard protocol of increasing stimulation frequencies, from twitch to 200 Hz tetanus. All force recordings reported were normalized to the cross-sectional area (CSA) through measurement of muscle length and weight as well as the use of the appropriate pyknotic vector for each muscle and thus reported as specific force (mN/mm^2^). For frequency dependence, force was normalized to the peak force generated by the muscle across all the stimuli; therefore, reported as normalized force (arbitrary unit, AU). Similarly, for assaying myotonia through impulse calculation, peak force generated during the tetanic stimuli was normalized to 1 AU. The time integral of the force trace following the tetanic stimuli was then calculated to provide the area under curve (impulse) of myotonia.

### Histology – conventional staining and immunofluorescence

For analysis of skeletal muscle histopathologic hallmarks of DM1, standard hematoxylin and eosin (H&E) was used for assessing central nucleation. Muscles were mounted with tragacanth gum, frozen in 2-methylbutane submerged in liquid nitrogen, and stored at −80 °C until further processing. Transverse slices (10 μm) were obtained with a cryostat (Thermo Scientific) and transferred to slides for staining such that each slide contained at least one section of each genotype assayed. Slides were stored at −80 °C prior to staining. For determination of fiber-type composition, myosin heavy chain (MyHC) immunofluorescence (IF) was used for visualization of type I, IIa, IIb, and IIx fibers. Slices were first permeabilized for 10-min with PBST (PBS with 0.2% Triton X-100) and then blocked at room temperature for 30-min in 10% normal goat serum (NGS) (Gibco, Thermo Fisher Scientific) in PBS. Additional blocking with 0.1 mg/mL AffiniPure Fab fragment anti–mouse IgG (3%; H&L) (Jackson Immunoresearch) in PBS with 2% normal goat serum for 60-min (room temperature). Slides were then washed (3 times with fresh PBS for 10-min at room temperature) and incubated with primary antibodies (Supplementary Table 1b) overnight (14–18 h) at 4 °C, primary antibodies against myosin heavy chain isoforms were obtained from the Developmental Studies Hybridoma Bank (DSHB) created by the NICHD of the NIH and maintained at The University of Iowa, Department of Biology (Iowa City, IA). The next day, slides were washed again with PBS (3 times for 10-min at room temperature). Then, secondary antibodies were applied for 1-h at room temperature. Slides were then washed with PBS (3 times for 10-min at room temperature) and finally mounted with Fluoromount-G (Invitrogen). No primary antibody control slides were completed for all samples with all IF paradigms and were used for establishing exposure levels when imaging each slide. Imaging was performed with a Keyence BZ-X810 All-in-One Fluorescence Microscope. Magnification and scale are noted for all included images. Percent fibers with central nuclei were manually calculated as the number of fibers with 1+ centrally located nuclei divided by the total number of intact fibers in a complete H&E-stained transverse section (image collected at 10x and stitched with Keyence analysis software). Individuals performing quantification were blinded to genotype when possible. Fiber morphometry and typing was performed with Myosoft^84^. For presentation of minimal Feret’s diameter and CSA, values for each muscle sample were filtered for outliers to remove rare errors of the automated analysis software by the ROUT method (*Q* = 1%) with Graphpad Prism 10. For presentation of representative images, ImageJ (NIH) with the plugin *Quick Figures* was utilized^85^.

### RNA sequencing – data collection, preliminary data processing, and expression analysis

RNA was isolated from quadriceps muscle from 4–6-month-old WT (2:2), ClC-1^∆E7a/∆E7a^ (2:2), Mbnl1^−/−^ (3:1), and Mbnl1^−/−^; ClC-1^∆E7a/∆E7a^ (1:3) mice (male to female) as described above (RNA isolation). Libraries were prepared with Illumina Stranded Total RNA Prep Ligation with Ribo-Zero Plus and sequenced on an Illumina NovaSeq6000. Raw reads generated from the Illumina base calls were demultiplexed using bcl2fastq (version 2.20.0). Quality filtering and adapter removal were performed using FastP (version 0.23.1)^86^. Processed/cleaned reads were then mapped to the GRCm39/gencode M31 reference using STAR_2.7.9a^87,88^. Gene-level read quantification was derived using the subread-2.0.1 package (featureCounts) with a GTF annotation file (GRCm39/gencode M31)^89^. Differential expression analysis was performed using DESeq2 with a minimum threshold of 50 aggregate reads per gene and contrasts completed between each experimental and control group^80^. Improved expression in Mbnl1^−/−^; ClC-1^∆E7a/∆E7a^ samples was defined as transcripts with significant expression changes in Mbnl1^−/−^ compared to WT (∣log_2_FC∣ > 0.5, p-adj. <0.05) and with significant changes in expression towards WT when Mbnl1^−/−^; ClC-1^∆E7a/∆E7a^ were directly compared to Mbnl1^−/−^ samples (*p*-adj. < 0.05). PCA analysis was completed with pcaExplorer^90^. Heatmaps were generated with ComplexHeatmap^91^. Venn diagrams were created with Bioinformatics and Evolutionary Genomics web tool (https://bioinformatics.psb.ugent.be/webtools/Venn/).

### Short-read mRNA-sequencing splicing analysis

Isolated RNA was sequenced to a depth of ~60 million reads and mapped against the GRCm39 mouse genome using the STAR 2.7.0 aligner. The aligned reads were analyzed for splicing alterations using MAJIQ and ΔPSI-values were calculated in pair-wise comparisons for all conditions with of ΔPSI > 0.1 or <-0.1 and an FDR < 0.05 was set to be significantly changed. For visualization, MAJIQ modulizer results separated after event types (exon skipping, intron retention, alternative 3’ splice sites and 5’splice sites) were loaded into R 4.2. Partial rescues were defined as having more than a 0.15 delta-PSI shift in Mbnl1^−/−^; ClC-1^∆E7a/∆E7a^ towards control as compared to Mbnl1^−/−^ mice. Events were considered to display a complete rescue with ΔPSI > 0.1 or <-0.1 in Mbnl1^−/−^ vs WT mice and ΔPSI < 0.1 or >-0.1 (non-changing) in Mbnl1^−/−^; ClC-1^∆E7a/∆E7a^ vs WT at an FDR of < 0.05.

### Statistics

Statistical analysis and graphing were predominantly completed with GraphPad Prism 10/11. For RNA-seq and long-read PacBio sequencing, statistics with DESeq2 were completed with R (4.3.1). Some illustrations and schematics were generated with BioRender (Biorender.com). For comparisons of more than two groups with parametric data that features reasonable sphericity, standard one-way ANOVAs with secondary multiple comparisons with Tukey’s correction were completed. For comparisons with clear differences in variance between groups, Brown-Forsythe and Welch ANOVA tests with secondary multiple comparisons with Dunnett T3 correction were completed. For non-parametric comparisons, a Kruskal–Wallis test with Dunn’s multiple comparisons was completed. Comparison between genotypes across different timepoints was completed with two-way ANOVA with post-hoc Tukey’s multiple comparisons or Sidak multiple comparisons test when only selected comparisons were predetermined. Statistics for long-read sequencing and RNAseq analysis were completed as discussed in respective methods sections. For all comparisons shown, significance is indicated as follows unless otherwise specified: multiplicity adjusted *p*-value < 0.05*, <0.01**, <0.001***, and <0.0001****.

## Supplementary information

Supplementary Information

Transparent Peer Review file

## Source data

Source Data

## Data Availability

RNA sequencing data generated for this study have been deposited in Gene Expression Omnibus with the accession codes GSE311069 and GSE311013. All other data are presented in the manuscript and included in the supplementary information and Source data file. Source data are provided with this paper.

## Materials availability

Requests for further information and resources should be directed to and will be fulfilled by the lead contact, John Lueck (john_lueck@urmc.rochester.edu). Materials will be made available upon request.
