# Supplementary material for: Elimination of myotonia improves myopathy in a muscleblind-like knockout model of myotonic dystrophy
Source: Nat Commun. 2026 Jul 8;17:8429. doi: 10.1038/s41467-026-75243-x (PMC13478325; doi:10.1038/s41467-026-75243-x)
Supplement: Supplementary file 1 [file 41467_2026_75243_MOESM1_ESM.pdf]

Elimination of myotonia improves myopathy in a muscleblind-like knockout model of myotonic dystrophy

Supplementary Figures and Tables

Matthew T. Sipple[1,2], Sakura A. Hamazaki[3], Vanessa Todorow[4], Lily A. Cisco[2], Katherine M. Lupia[2], Christina S. Heil[5], Peter Meinke[6], Charles A. Thornton[5,7], John D. Lueck[2,5,7,*].

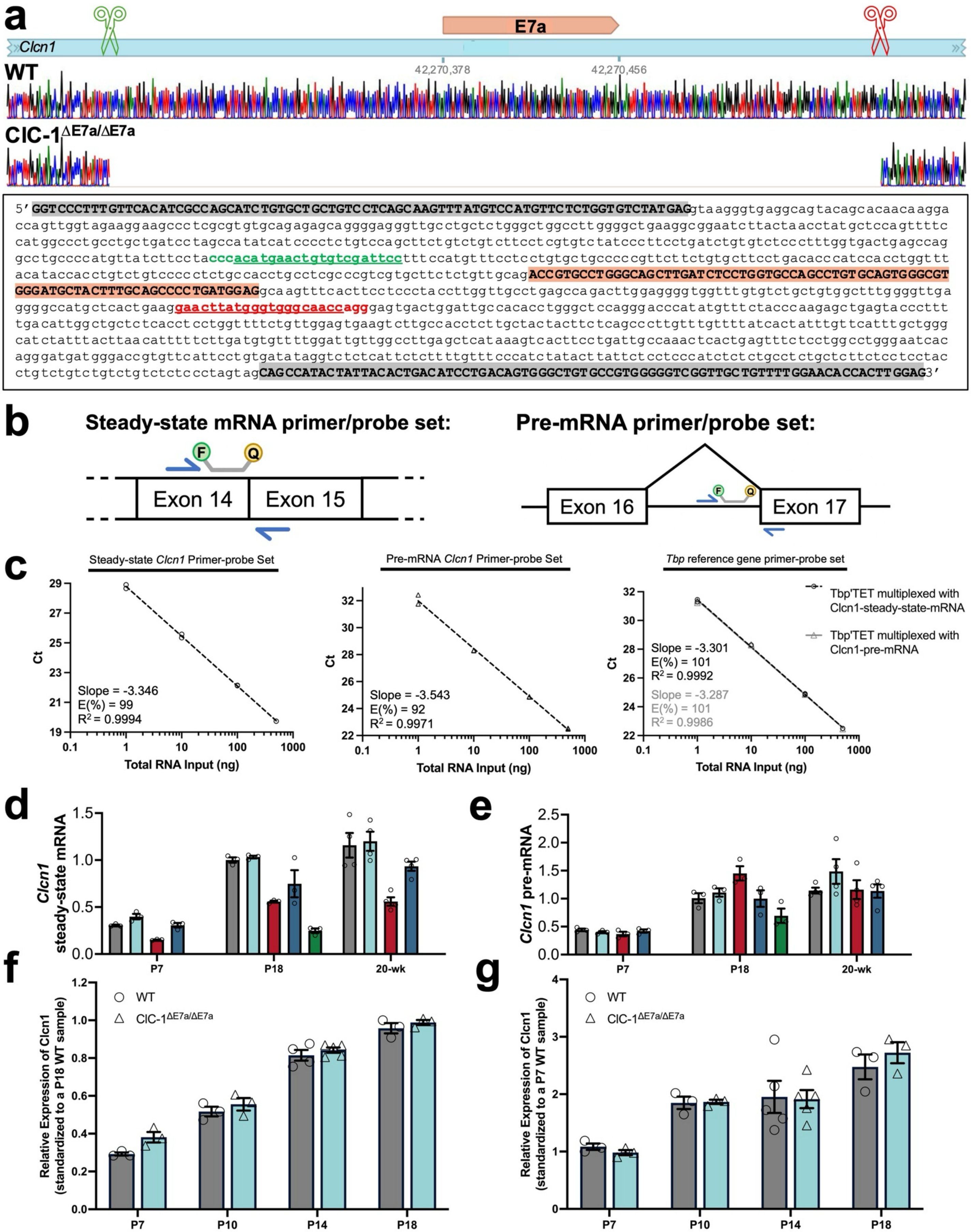
a
E7a
Clcn1
42,270,378
42,270,456
WT
ClC-1ΔE7a/ΔE7a
5′GGTCCCTTTGTTCACATCGCCAGCATCTGTGCTGCTGTCCTCAGCAAGTTTATGTCCATGTTCTCTGGTGTCTATGAGgtaagggtgaggcagtacagcacaacaagga
ccagttggtagaaggaagccctcgcgtgtgcagagagcaggggagggttgcctgctctgggctggccttggggctgaaggcggaatcttactaacctatgctccagttttc
catggccctgcctgctgatcctagccatatcatcccctctgtccagcttctgtctgtcttcctcgtgtctatcccttcctgatctgtgtctccctttggtgactgagccag
gcctgccccatgttatcttcctacccacatgaactgtgtcgattcctttccatgtttcctcctgtgctgccccgttcttctgtgcttcctgacacccatccacctggttt
acataccacctgtctgtcccctctgccacctgcctcgcccgtcgtgcttctctgttgcagACCGTGCCTGGGCAGCTTGATCTCCTGGTGCCAGCCTGTGCAGTGGGCGT
GGGATGCTACTTTGCAGCCCCTGATGGAGgcaagtttcacttcctccctaccttggttgcctgagccagacttggaggggtggtttgtgtctgctgtggctttggggttga
ggggccatgctcactgaaggaacttatgggtgggcaaccagggagtgactggattgccacacctgggctccagggacccatatgtttctacccaagagctgagtacccttt
tgacattggctgctctcacctcctggtttctgttggagtgaagtcttgccacctcttgctactacttctcagcccttgtttgttttatcactatttgttcatttgctggg
catctatttacttaacatttttcttgatgtgtttggattgttggccttgagctcataaagtcacttcctgattgccaaactcactgagtttctcctggcctgggaatcac
agggatgatgggaccgtgttcattcctgtgatataggtctctcattctctttgtttcccatctatacttattctcctcccatctctctgcctctgctcttctcctcctac
ctgtctgtctgtctgtctctcccctagtagCAGCCATACTATTACACTGACATCCTGACAGTGGGCTGTGCCGTGGGGGTCGGTTGCTGTTTTGGAACACCACTTGGAG3′
b
Steady-state mRNA primer/probe set:
F
Q
Exon 14
Exon 15
Pre-mRNA primer/probe set:
Exon 16
Exon 17
c
Steady-state Clcn1 Primer-probe Set
Slope = -3.346
E(%) = 99
R2 = 0.9994
Ct
Total RNA Input (ng)
Pre-mRNA Clcn1 Primer-probe Set
Slope = -3.543
E(%) = 92
R2 = 0.9971
Tbp reference gene primer-probe set
Tbp'TET multiplexed with Clcn1-steady-state-mRNA
Tbp'TET multiplexed with Clcn1-pre-mRNA
Slope = -3.301
E(%) = 101
R2 = 0.9992
Slope = -3.287
E(%) = 101
R2 = 0.9986
d
Clcn1 steady-state mRNA
P7
P18
20-wk
e
Clcn1 pre-mRNA
P7
P18
20-wk
f
WT
ClC-1ΔE7a/ΔE7a
Relative Expression of Clcn1 (standardized to a P18 WT sample)
P7
P10
P14
P18
g
WT
ClC-1ΔE7a/ΔE7a
Relative Expression of Clcn1 (standardized to a P7 WT sample)
P7
P10
P14
P18

SF 1

Supplementary Figure 1. Generation of Clcn1 exon 7a exclusion mouse and RT-qPCR assessment of *Clcn1.* (a) Verification of genomic excision of exon 7a sequence (and flanking intronic regions) from *Clcn1.* Upper panel shows electropherogram of Sanger sequencing of targeted amplification of the *Clcn1* gene from genomic DNA isolated from WT and ClC-1$^{\Delta E7a/\ \Delta E7a}$ samples, aligned to the *Clcn1* sequence above with CRISPR cut sites indicated by scissors and the exon 7a sequence annotated. Bottom panel represents the *Clcn1* genomic sequence flanking exon 7a (highlighted in red) with exons 6 and 7 shaded in gray and CRISPR cut sequences in green and red text. Electropherogram and schematic generated with Benchling.com. (b) Schematics of the design of RT-qPCR primer-probe sets for assessing steady-state mature *Clcn1* mRNA (IDT) and *Clcn1* pre-mRNA. (c) Standard curves to assess primer-probe efficiencies estimated by dilution series on total RNA isolated from P14 WT mouse (completed in technical triplicate). (d) Relative steady-state *Clcn1* and (e) *Clcn1* pre-mRNA used for calculation of steady-state to pre-mRNA ratio in Fig. 1d. (f) Relative steady-state *Clcn1* and (g) *Clcn1* pre-mRNA across early postnatal development. *Tbp* as housekeeping. Normalized to P18 WT average or P7 WT average. P7 and P18 data replotted from above. Dots represent biological replicates. Completed in technical triplicate. Data shown mean ± SEM.

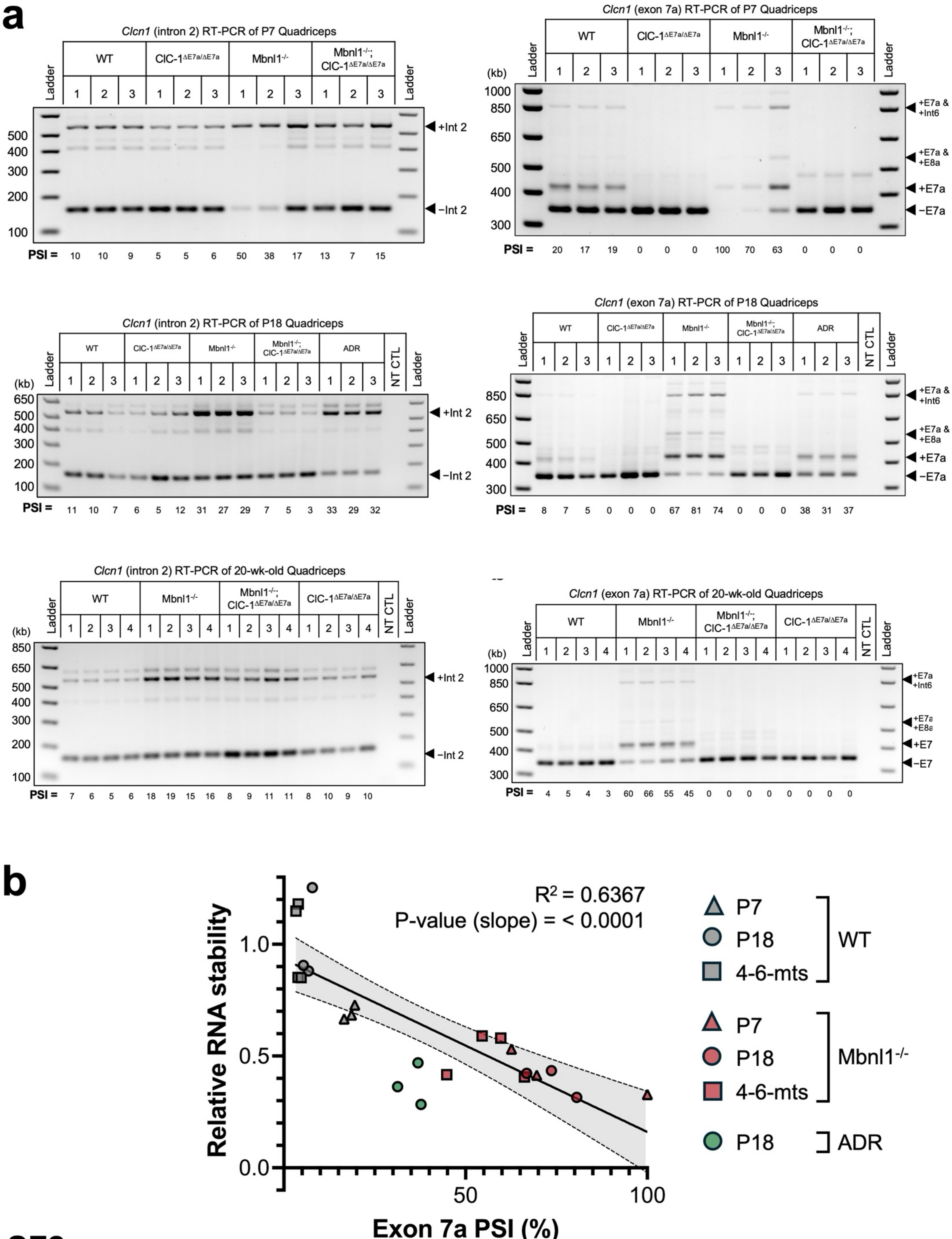
a
Clcn1 (intron 2) RT-PCR of P7 Quadriceps
Ladder
WT
ClC-1ΔE7a/ΔE7a
Mbnl1-/-
Mbnl1-/-; ClC-1ΔE7a/ΔE7a
1 2 3 1 2 3 1 2 3 1 2 3
500
400
300
200
100
+Int 2
−Int 2
PSI = 10 10 9 5 5 6 50 38 17 13 7 15
Clcn1 (exon 7a) RT-PCR of P7 Quadriceps
(kb)
1000
850
650
500
400
300
+E7a & +Int6
+E7a & +E8a
+E7a
−E7a
PSI = 20 17 19 0 0 0 100 70 63 0 0 0
Clcn1 (intron 2) RT-PCR of P18 Quadriceps
WT
ClC-1ΔE7a/ΔE7a
Mbnl1-/-
Mbnl1-/-; ClC-1ΔE7a/ΔE7a
ADR
NT CTL
(kb)
650
500
400
300
200
100
+Int 2
−Int 2
PSI = 11 10 7 6 5 12 31 27 29 7 5 3 33 29 32
Clcn1 (exon 7a) RT-PCR of P18 Quadriceps
850
650
500
400
300
+E7a & +Int6
+E7a & +E8a
+E7a
−E7a
PSI = 8 7 5 0 0 0 67 81 74 0 0 0 38 31 37
Clcn1 (intron 2) RT-PCR of 20-wk-old Quadriceps
WT
Mbnl1-/-
Mbnl1-/-; ClC-1ΔE7a/ΔE7a
ClC-1ΔE7a/ΔE7a
NT CTL
1 2 3 4 1 2 3 4 1 2 3 4 1 2 3 4
(kb)
850
650
500
400
300
200
100
+Int 2
−Int 2
PSI = 7 6 5 6 18 19 15 16 8 9 11 11 8 10 9 10
Clcn1 (exon 7a) RT-PCR of 20-wk-old Quadriceps
(kb)
1000
850
650
500
400
300
+E7a +Int6
+E7a +E8a
+E7
−E7
PSI = 4 5 4 3 60 66 55 45 0 0 0 0 0 0 0 0
b
R^2 = 0.6367
P-value (slope) = < 0.0001
Relative RNA stability
0.0
0.5
1.0
50
100
Exon 7a PSI (%)
P7
P18
4-6-mts
WT
P7
P18
4-6-mts
Mbnl1-/-
P18
ADR

SF2

Supplementary Figure 2. Representative RT-PCR gels and relationship between exon 7a splicing and relative RNA stability. (a) Representative agarose gels stained with EtBr for targeted RT-PCR splicing analysis of intron 2 retention and exon 7a inclusion for *Clcn1* with WT, ClC-1$^{\Delta E7a/\Delta E7a}$, Mbnl1$^{-/-}$, Mbnl1$^{-/-}$; ClC-1$^{\Delta E7a/\Delta E7a}$, and ADR samples as indicated at P7, P18, and 4-6-month time points. Lanes represent biological replicates. Ladder (1kb Plus ladder, Invitrogen) and no template control (NT CTL) shown. Gel for P18 exon 7a splicing is reproduced from Fig. 1b for completeness. (b) Linear regression analysis of samples plotted with exon 7a PSI against relative RNA stability, for all samples without forced exon 7a exclusion. Line represents simple linear regression with shaded area representing 95% CI.

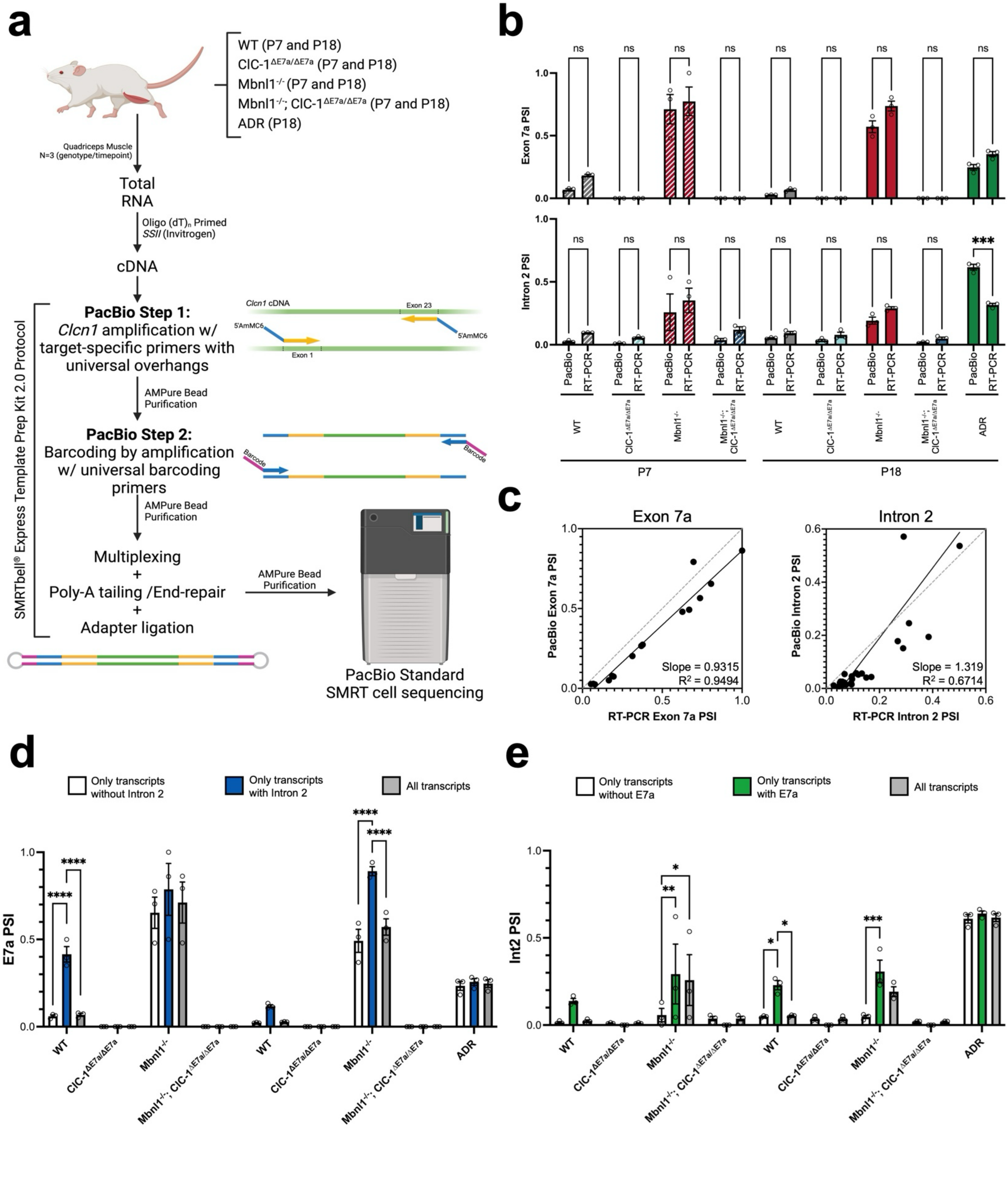
a
WT (P7 and P18)
ClC-1ΔE7a/ΔE7a (P7 and P18)
Mbnl1-/- (P7 and P18)
Mbnl1-/-; ClC-1ΔE7a/ΔE7a (P7 and P18)
ADR (P18)
Quadriceps Muscle
N=3 (genotype/timepoint)
Total RNA
Oligo (dT)n Primed
SSII (Invitrogen)
cDNA
SMRTbell® Express Template Prep Kit 2.0 Protocol
PacBio Step 1:
Clcn1 amplification w/ target-specific primers with universal overhangs
Clcn1 cDNA
Exon 23
5'AmMC6
5'AmMC6
Exon 1
AMPure Bead Purification
PacBio Step 2:
Barcoding by amplification w/ universal barcoding primers
Barcode
Barcode
AMPure Bead Purification
Multiplexing
+
Poly-A tailing /End-repair
+
Adapter ligation
AMPure Bead Purification
PacBio Standard SMRT cell sequencing
b
Exon 7a PSI
Intron 2 PSI
ns
***
PacBio
RT-PCR
WT
ClC-1ΔE7a/ΔE7a
Mbnl1-/-
Mbnl1-/-; ClC-1ΔE7a/ΔE7a
ADR
P7
P18
c
Exon 7a
PacBio Exon 7a PSI
RT-PCR Exon 7a PSI
Slope = 0.9315
R2 = 0.9494
Intron 2
PacBio Intron 2 PSI
RT-PCR Intron 2 PSI
Slope = 1.319
R2 = 0.6714
d
Only transcripts without Intron 2
Only transcripts with Intron 2
All transcripts
E7a PSI
****
e
Only transcripts without E7a
Only transcripts with E7a
All transcripts
Int2 PSI
*
**
***

SF 3

Supplementary Figure 3 Long-read *Clcn1* sequencing and comparison to traditional RT-PCR splicing analysis. (a) Schematic of targeted whole-*Clcn1* transcript PacBio sequencing. Created in BioRender. Sipple, M. (2026) https://BioRender.com/j89e325. (b) Comparison between exon 7a and intron 2 PSI values derived from PacBio transcript utilization analysis and traditional RT-PCR with band densitometry. Comparisons with two-way ANOVA comparing genotype-age and splicing assay with selected Šidák corrected post-hoc comparisons shown with "ns" representing $p \geq 0.05$ and *** $p < 0.001$. (c) Linear regression analysis of samples plotted against PacBio and RT-PCR determined PSI values for exon 7a inclusion (left) and intron 2 retention (right). Simple linear regression shown by solid black line with slope and R-squared values as indicated. Dotted line represents a slope of 1 (y=x). (d) Combinatorial splicing analysis of full-length *Clcn1* transcripts displaying exon 7a PSI based on intron 2 status of the transcripts (only transcripts without intron 2, only transcripts with intron 2, or all transcripts). (e) Combinatorial splicing analysis of full-length *Clcn1* transcripts displaying intron 2 PSI based on exon 7a status of the transcripts (only transcripts without exon 7a, only transcripts with exon 7a, or all transcripts). Presented mean ± SEM with superimposed biological replicates at dots. Analysis completed with two-way ANOVA (genotype and splicing status) with intra-genotype comparisons between splicing status shown. Multiplicity adjusted p-value shown as * $p < 0.05$, ** $p < 0.01$, *** $p < 0.001$, **** $p < 0.0001$.

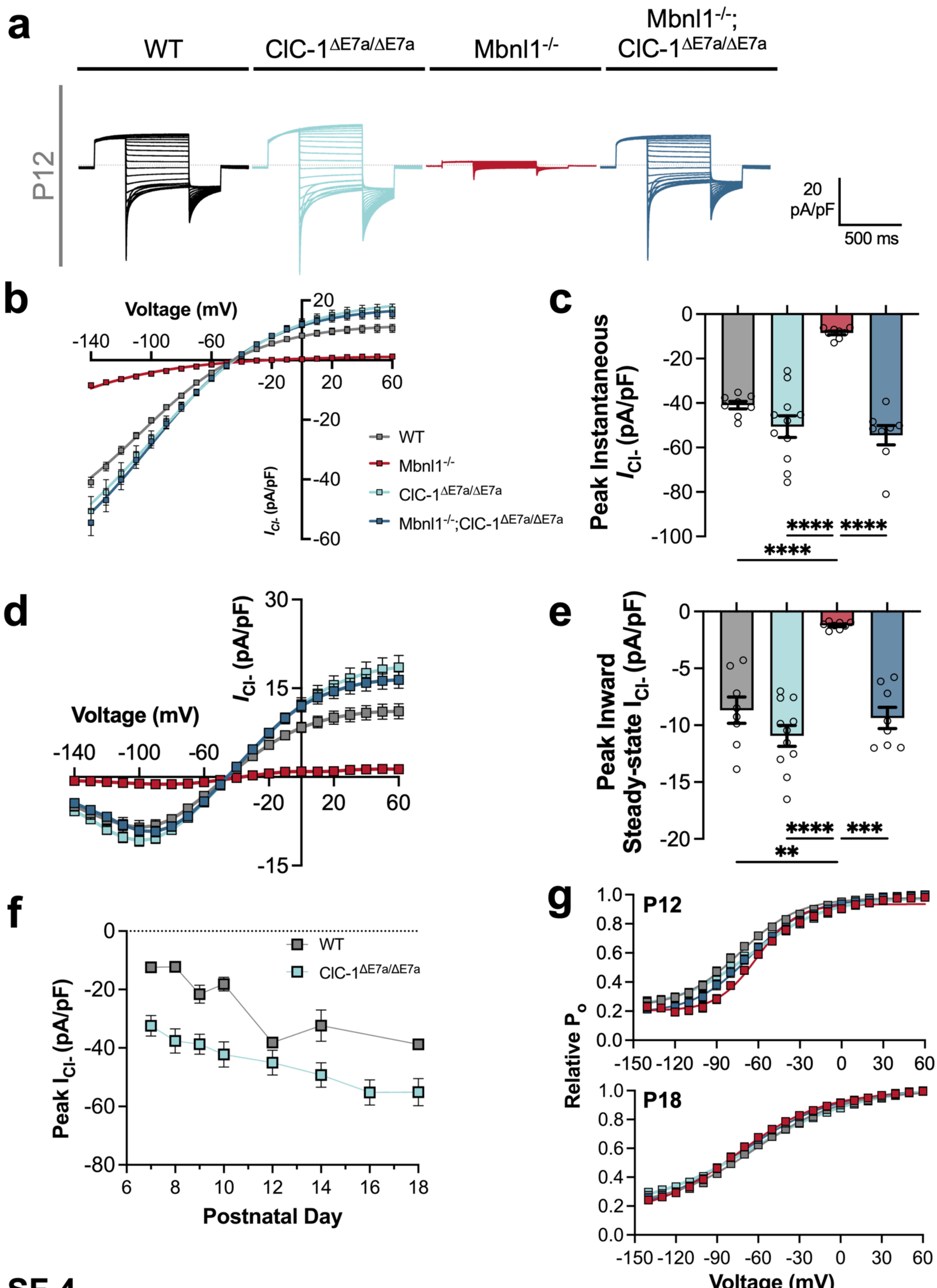
a
WT
ClC-1ΔE7a/ΔE7a
Mbnl1-/-
Mbnl1-/-; ClC-1ΔE7a/ΔE7a
P12
20 pA/pF
500 ms
b
Voltage (mV)
I Cl- (pA/pF)
WT
Mbnl1-/-
ClC-1ΔE7a/ΔE7a
Mbnl1-/-;ClC-1ΔE7a/ΔE7a
c
Peak Instantaneous I Cl- (pA/pF)
****
d
Voltage (mV)
I Cl- (pA/pF)
e
Peak Inward Steady-state I Cl- (pA/pF)
****
***
**
f
Peak I Cl- (pA/pF)
Postnatal Day
WT
ClC-1ΔE7a/ΔE7a
g
P12
P18
Relative Po
Voltage (mV)

SF 4

Supplementary Figure 4 Whole-cell patch-clamp of ClC-1 currents. (a) Representative traces of ClC-1 currents from WT, ClC-$1^{\Delta E7a/\Delta E7a}$, Mbnl$1^{-/-}$, and Mbnl$1^{-/-}$; ClC-$1^{\Delta E7a/\Delta E7a}$ samples collected at P12. Scale as shown. (b) Current density versus voltage for peak instantaneous inward current in response to family of voltage steps (-140 to 60 mV) with peak inward current for each P12 recording compared in (c) as a bar graph. (d) Average steady-state current versus voltage with max inward steady-state current compared in (e) as a bar graph. Comparisons with one-way ANOVA with post-hoc multiple comparisons. Multiplicity adjusted p-values shown as ** $p < 0.01$, *** $p < 0.001$, and **** $p < 0.0001$. (f) ClC-1 current densities across early post-natal development (P7 to P18) for WT and ClC-$1^{\Delta E7a/\Delta E7a}$ samples (n= 2+ mice with multiple fibers per mouse per timepoint per genotype). (g) Relative open probability versus voltage as determined by amplitude of tail currents for P12 and P18 samples fit with modified Boltzmann equation. Data shown mean ± SEM. Linear properties and values of Boltzmann fit shown in Supplementary table 2.

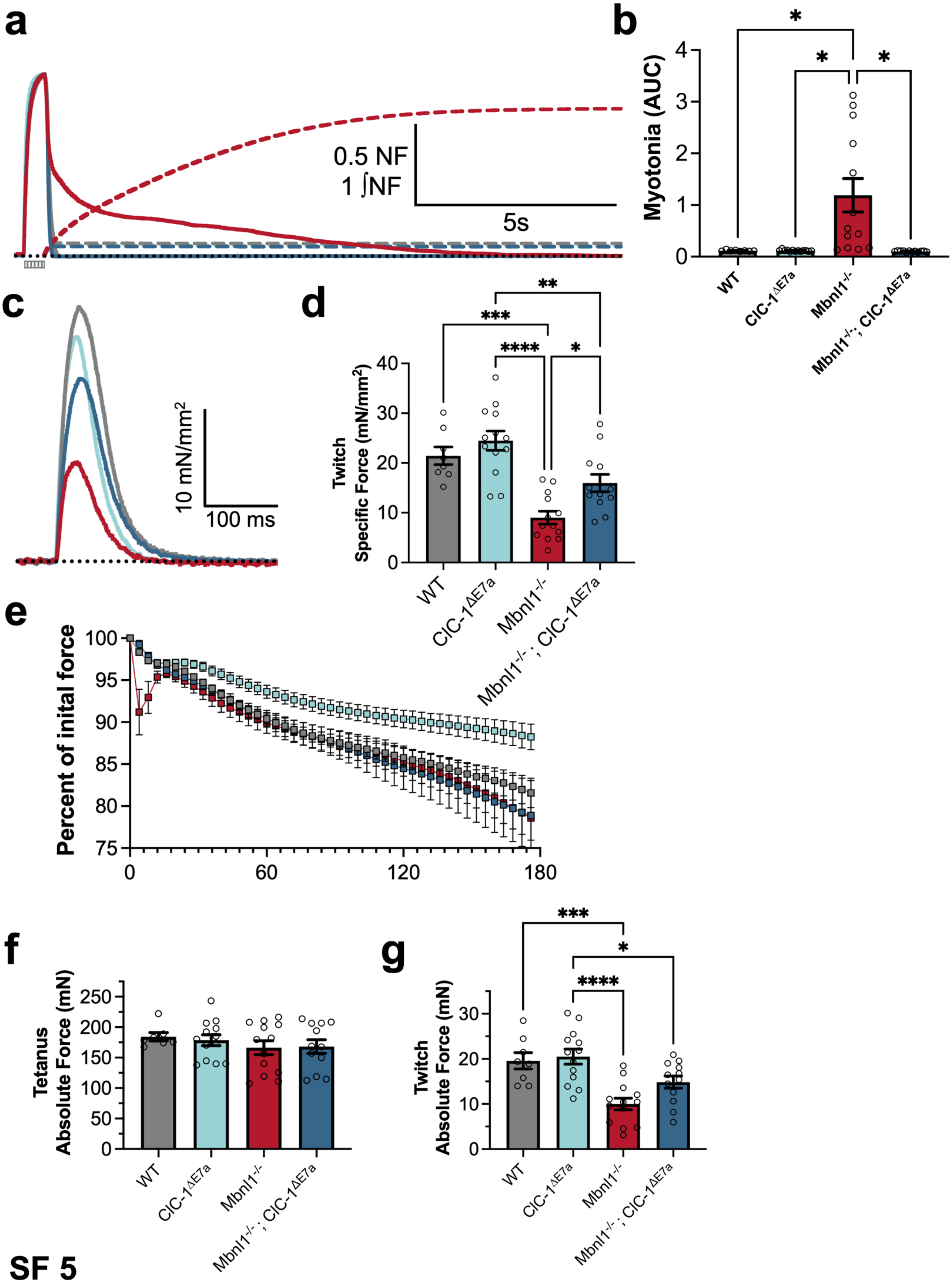

a
0.5 NF
1 ∫NF
5s
b
Myotonia (AUC)
WT
ClC-1ΔE7a
Mbnl1-/-
Mbnl1-/-; ClC-1ΔE7a
c
10 mN/mm2
100 ms
d
Twitch
Specific Force (mN/mm2)
e
Percent of inital force
f
Tetanus
Absolute Force (mN)
g
Twitch
Absolute Force (mN)

SF 5

Supplementary Figure 5 *Ex vivo* force-contraction from isolated solei muscles. (a) Representative normalized force traces in response to tetanic stimuli (500 ms, 150Hz) with area under the myotonic force represented by dotted lines. Scales as shown. (b) Myotonia (AUC) compared for samples (arbitrary units). Data presented as mean ± SEM with points representing biological replicates (individual Soleus muscles). (c) Representative twitch trace in response to a single 0.2 ms stimulus displayed as specific force versus time, scale as indicated on inset scale bars. Colors for each genotype as per bar graph shading above. (d) Peak twitch specific force across genotypes. Data presented as mean ± SEM with points representing biological replicates (individual Soleus muscles). (e) Peak specific force output in response to repetitive tetanic stimuli to assess transient weakness and fatigue. Protocol comprised of 45 consecutive tetani (500 ms, 100Hz) delivered every 4 seconds (duty cycle = 4s). Colors for each genotype as per bar graph shading above. Data presented as mean ± SEM. (f) Peak absolute force generated in response to tetanic stimulus (150 Hz, 500 ms). (g) Peak absolute force generated for twitch. Data presented as mean ± SEM with individual biological replicates shown. All statistical comparisons shown, represent one-way ANOVA with Tukey's multiple comparisons. Multiplicity adjusted p-values shown as * $p < 0.05$, ** $p < 0.01$, *** $p < 0.001$, and **** $p < 0.0001$.

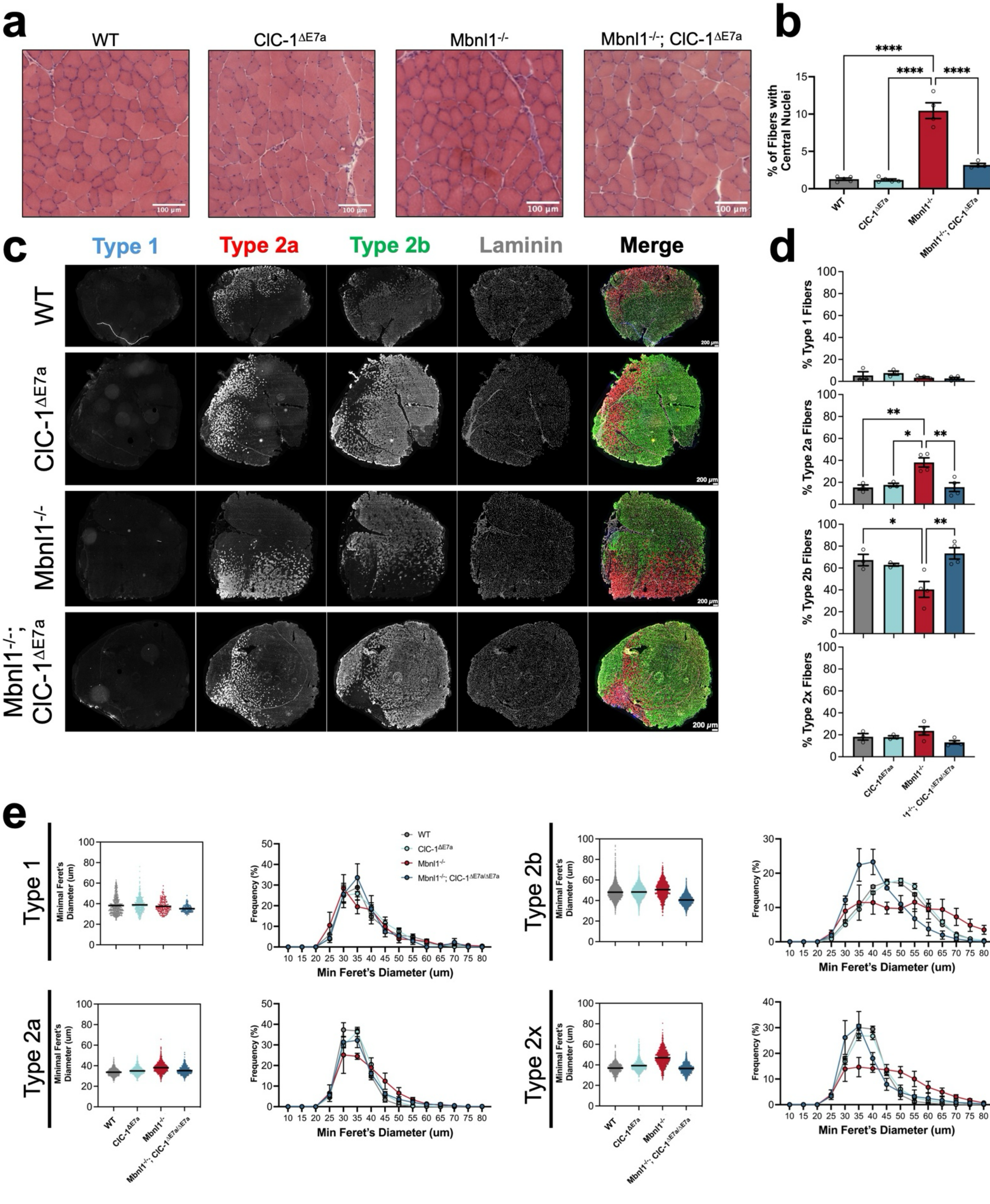

a
WT
ClC-1ΔE7a
Mbnl1-/-
Mbnl1-/-; ClC-1ΔE7a
100 µm
b
% of Fibers with Central Nuclei
****
c
Type 1
Type 2a
Type 2b
Laminin
Merge
200 µm
d
% Type 1 Fibers
% Type 2a Fibers
% Type 2b Fibers
% Type 2x Fibers
e
Type 1
Type 2a
Type 2b
Type 2x
Minimal Feret's Diameter (um)
Frequency (%)
Min Feret's Diameter (um)
WT
ClC-1ΔE7a
Mbnl1-/-
Mbnl1-/-; ClC-1ΔE7a/ΔE7a

SF 6

Supplementary Figure 6 Histological analysis of quadriceps muscle. (a) Representative images of quadriceps transverse sections stained with hematoxylin and eosin (H&E) from WT, ClC-1$^{\Delta E7a/\Delta E7a}$, Mbnl1$^{-/-}$, and Mbnl1$^{-/-}$; ClC-1$^{\Delta E7a/\Delta E7a}$ mice (4-6-month-old). Scale bars shown represent 100 micrometers. (b) Percent of fibers with central nuclei present on H&E stained quadriceps samples. (c) Fiber-typing by myosin heavy chain (MyHC) isoform was completed by immunofluorescence (IF), representative images shown, with each MyHC isoform channel and laminin counterstain shown in grayscale and with the merged image pseudo-colored (based on color of channel labels). (d) Percent of fibers staining for each of the respective MyHC isoforms. Comparisons with one-way ANOVAs with Tukey's multiple comparisons. Multiplicity adjusted p-values of significant comparisons presented as * $p<0.05$, ** $p<0.01$, *** $p<0.001$, and **** $p<0.0001$. (e) Fiber morphometry based on each fiber-type. Bar graph of aggregate fibers to the left and histogram to the right.

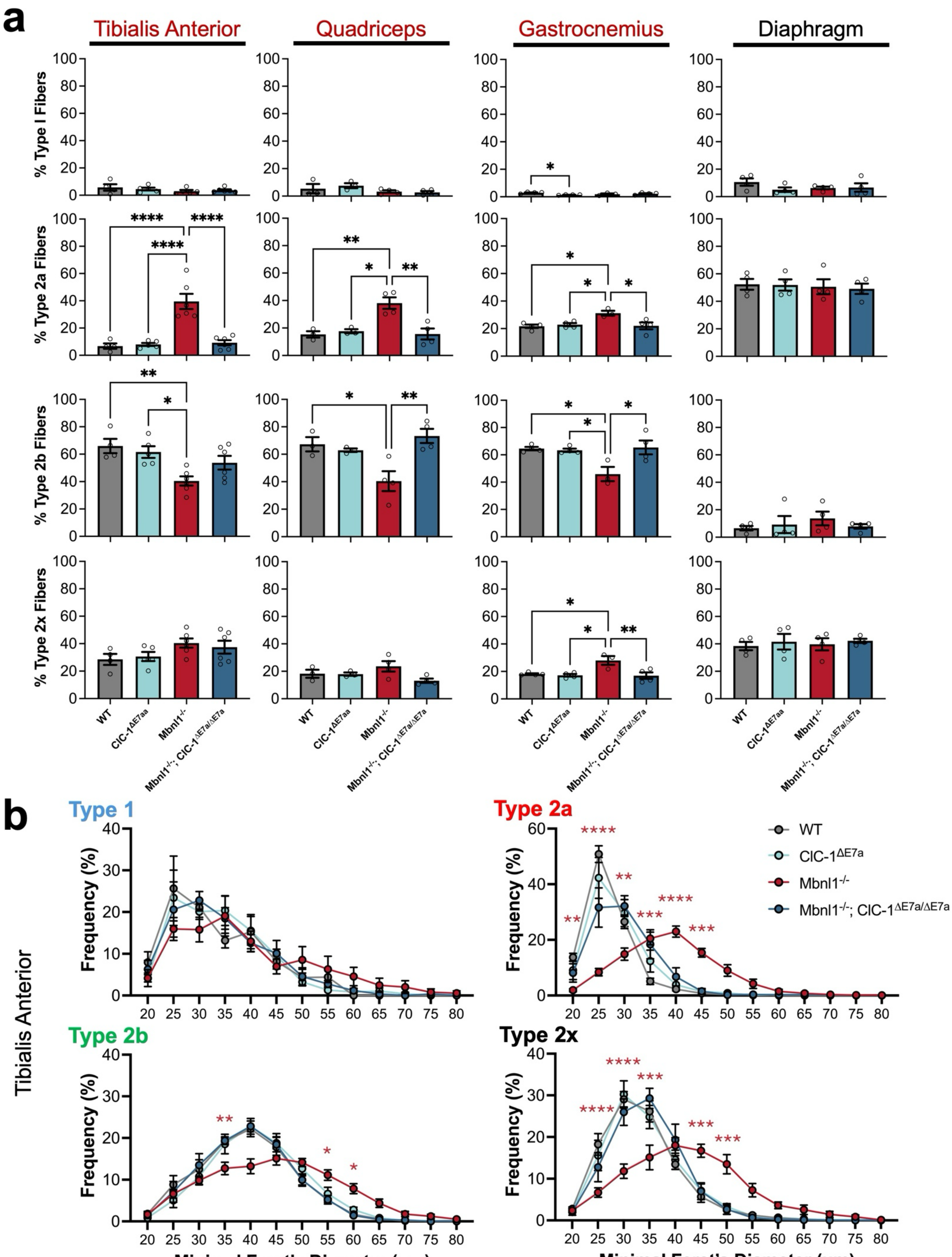
a
Tibialis Anterior
Quadriceps
Gastrocnemius
Diaphragm
% Type I Fibers
% Type 2a Fibers
% Type 2b Fibers
% Type 2x Fibers
WT
ClC-1ΔE7aa
Mbnl1-/-
Mbnl1-/-; ClC-1ΔE7a/ΔE7a
b
Tibialis Anterior
Type 1
Type 2a
Type 2b
Type 2x
Frequency (%)
Minimal Feret's Diameter (um)
WT
ClC-1ΔE7a
Mbnl1-/-
Mbnl1-/-; ClC-1ΔE7a/ΔE7a

SF 7

Supplementary Figure 7 Fiber type analysis across hindlimb and diaphragm muscles as well as TA fiber-type morphometry. (a) Fiber-type analysis by MyHC IF for transverse slices from tibialis anterior (transposed from Fig. 5a), quadriceps, gastrocnemius, and diaphragm for WT, ClC-1$^{\Delta E7a/\Delta E7a}$, Mbnl1$^{-/-}$, Mbnl1$^{-/-}$; ClC-1$^{\Delta E7a/\Delta E7a}$ samples (4-6-months old). Data presented a mean ± SEM with biological replicates shown as individual points. Comparisons with one-way ANOVA with post-hoc Tukey's multiple comparisons. (b) Histograms of minimum Feret's diameter for tibialis anterior fibers by fiber-type. Comparisons with two-way ANOVA (bin and genotype) with post-hoc multiple comparisons with stars representing intra-bin comparisons versus WT (red stars represent Mbnl1$^{-/-}$ versus WT). Multiplicity adjusted p-values shown as * p < 0.05, ** p < 0.01, *** p < 0.001, and p < 0.0001.

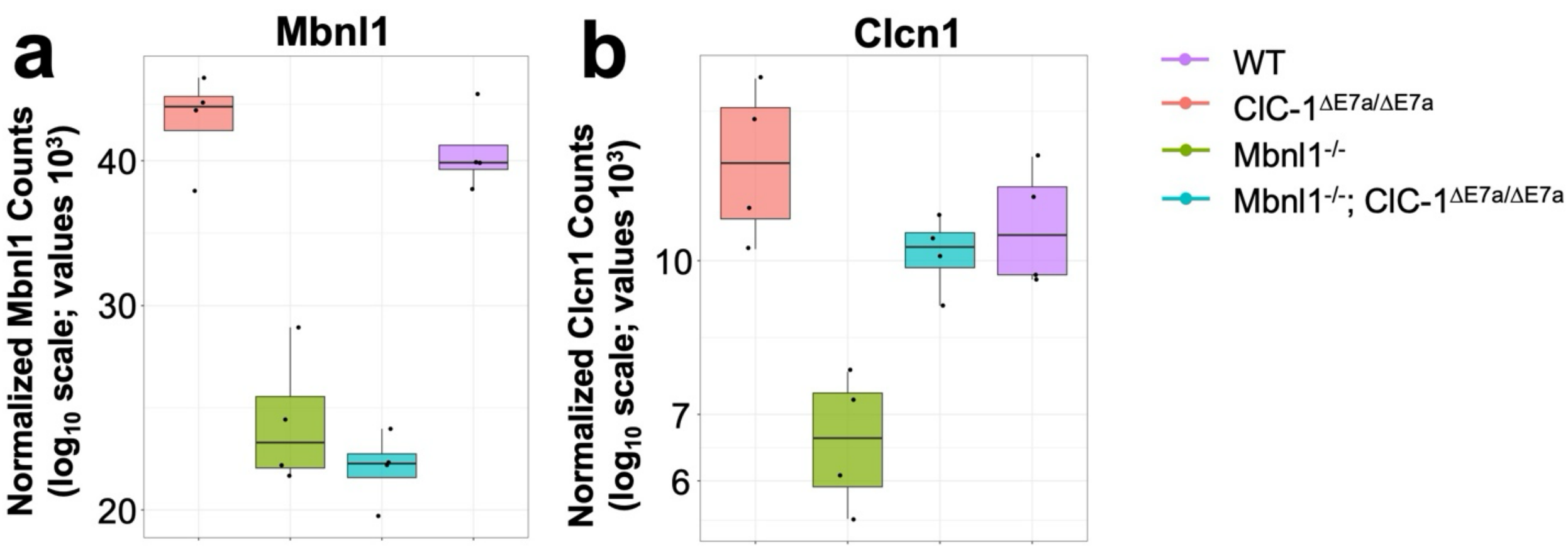

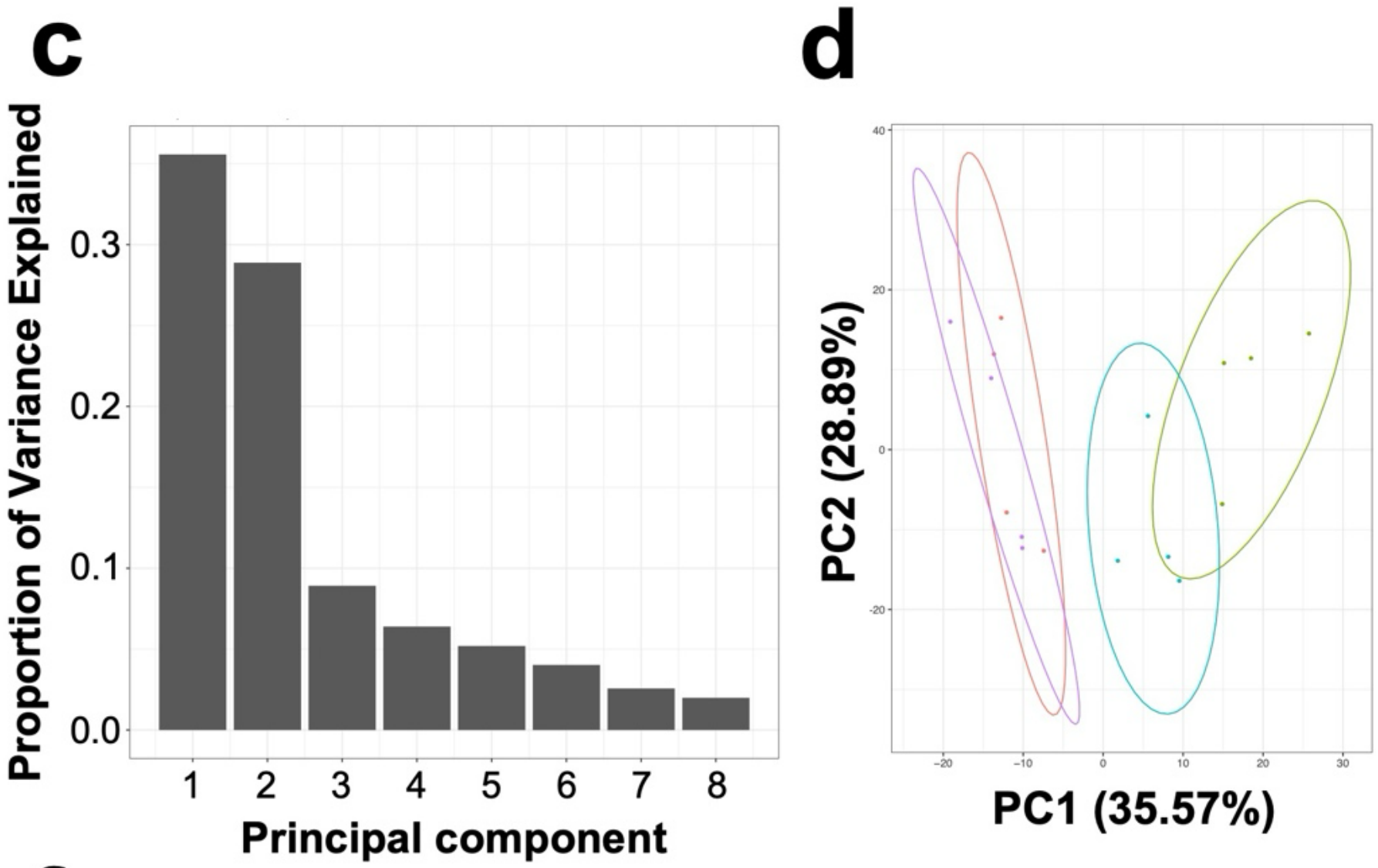

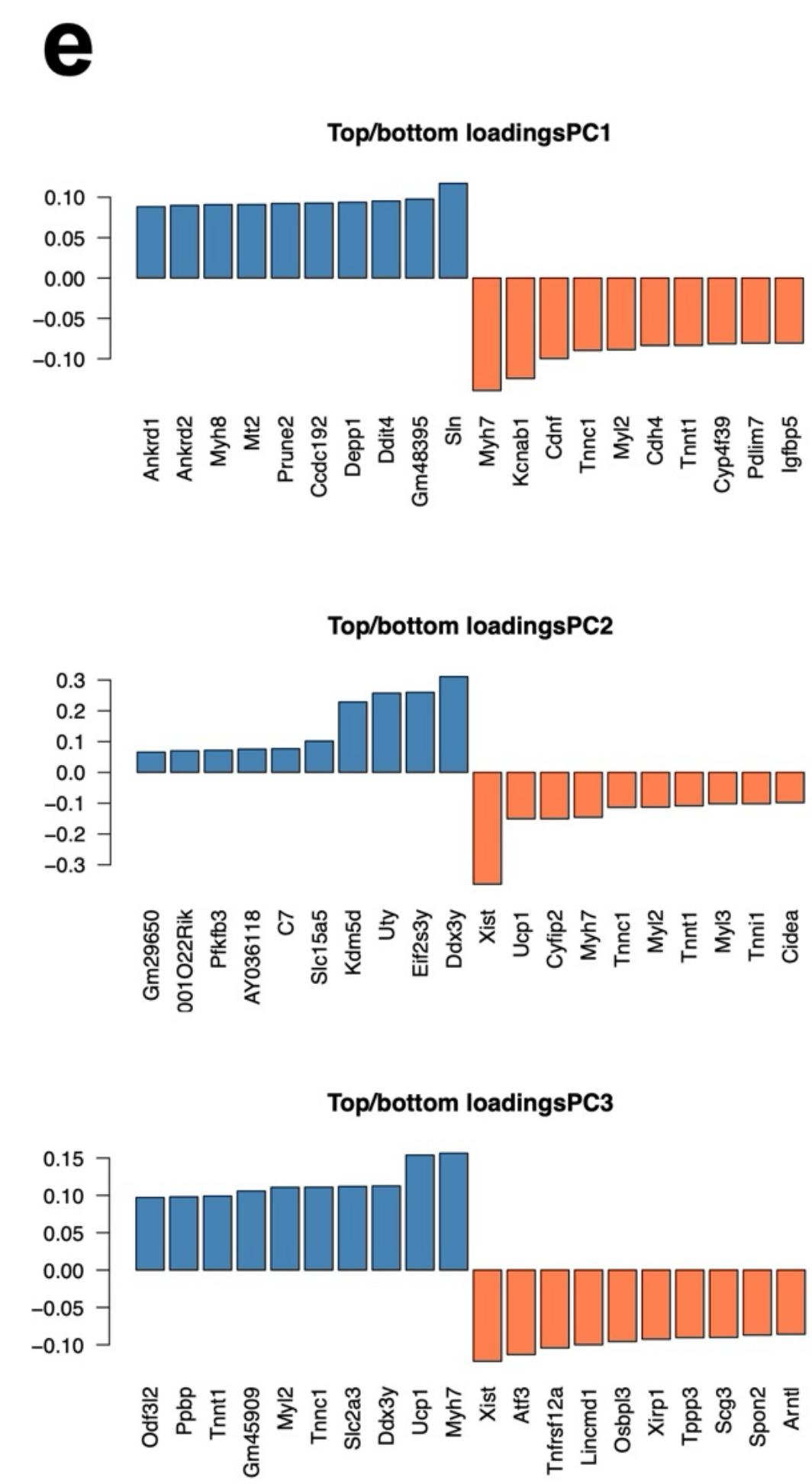

f

Term	P-value	Adjusted p-value	Odds ratio	Combined score	Genes
**Striated muscle contraction (GO:0006941)**	3.57E-09	3.14E-06	20.23514682	393.6007287	Csrp3;Myh3;Tnnt1;Myl2;Myl3; Myh8;Tnni1;Myh6;Myh7
**Heart contraction (GO:0060047)**	1.52E-07	6.68E-05	21.08533984	331.0642343	Csrp3;Actc1;Myl2;Myl3; Tnni1;Myh6;Myh7
**Myofibril assembly (GO:0030239)**	7.18E-07	1.45E-04	16.28497409	230.3788363	Csrp3;Myh3;Tnnt1;Myl2;Ankrd1; Myh6;Lmod2
**Skeletal muscle contraction (GO:0003009)**	9.87E-07	1.45E-04	36.23809524	501.1275974	Myh3;Tnnt1;Myh8;Tnni1;Myh7
**Cardiac muscle contraction (GO:0060048)**	9.88E-07	1.45E-04	21.83946981	301.988507	Csrp3;Myl2;Myl3;Tnni1; Myh6;Myh7
**Sarcomere organization (GO:0045214)**	9.88E-07	1.45E-04	21.83946981	301.988507	Csrp3;Myh3;Tnnt1;Ankrd1; Myh6;Lmod2

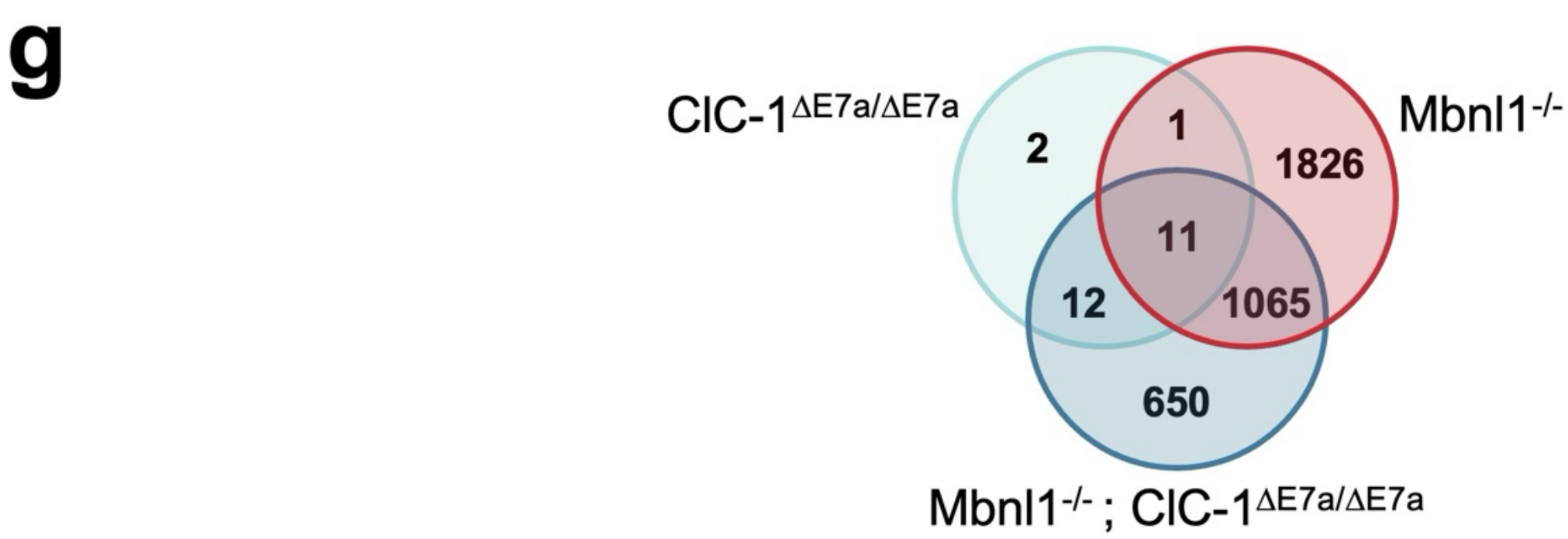

SF 8

Supplementary Figure 8 RNA-seq expression analysis. Normalized counts of reads for (a) *Mbnl1* and (b) *Clcn1* from RNAseq on approximately 4-6-month-old quadriceps tissue. Boxplots from pcaExplorer shown with individual values for each sample. (c) Scree plot of the variance accounted for by each principal component. (d) PCA plot with axes defined by PC1 and PC2. (e) Plot of top and bottom highest loading genes for PC1 through PC3. (f) Table displaying GO biological pathways analysis of top loading genes for PC1. (g) Venn diagram showing unique and shared dysregulated genes in ClC-1$^{\Delta E7a/\Delta E7a}$, Mbnl1$^{-/-}$, and Mbnl1$^{-/-}$; ClC-1$^{\Delta E7a/\Delta E7a}$ samples (compared to WT) with a threshold of absolute $\log_2$FC > 0.5 and adjusted p-value of < 0.05.

a

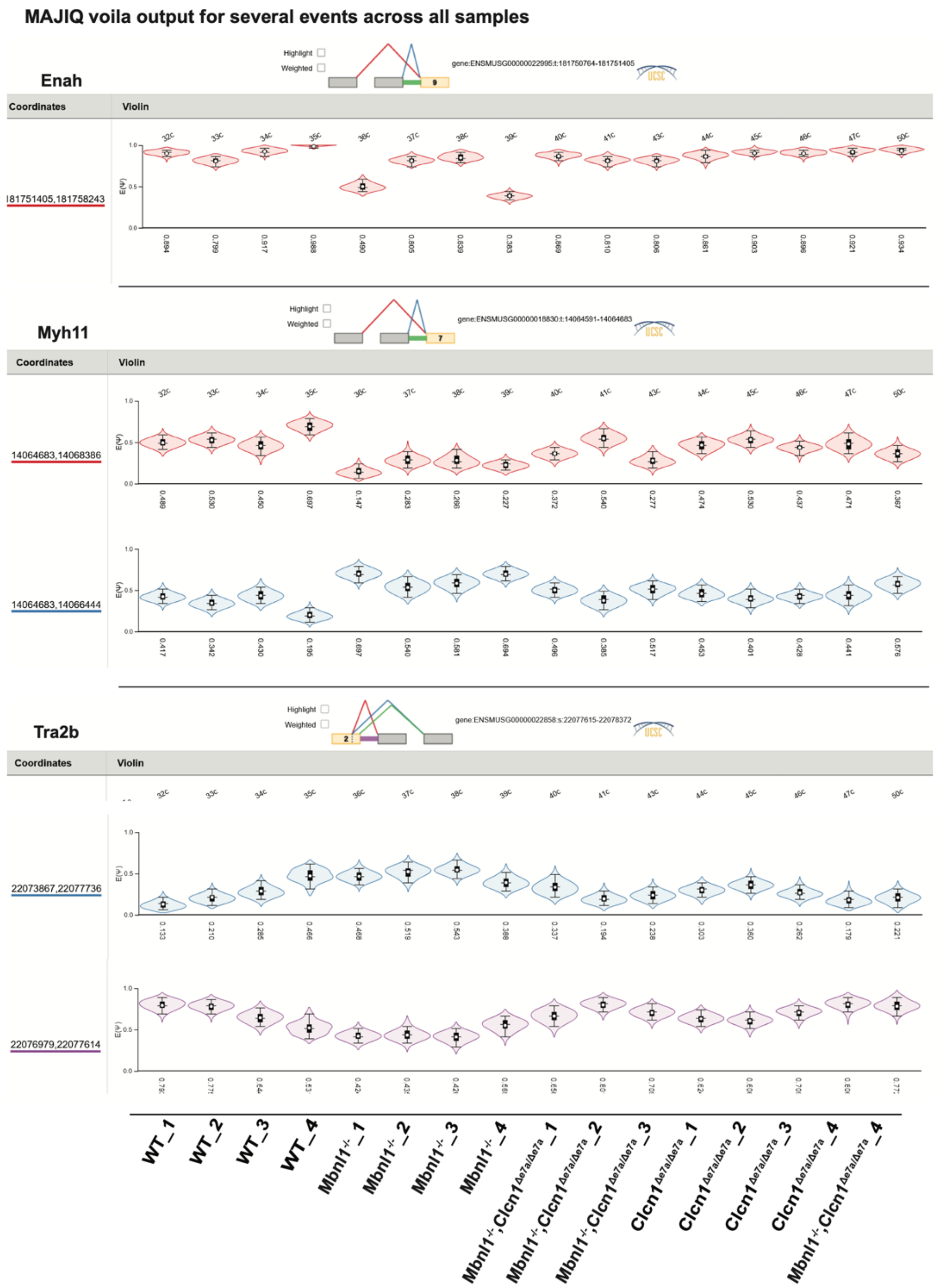

MAJIQ voila output for several events across all samples
Enah
Highlight
Weighted
gene:ENSMUSG00000022995:t:181750764-181751405
Coordinates
Violin
181751405,181758243
Myh11
gene:ENSMUSG00000018830:t:14064591-14064683
14064683,14068386
14064683,14066444
Tra2b
gene:ENSMUSG00000022858:s:22077615-22078372
22073867,22077736
22076979,22077614
WT_1
WT_2
WT_3
WT_4
Mbnl1-/-_1
Mbnl1-/-_2
Mbnl1-/-_3
Mbnl1-/-_4
Mbnl1-/-;Clcn1ΔE7a/ΔE7a_1
Mbnl1-/-;Clcn1ΔE7a/ΔE7a_2
Mbnl1-/-;Clcn1ΔE7a/ΔE7a_3
Clcn1ΔE7a/ΔE7a_1
Clcn1ΔE7a/ΔE7a_2
Clcn1ΔE7a/ΔE7a_3
Clcn1ΔE7a/ΔE7a_4
Mbnl1-/-;Clcn1ΔE7a/ΔE7a_4

b

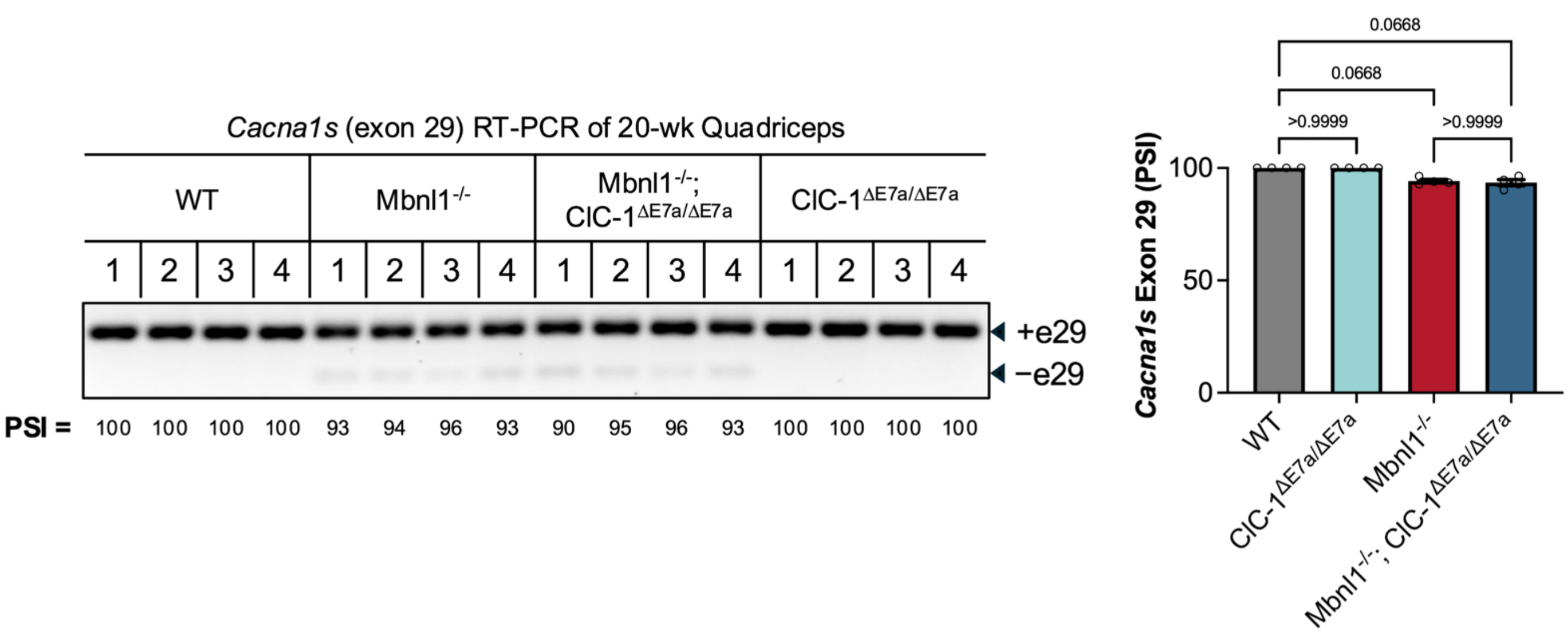

Cacna1s (exon 29) RT-PCR of 20-wk Quadriceps
WT
Mbnl1-/-
Mbnl1-/-; ClC-1ΔE7a/ΔE7a
ClC-1ΔE7a/ΔE7a
1 2 3 4 1 2 3 4 1 2 3 4 1 2 3 4
+e29
−e29
PSI = 100 100 100 100 93 94 96 93 90 95 96 93 100 100 100 100
0.0668
0.0668
>0.9999
>0.9999
Cacna1s Exon 29 (PSI)
100
50
0
WT
ClC-1ΔE7a/ΔE7a
Mbnl1-/-
Mbnl1-/-; ClC-1ΔE7a/ΔE7a

SF 9

Supplementary Figure 9 Splicing analysis. (a) Splicing analysis, MAJIQ output, for *Enah*, *Myh11*, and *Tra2b*. (b) Left: Agarose gel of RT-PCR products generated utilizing primers flanking exon 29 in *Cacna1s* from RNA isolated from quadriceps muscle of 4–6-month-old mice (n = 4 mice/genotype). Percent spliced-in (PSI) displayed below. Right: Quantification of $Ca_V1.1$ exon 29 PSI. Comparisons with one-way ANOVA with post-hoc comparisons (Tukey's) with adjusted p-value displayed.

a

Gene	Use	F/R	Sequence (5' → 3')
Clcn1	gDNA PCR (E7a)	F	CCTAGCCATATCATCCCCTCTGTCCA
		R	GCCCAGCAAATGAACAAATAGTGA
Clcn1[a]	RT-PCR (E2→E3)	F	GGGAATGCCCAAGAAGATGGGCT
		R	GCCATCAGGAGGCCCAGGAGCAC
Clcn1[b]	RT-PCR (E5→E8)	F	TGAAGGAATACCTCACACTCAAGG
		R	CACGGAACACAAAGGCACTG
Cacna1s[a]	RT-PCR (E28→E30)	F	GAGATCCTTGGAATGTGTTTGACTTCCT
		R	GGTTCAGCAGCTTGACCAGTCTCAT
Clcn1	PacBio (E1→E23)	F	5AmMC6/GCAGTCGAACATGTAGCTGACTCAGGTCACTTGTACCA GCTACGGACTGC
		R	5AmMC6/TGGATCACTTGTGCAAGCATCACATCGTAGCGTCCACTC TCTGACATGGG
Clcn1[c]	RT-qPCR (ss-mRNA)	F	TGGAGACTGTGTGGGAGAC
		Probe	5'6-FAM/CCAATTACT/ZEN/GCATAGCCCCCAGGT/3'IABkFQ
		R	CATTTGGAAGGCTGGTAGGAG
Clcn1	RT-qPCR (pre-mRNA)	F	CTGTGTCATTCCCATTGTACCC
		Probe	5'6-FAM/TGTCAAGAA/ZEN/AGACAGCTCCAACCCA/3'IABkFQ
		R	TGGAGACTGTGTGGGAGAC
Tbp[d]	RT-qPCR (reference)	F	CCAGAACTGAAAATCAACGCAG
		Probe	5'TET/ACTTGACCT/ZEN/AAAGACCATTGCACTTCGT/3IABkFQ
		R	TGTATCTACCGTGAATCTTGGC

(a) Yadava *et al.* (2020), (b) Wheeler *et al.* (2007), (c) Mm.PT.58.8747621 (IDT), (d) Mm.PT.39a.22214839 (IDT)

b

Primary Antibodies:

Anti-	Source	Product #	Isotype	Dilution
MyHC1	DSHB	BA-D5	MIgG2b	1:40
MyHC2a	DSHB	SC-71	MIgG1	1:40
MyHC2b	DSHB	BF-F3	MIgM	1:40
Laminin	Sigma-Aldrich	L9393	Rabbit poly	1:1000

Secondary Antibodies:

Anti-	Source	Product #	Fluorophore	Dilution
MIgG2b	Jackson Immuno.	115-477-187	DyLight 405	1:1000
MIgG1	Invitrogen	A-21124	Alexa 568	1:1500
MIgM	Invitrogen	A-21042	Alexa 488	1:1500
RIgG	Invitrogen	A-21245	Alexa 647	1:1000

ST 1

Supplementary Table 1 Oligonucleotides and antibodies. (a) Sequences of primers and probes used for genotyping PCR reactions, RT-PCR splicing analysis, full-length *Clcn1* transcript amplification, and RT-qPCR. Primers were obtained from previous studies as well as IDT designer, as indicated. (b) Primary and secondary antibodies used for MyHC IF.

Genotype:			Linear Properties:			Instantaneous Current Density IV:	Steady-State Current Density IV:	Relative Open Probability:		
	Age (days postnatal):	n	Capacitance (pF)	Rs (kOhms)	Tau (us)	Peak Instantaneous Current Density (pA/pF)	Peak Inward Current Density (pA/pF)	P_{min}	k	$V_{1/2}$ (mV)
WT	12	8	597±32	853±52	549±64	-40.9±1.7	-8.7±1.2	0.190±0.027	24.6±2.0	-74.7±2.3
ClC-$1^{\Delta E7a/\Delta E7a}$	12	11	494±25	1128±99	602±69	-50.6±4.9	-10.9±0.9	0.172±0.034	31.9±2.9	-66.5±3.0
Mbnl$1^{-/-}$	12	8	489±29	1059±51	560±40	-8.5±0.8	-1.3±0.1	0.179±0.018	19.9±1.6	-59.9±1.7
Mbnl$1^{-/-}$; ClC-$1^{\Delta E7a/\Delta E7a}$	12	8	502±19	1005±128	523±49	-54.5±4.4	-9.4±0.9	0.155±0.023	24.5±1.8	-66.2±2.0
WT	18	14	1082±39	697±40	816.5±45	-42.1±1.7	-9.5±0.7	0.192±0.022	31.8±2.1	-62.3±2.05
ClC-$1^{\Delta E7a/\Delta E7a}$	18	17	985±31	657±29	703±36	-63.5±4.2	-18.4±1.8	0.171±0.041	39.6±3.9	-64.9±3.74
Mbnl$1^{-/-}$	18	16	831±30	703±31	631±37	-25.3±1.2	-5.3±0.4	0.133±0.038	31.2±2.7	-75.2±3.19
Mbnl$1^{-/-}$; ClC-$1^{\Delta E7a/\Delta E7a}$	18	14	851±42	742±36	677±35	-56.4±2.7	-13.8±1.1	0.139±0.030	33.8±2.6	-72.2±2.58
ADR	18	7	1169±62	942±62	1129±81	0.031±0.06	n/a	n/a	n/a	n/a

ST 2

Supplementary Table 2 Whole-cell patch-clamp linear properties and fitting parameters. Table of linear properties and fitting properties for relative open probability for ClC-1 current recordings.
