# Supplementary material for: Elimination of myotonia improves myopathy in a muscleblind-like knockout model of myotonic dystrophy
Source: Nat Commun. 2026 Jul 8;17:8429. doi: 10.1038/s41467-026-75243-x (PMC13478325; doi:10.1038/s41467-026-75243-x)
Supplement: Supplementary file 2 [file 41467_2026_75243_MOESM2_ESM.pdf]

nature portfolio

Peer Review File

Elimination of myotonia improves myopathy in a muscleblind-like knockout model of myotonic dystrophy

Corresponding Author: Dr John Lueck

This file contains all reviewer reports in order by version, followed by all author rebuttals in order by version.

Version 0:

Reviewer comments:

Reviewer #1

(Remarks to the Author)
Summary
Amongst the inherited dystrophies of skeletal muscle, myotonic dystrophy (DM) is unique because of the combined symptoms of myotonia (stiffness caused by involuntary after-contractions with prolonged runs of action potentials) and dystrophic weakness. Both symptoms diminish quality of life, and because of the co-existence of these symptoms in specific muscle groups it has been hypothesized that myotonia contributes to the pathogenesis of progressive dystrophy. The importance of this question extends beyond understanding disease mechanism because a causal relationship would strongly influence the strategic approach for therapeutic intervention.

This study takes a new approach at dissecting the effects of myotonia and of the disruption of developmentally regulated RNA splicing that underlies the toxic gain-of-function in DM from expanded CUG repeats that sequester RNA-binding proteins. Myotonia in DM arises from a >50% loss of the resting fiber chloride conductance, as a result of RNA mis-splicing, particularly inappropriate retention of a cryptic exon (7a) that introduces a nonsense frameshift in Clcn1. The DM model herein is an extension of an RNA-binding protein deficient mouse (muscleblind-like null, mbnl1(-/-)) combined with a genomic deletion of exon (E7a) for the ClC-1 chloride channel. The data convincingly show:

1) Genomic deletion of Clcn1 E7a in the Mbnl1(-/-) background restored the correct RNA splicing for the Clcn1 transcript across multiple exons (e.g. intron 2, intron 6 in addition to exon 7a).

2) In the Mbnl1(-/-);Clcn1E7a(-/-) mouse, the ClC-1 conductance was fully restored to WT levels and myotonia was completely abolished.

3) In the absence of myotonia, muscle function improved in the DM Mbnl1(-/-) mouse, with increased specific force and partial correction of histological changes (increased central nuclei, fiber-type switching).

4) Transcriptomic comparison of WT, Mbnl1 ko without myotonia, and Mbnl1 ko with myotonia showed differential expression levels and splicing events. While half of all transcripts are mis-spliced in the Mbnl1 ko, a unique subset occurs in the presence of myotonia (cytoskeletal and chromatin organization, RNA splicing) that differs from Mbnl1 KO without myotonia (fewer total number, cytoskeletal organization).

Overall, this study supports the notion that myotonia has an important modulatory effect on the pathologic phenotype in DM and that specifically targeting E7a of CLCN1 has the potential for beneficial effects that extend beyond restoration of CLC-1 channels and suppression of myotonia.

Critique:

This study is well-designed, the data are presented clearly, and the author's conclusions are strongly supported by the data. The primarily limitation is explicitly stated at the end of the Discussion "Limitations of our study revolve around the use of a mouse model with mild to moderate DM1 phenotype". In fact, the most pronounced feature of the phenotype is myotonia with only mild evidence for myopathy or dystrophy. Why did the authors choose the Mbnl1(-/-) model for DM1 instead of the Mbnl1(-/-); Mbnl2(+/-) model with a more severe phenotype (Lee et al. EMBO Mol Med 2013, 5:1887)?

What is the confidence level for the improvement of peak specific force of Mbnl1(-/-) mice when myotonia is suppressed by deletion of Clcn1E7a (Fig. 4)? The concern is that the 28% reduction of peak specific force is entirely attributable to the calculated increase in cross sectional area (26% based on soleus weight, length, and pyknotic score). It appears as though the peak force (not normalized for CSA) would be comparable across all genotypes. Perhaps this could be shown in the Supplemental Data? Another concern is that DM should cause dystrophy with a reduction of CSA, not the increase of CSA observed for Mbnl1(-/-) mice (Fig 4f).

The inclusion of data from the ADR mouse (Clcn1 frameshift in exon 18) is an excellent control comparison, with lifelong severe myotonia in the absence of a primary splicing defect. Unexpectedly, ADR mice have multiple Clcn1 splicing defects (intron 2, exon 7a), presumably as a consequence of NMD (Figs. 1 and 2). A complete loss of the Cl conductance is shown (Fig 3a-d), but the corresponding effects on the late EMG (Fig 3g) and after-contraction (Fig. 3h) are not included. This would be an informative comparison to the Mbnl1(-/-) model of DM. More importantly, the ADR mouse would be an important control for the specific force measurements (Fig. 4) and the histological changes (Fig 5) should be non-dystrophic.

In the Introduction, the authors cite the DM1 splicing defect of CaV1.1, causing exon 29 skipping with a gain-of-function increase of Ca influx and late onset myopathy. With this being an established cause of myopathy, how would suppression of myotonia by genomic deletion of Clcn1 exon 7a be expected to attenuate myopathy? It might be worth examining the transcriptomic data to explicitly report on CaV1.1 exon 29 splicing for the non-myotonic Mbnl1(-/-); Clcn1E7a(-/-) mouse.

In the Results on page 11, the authors state "Interestingly, Mbnl1(-/-);ClC1E7a(+/-) mice with monoallelic deletion of E7a also lacked action myotonia". It is well known that reduction of the ClC-1 conductance to less than 50% of WT is required to cause myotonia. Perhaps the statement should therefore be revised to the effect: Consistent with the recessive and dominant-negative inheritance patterns of myotonia congenita from ClC-1 loss of function, MNbl1(-/-)…..

The utility of the rotarod test is a bit overstated (page 13) as a measure of "integrated effects of CLC-1 restoration on neuromuscular transmission, excitation-contraction coupling, muscle contraction and myotonia". First, the rotarod is primarily a test of coordination (cerebellar function). Second it is not clear what "failure to improve" over 4 trials (Fig 4h) means in terms of myopathy, contractility, or myotonia.

Reviewer #2

(Remarks to the Author)
The reviewer used an AI-based language tool to assist with English phrasing and clarity. The scientific content, interpretation, and opinions expressed are solely those of the reviewer.

Sipple et al. demonstrate that genomic deletion of a ClC-1 exon eliminates myotonia and ameliorates myopathy in a muscleblind knockout model of myotonic dystrophy. In addition to long-read sequencing analyses of CLCN1 mRNA splice patterns, the authors performed comprehensive transcriptome analyses including differential gene expression and alternative splicing. Functional improvements were assessed by measurements of muscle force, rotarod performance, and histopathological analyses. The experimental approach is methodologically sound, employs state-of-the-art techniques, and represents a thorough and carefully executed study. Importantly, the work carries potential clinical implications, as it provides initial evidence that long-term suppression of myotonia in myotonic dystrophy may mitigate aspects of progressive myopathy.

However, I would like to raise a concern regarding the interpretation of the reported improvement in muscle strength in the mouse model.

Muscle force was assessed using tetanic stimulation. It is well established that in myotonic muscle, repetitive stimulation can lead to a decrement in force generation. Therefore, the apparent improvement observed here may reflect amelioration of myotonia rather than a true enhancement of intrinsic contractile strength. Clarification of this distinction would strengthen the conclusions. Inclusion of comparative data from established myotonic mouse models (e.g., ADR mice), or additional measurements designed to assess intrinsic muscle force independent of myotonic phenomena, would help to more precisely interpret the functional outcomes.

Regarding the transcriptome analysis, PC2 is not presented in the main PCA figure but only in the supplemental material. The authors state that "PC2 was excluded as the top loading genes represented predominantly those localized to sex chromosomes." Further clarification is warranted. Were the mice matched for sex and age? If so, the presence of substantial sex chromosome–associated variance may represent a biologically relevant finding rather than technical noise. If not, this potential confounder should be explicitly addressed.

Finally, the authors have previously investigated Cacna1S in the context of DM1 pathophysiology. It would be important to clarify whether normalization of CLCN1 splicing in the present study also resulted in correction of splicing abnormalities in Cacna1S. Given the functional importance of CaV1.1 in skeletal muscle excitation–contraction coupling, this point may have mechanistic relevance.

Reviewer #3

(Remarks to the Author)

Sipple et al. investigate the role of myotonia in skeletal muscle pathology in myotonic dystrophy (DM1). Muscle weakness and wasting is the primary cause of early death in the disease. The molecular hallmark and cause of at least some manifestations of the disease is misregulated alternative splicing due to sequestration of the MBNL RNA binding protein on the expanded CUG repeat RNA transcribed from the mutant allele. A large number of genes undergo misregulated splicing and aberrant inclusion of exon 7a (E7a) of Clcn1 causes loss of the chloride channel in muscle resulting in the myotonia of myotonic dystrophy. This group previously showed that the combinatorial effects of loss of Clcn1 combined with misregulated splicing of the CACNA1S calcium channel caused severe muscle wasting in mice.

In this study the authors test the hypothesis that myotonia drives myopathy in DM1 and is a potential therapeutic target. They use Mbnl-/- mice that reproduce mild to moderate DM1-like myopathy. They performed rigourous analysis demonstrating inclusion of E7a resulted in loss of Clcn1 mRNA relative to the pre-mRNA and association of other alternative splicing events with loss of E7a. Genomic deletion of E7a produced full recovery of channel function and loss of myotonia in Mbnl1-/- mice. They show that Mbnl1-/- /ΔE7a mice improved force production and reversed transcriptome changes. Interestingly, loss of myotonia reversed misregulated splicing changes demonstrating that these are not directly regulated by loss of MBNL1.

The manuscript presents a rigorous analysis of physiological, histological, and molecular changes associated with a main feature of DM1 skeletal muscle. The results are a significant addition to the understanding of mechanisms of myopathy, specifically the previously underappreciated role of myotonia, and potential therapeutic target.

Comments address to minor concerns.
- Have the authors considered the impact of fiber type changes on the transcriptome analysis?
- Therapeutic possibilities were raised in the abstract and elsewhere but not addressed sufficiently as practical option.

Version 1:

Reviewer comments:

Reviewer #1

(Remarks to the Author)
In the revised manuscript, the authors have addressed each of the specific concerns raised by the 3 reviewers. The manuscript is improved. Importantly the revisions provide additional data (Supplemental Fig. 5 d, f, g) and more clearly state the limitations for functional assessment of myopathy in the Mbnl1-/- mouse model of DM1 (myopathic changes are mild in the mouse model but may be severe in human DM1; muscle CSA is increased in the mouse model, but decreased in human DM1 which makes the interpretation of specific force per CSA problematic). These revisions enable the reader to be more discerning in deciding whether the data showing improved muscle performance and histology in the setting of ClC-1 delE7a are derived from suppression of myotonia alone or from suppression of myotonia-aggravated DM myopathy as asserted by the authors.

Reviewer comments are provided in reply to some of the authors' responses to specific concerns:

1) The reviewer suggestion to use the Mbnl1-/-;Mbnl2+/- background as a more severe DM phenotype, and thereby have more prominent myopathy as readout, is impractical as nicely summarized in the response.

2) The suggestion to use the ADR mouse as a control for "myotonia only" in the specific force and CSA measurements was dismissed by the authors. Two reasons were given: (1) reduced specific force of the peak tetanic contraction was already reported for the ADR mouse in their prior publication (Cisco et al., 2024), and (2) "genetic correction of the ADR" would be required to use this line as a control. This reviewer disagrees. The prior publication did not provide data on the CSA histogram and so there is no basis to know the relative contribution of decreased force or increased CSA as a cause for the reduction in specific force. Moreover, the well-established fiber type switching (IIb -> IIA) in the setting of chronic myotonia is the major driver for reduced specific force (i.e. fiber type dependent) because in ADR muscle the fiber CSA is decreased (not increased as in the Mbnl1-/- mouse). Moreover, the impact of fiber type switching on overall macroscopic contractile properties will be muscle dependent (EDL is very different from soleus). And, with regard to the second point from the author, there is no need to correct the ADR defect by crossing with another mouse line.

3) With regard to interrogating the effect of suppressing myotonia on the DM splicing defect of CaV1.1 (exon 29 skipping), the authors added supplemental Fig. 9 to show that, unlike the case for human DM1, the Mbnl1-/- mouse has barely detectable drop out of exon 29. With this being the case, then why do the authors retain this statement in the Results on page 12 "Soleus was selected for these experiments because it exhibits high levels of mis-regulated splicing for CaV1.1 calcium channel in Mbnl1-/- mice, recreating the deleterious CaV1.1/ClC-1 bi-channelopathy that exists in human DM1 and have more severe myotonia than other hindlimb muscles in mouse models of MC"

Reviewer #2

(Remarks to the Author)
The revised manuscript has been substantially improved, and the authors have satisfactorily addressed the reviewer’s primary concerns.

Reviewer #3

(Remarks to the Author)
The authors have adequately addressed this reviewer's concerns

Open Access This Peer Review File is licensed under a Creative Commons Attribution 4.0 International License, which permits use, sharing, adaptation, distribution and reproduction in any medium or format, as long as you give appropriate credit to the original author(s) and the source, provide a link to the Creative Commons license, and indicate if changes were made.
In cases where reviewers are anonymous, credit should be given to 'Anonymous Referee' and the source.
The images or other third party material in this Peer Review File are included in the article’s Creative Commons license, unless indicated otherwise in a credit line to the material. If material is not included in the article’s Creative Commons license and your intended use is not permitted by statutory regulation or exceeds the permitted use, you will need to obtain permission directly from the copyright holder.
To view a copy of this license, visit https://creativecommons.org/licenses/by/4.0/

REVIEWER COMMENTS

We thank the editor and reviewers for taking the time to critically read and address concerns within the manuscript. We feel that this review process has improved the manuscript significantly. Each of the comments was addressed below in blue. Of note, we have included a designated reference cited page at the end of the "Response to Reviewers" for the reviewers to access the primary literature that support claims made.

Reviewer #1 (Remarks to the Author):

Summary
Amongst the inherited dystrophies of skeletal muscle, myotonic dystrophy (DM) is unique because of the combined symptoms of myotonia (stiffness caused by involuntary after-contractions with prolonged runs of action potentials) and dystrophic weakness. Both symptoms diminish quality of life, and because of the co-existence of these symptoms in specific muscle groups it has been hypothesized that myotonia contributes to the pathogenesis of progressive dystrophy. The importance of this question extends beyond understanding disease mechanism because a causal relationship would strongly influence the strategic approach for therapeutic intervention.

This study takes a new approach at dissecting the effects of myotonia and of the disruption of developmentally regulated RNA splicing that underlies the toxic gain-of-function in DM from expanded CUG repeats that sequester RNA-binding proteins. Myotonia in DM arises from a >50% loss of the resting fiber chloride conductance, as a result of RNA mis-splicing, particularly inappropriate retention of a cryptic exon (7a) that introduces a nonsense frameshift in Clcn1. The DM model herein is an extension of an RNA-binding protein deficient mouse (muscleblind-like null, mbnl1(-/-)) combined with a genomic deletion of exon (E7a) for the ClC-1 chloride channel. The data convincingly show:

1) Genomic deletion of Clcn1 E7a in the Mbnl1(-/-) background restored the correct RNA splicing for the Clcn1 transcript across multiple exons (e.g. intron 2, intron 6 in addition to exon 7a).

2) In the Mbnl1(-/-);Clcn1E7a(-/-) mouse, the ClC-1 conductance was fully restored to WT levels and myotonia was completely abolished.

3) In the absence of myotonia, muscle function improved in the DM Mbnl1(-/-) mouse, with increased specific force and partial correction of histological changes (increased central nuclei, fiber-type switching).

4) Transcriptomic comparison of WT, Mbnl1 ko without myotonia, and Mbnl1 ko with myotonia showed differential expression levels and splicing events. While half of all transcripts are mis-spliced in the Mbnl1 ko, a unique subset occurs in the presence of myotonia (cytoskeletal and chromatin organization, RNA splicing) that differs from Mbnl1 KO without myotonia (fewer total number, cytoskeletal organization).

Overall, this study supports the notion that myotonia has an important modulatory effect on the pathologic phenotype in DM and that specifically targeting E7a of CLCN1 has the potential for beneficial effects that extend beyond restoration of CLC-1 channels and suppression of myotonia.

Critique:

This study is well-designed, the data are presented clearly, and the author's conclusions are strongly supported by the data. The primarily limitation is explicitly stated at the end of the Discussion "Limitations of our study revolve around the use of a mouse model with mild to moderate DM1 phenotype". In fact, the most pronounced feature of the phenotype is myotonia with only mild evidence for myopathy or dystrophy. Why did the authors choose the Mbnl1(-/-) model for DM1 instead of the Mbnl1(-/-); Mbnl2(+/-) model with a more severe phenotype (Lee et al. EMBO Mol Med 2013, 5:1887)?

We thank the reviewer for the critique of the DM1 mouse model this study is based upon. To accomplish the main goal of this study, modeling the effects of global elimination of *Clcn1* exon 7a mis-splicing, we utilized CRISPR/Cas9 to generate a novel ClC-1$^{\Delta E7a}$ mouse model. Accordingly, to generate myotonic and non-myotonic DM1 mice, we utilized crossbreeding with an established DM1 line. When determining which DM1 model would be optimal for this study, we sought a model with clear and consistent transcriptomic and phenotypic manifestation of DM1 in skeletal muscle as well as a feasible breeding scheme for technical considerations. We thus primarily considered monogenetic models of DM1, namely the *HSA*^{LR} or Mbnl1$^{-/-}$ models.

Moreover, there were concerns that myotonia may directly alter expression of *HSA* and thus confound the results of the study. This was seen in Hu *et al.* (2023), which observed increased transcription of the *HSA (ACTA1)* transgene in *HSA*^{LR}; Mbnl1$^{-/-}$ mice treated with Clcn1 splice-shifting ASOs that eliminated myotonia in these mice. However, they observed no changes in endogenous *Dmpk* expression. Concomitant changes in transgene driven toxic-RNA burden and absence of myotonia could obfuscate transcriptomic changes in response to anti-myotonic interventions in these mice. Therefore, we chose to use Mbnl1$^{-/-}$ model with limitations as discussed in the limitations section included on Pg. 24.

Although, interrogating the effects of corrected Clcn1 splicing in the Mbnl1$^{-/-}$; Mbnl2$^{+/-}$ model would have provided additional information on the relevance of these findings in the setting of severe myopathy, these mice have an extremely severe phenotype limiting reproductive success. This requires breeding schemes that greatly limit the capability of obtaining mice of necessary genotypes. With the addition of another allele for selection (ClC-1$^{\Delta E7a}$), obtaining Mbnl1$^{-/-}$; Mbnl2$^{+/-}$; ClC-1$^{\Delta E7a/\Delta E7a}$ would have required ample resources with likely compensatory decreases in the breadth of experiments completed to account for this. In future studies, our goal is to translate these findings into more clinically applicable interventions that do not require crossbreeding to select three alleles.

What is the confidence level for the improvement of peak specific force of Mbnl1(-/-) mice when myotonia is suppressed by deletion of Clcn1E7a (Fig. 4)? The concern is that the 28% reduction of peak specific force is entirely attributable to the calculated increase in cross sectional area (26% based on soleus weight, length, and pyknotic score). It appears as though the peak force (not normalized for CSA) would be comparable across all genotypes. Perhaps this could be shown in the Supplemental Data? Another concern is that DM should cause dystrophy with a reduction of CSA, not the increase of CSA observed for Mbnl1(-/-) mice (Fig 4f).

We appreciate the observation from the reviewer that the in *ex vivo* force-contraction results demonstrate a deficit in specific force with concomitant increase in muscle cross-sectional area. This represents a decrease in force per unit area, likely secondary to shifts in fiber-type distributions (shifts to fiber-types with decreased maximal force output) or intrinsic myofiber dysfunction, but not a reduction in absolute force production. Accordingly, to address this comment we have added graphs showing absolute maximal force generation for both twitch and tetanic contraction to provided clarity on this point with associated text added to the manuscript. Additionally, we value your concern about the observation that DM1 is associated with muscle atrophy rather than hypertrophy with drops in specific force output seen here, indicating a discrepancy between mouse models of the disease and patient symptoms. Unfortunately, this is a caveat seen across many DM1 mouse models including both $Mbnl1^{-/-}$ and $Mbnl1^{-/-}$; $Mbnl2^{-/+}$, which both display increased muscle mass relative to their bodyweight (Davenport, Fong et al. 2025). However, although speculative, one could posit that if these fiber-type shifts hold true in the setting of atrophy, changes in both specific force and absolute force may result. To address this critique, we have added an additional statement to the limitations paragraph of the manuscript, included below.

Added data and text to address this critique are included below from Supplemental Fig. 5, main text pg. 13-14, and main text pg. 25-26 respectively.

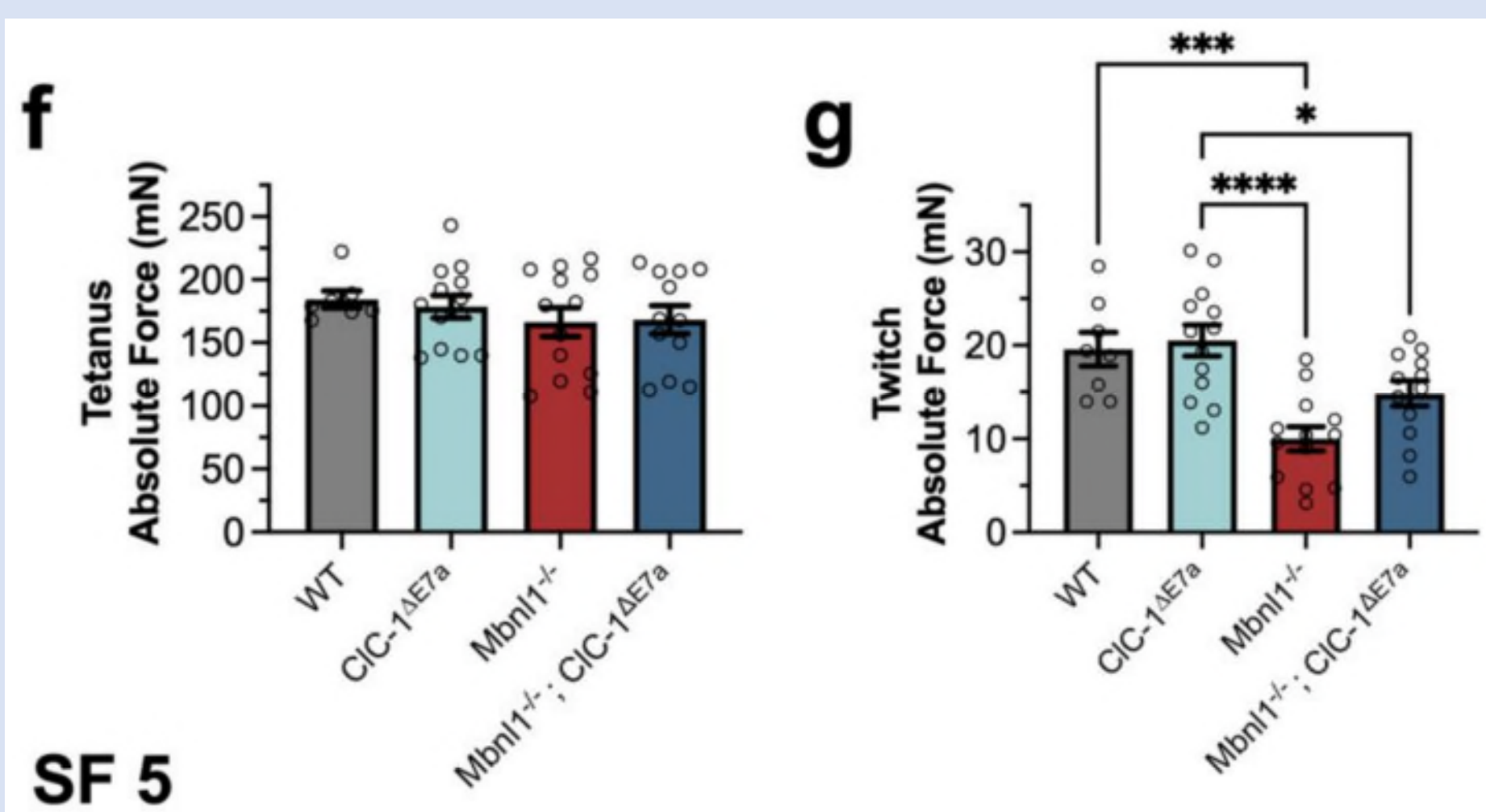

"Overall, the deficits in maximum specific force generation seen in Mbnl1$^{-/-}$ recordings were largely driven by a 26% increase in cross-sectional area (CSA) of Mbnl1$^{-/-}$ solei compared to WT (p = 0.029) with similar absolute force output in response to tetanic stimulation (Fig. S5f). Interestingly, peak absolute force generated in response to twitch stimulation was decreased in Mbnl1$^{-/-}$ muscles and significantly improved in Mbnl1$^{-/-}$; ClC-1$^{\Delta E7a}$ muscles (Fig. S5g). Furthermore, the change in CSA was not seen in Mbnl1$^{-/-}$; ClC-1$^{\Delta E7a}$ muscles, which were not significantly different from WT (p-adj. = 0.95; Fig. 4f). This indicates that the deficits seen are largely as a result of a decrease in contractile efficiency (force generation per unit area) consistent with altered intrinsic muscle function (e.g., excitation–contraction performance) and/or fiber-type remodeling.

"However, our findings also illuminate a limitation of extrapolating findings from murine models to DM1 patients, as many of the existing models regardless of apparent severity of phenotypic presentation feature findings, such as muscle hypertrophy, that do not fully recapitulate the skeletal muscle sequelae of DM1[35]. Despite these limitations, the numerous murine models of DM1 have served integral roles in interrogating disease mechanisms and preclinical studies investigating novel therapeutic approaches to DM1, and thus insights gained through these approaches warrant further clinical studies."

The inclusion of data from the ADR mouse (Clcn1 frameshift in exon 18) is an excellent control comparison, with lifelong severe myotonia in the absence of a primary splicing defect. Unexpectedly, ADR mice have multiple Clcn1 splicing defects (intron 2, exon 7a), presumably as a consequence of NMD (Figs. 1 and 2). A complete loss of the Cl conductance is shown (Fig 3a-d), but the corresponding effects on the late EMG (Fig 3g) and after-contraction (Fig. 3h) are not included. This would be an informative comparison to the Mbnl1(-/-) model of DM. More importantly, the ADR mouse would be an important control for the specific force measurements (Fig. 4) and the histological changes (Fig 5) should be non-dystrophic.

We appreciate this comment regarding further physiologic comparisons between our results and the ClC-1 null mouse model (ADR) that has been used as a model of non-dystrophic myotonia. In this study, we utilized comparisons with the ADR model primarily as a contrast between the introduction of a PTC in all transcripts by single nucleotide deletion versus alternative splicing driven shifts in reading frame resulting in a PTC (i.e., as an NMD control). This model also severed as a negative control for electrophysiologic recordings collected at the same timepoint, frequently collected simultaneously with tissue for RNA studies. We have previously published on the ADR model with regards to *in vitro* force contraction in aged mice as well as in the presence of verapamil, which functioned as a potent anti-myotonic agent in Cisco *et al.* (2024). These results showed significant myotonia as well as weakness with tetanic stimulation in ADR hindlimb muscles however, these mice did not show increased central nucleation on muscle histology. As genetic correction of the ADR is not possible with crosses with our novel ClC-1$^{\Delta E7a}$ model, and would be equivalent to a wildtype mouse, we did not pursue direct comparisons within this study. Furthermore, as ADR mice exhibit diminished lifespan, decreased muscle mass, and severe weakness

unlike patients with non-dystrophic myotonia, we felt that direct physiologic comparisons would not provide further impact of our presented findings.

To address this comment, comparisons with this prior work from our group (Cisco, Sipple et al. 2024) was added to the results on pages 14 and 15-16, included below...

“Furthermore, these findings align with our prior study $Ca_V1.1^{\Delta E29}$/ADR mice, which displayed significant specific force deficits in hindlimb muscles that were improved with the longitudinal treatment with verapamil, a drug that functioned as a potent anti-myotonic agent in these mice (Cisco, Sipple et al. 2024).”

“These finding also parallel those seen in hindlimb muscles of mice with isolated myotonia (ADR); however, those mice do not demonstrate any changes in central nucleation(Cisco, Sipple et al. 2024).”

In the Introduction, the authors cite the DM1 splicing defect of CaV1.1, causing exon 29 skipping with a gain-of-function increase of Ca influx and late onset myopathy. With this being an established cause of myopathy, how would suppression of myotonia by genomic deletion of Clcn1 exon 7a be expected to attenuate myopathy? It might be worth examining the transcriptomic data to explicitly report on CaV1.1 exon 29 splicing for the non-myotonic Mbnl1(-/-); Clcn1E7a(-/-) mouse.

We appreciate this suggestion to link the findings in this paper with our group’s prior published work establishing the role of altered CaV1.1 exon 29 splicing in modulating myopathy (Cisco *et al.* 2024). However, in the $Mbnl1^{-/-}$ line, there is minimal mis-splicing of CaV1.1 exon 29 at baseline (>90% percent spliced-in); therefore, this limited the ability to assay improvements in this splicing biomarker. As a single splicing event is unlikely to account for myopathy in DM1, these findings likely display the multifactorial nature of muscle weakness in DM1 that will be further discussed in the manuscript, as below. Furthermore, as part of our preliminary screen of RNA samples extracted from a, we completed RT-PCR experiments on RNA extracted from adult quadriceps muscle to assess CaV1.1 exon 29 splicing, shown below.

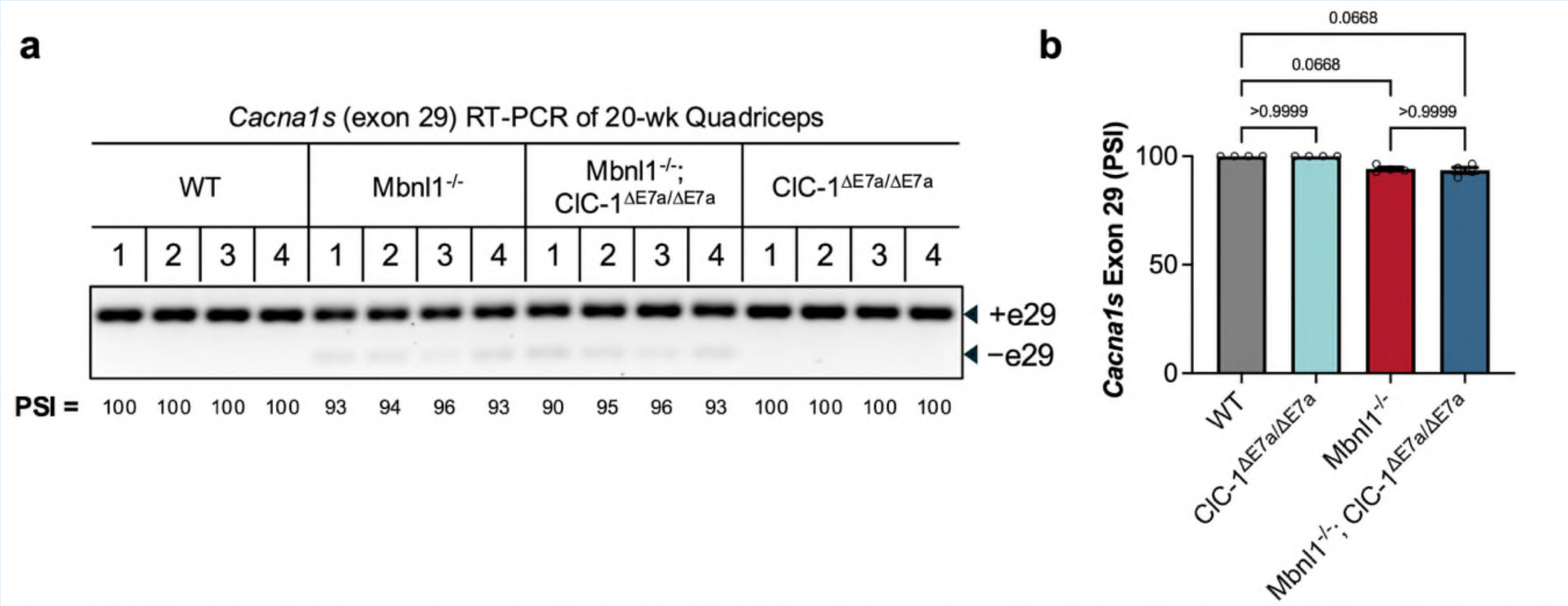

$Ca_V1.1$ splicing of exon 29 in myotonic and non-myotonic Mbnl1$^{-/-}$ mice. (a) Agarose gel of RT-PCR products generated utilizing primers flanking exon 29 in *Cacna1s* from RNA isolated from quadriceps muscle of 4–6-month-old mice (n = 4 mice/genotype). Percent spliced-in (PSI) displayed below. (b) Quantification of $Ca_V1.1$ exon 29 PSI. Comparisons with one-way ANOVA with post-hoc comparisons with p-value displayed.

Accordingly, to address this comment, this data was included in Supplemental Figure 9 and discussed in the text on page 19, included below.

"Despite our previous work demonstrating combinatorial effects of myotonia with $Ca_V1.1$ exon 29 exclusion, the Mbnl1$^{-/-}$ display minimal mis-splicing of *Cacna1s* (>90% PSI); thus, limiting our ability to probe the role of myotonia on this specific splice event (Supplemental Fig. 9b)."

In the Results on page 11, the authors state "Interestingly, Mbnl1(-/-);ClC1E7a(+/-) mice with monoallelic deletion of E7a also lacked action myotonia". It is well known that reduction of the ClC-1 conductance to less than 50% of WT is required to cause myotonia. Perhaps the statement should therefore be revised to the effect: Consistent with the recessive and dominant-negative inheritance patterns of myotonia congenita from ClC-1 loss of function, MNbl1(-/-)…..

We appreciate this critique, as based on prior literature in the field of myotonia congenita and animals with inherited chloride-channel myotonias, this finding is expected with prior estimates all exceeding 50% reduction in ClC-1 conductance to induce myotonia. Accordingly, to address this we have edited the portion of the manuscript quoted above and included the following statement with citation of appropriate literature (Pg. 11)

"Consistent with established literature in myotonia congenita and animals with genetic myotonia, greater than half of ClC-1 function must be lost to induce myotonia, the monoallelic deletion of E7a was sufficient to eliminate all evidence of myotonia in Mbnl1$^{-/-}$; ClC-1$^{\Delta E7a/+}$ mice. Subsequent physiologic experiments therefore used heterozygote and homozygote ClC-1$^{\Delta E7a}$ mice (identified as ClC-1$^{\Delta E7a}$ or Mbnl1$^{-/-}$; ClC-1$^{\Delta E7a}$) (Bryant and Morales-Aguilera 1971, Barchi 1975, Rüdel and Lehmann-Horn 1985, Colding-Jørgensen 2005)."

The utility of the rotarod test is a bit overstated (page 13) as a measure of "integrated effects of CLC-1 restoration on neuromuscular transmission, excitation-contraction coupling, muscle contraction and myotonia". First, the rotarod is primarily a test of coordination (cerebellar function). Second it is not clear what "failure to improve" over 4 trials (Fig 4h) means in terms of myopathy, contractility, or myotonia.

We appreciate this comment regarding the rotarod linear acceleration experiment (Fig. 4h) that demonstrated significant differences between myotonic and non-myotonic Mbnl1$^{-/-}$ mice. This assay was chosen based on its use by Lee *et al.* (2013) to characterize motor performance and mobility deficits in muscleblind-like knockout models. We observed that Mbnl1$^{-/-}$; ClC-1$^{\Delta E7a}$ mice performed equivalent to wildtype showing improvement in latency to fall with successive days of data collection. In contrast, myotonic Mbnl1$^{-/-}$ mice fail to improve, resulting in a significantly shorter latency to fall on the final day of testing; this indicates an overall deficit in mobility in the Mbnl1$^{-/-}$ mice that is completely resolved in Mbnl1$^{-/-}$; ClC-1$^{\Delta E7a}$. As this is a animal behavioral assay, encompassing everything from muscle to CNS functions, it is difficult to make direct claims on the specific origins of the deficits seen in the Mbnl1$^{-/-}$ mice. However, our claims largely stem from the direct comparison between Mbnl1$^{-/-}$ and Mbnl1$^{-/-}$; ClC-1$^{\Delta E7a}$ mice which differ only by resolution of the splicing of ClC-1, a chloride channel that has restricted expression to skeletal muscle. If deficits were derived primarily or partially from CNS dysfunction, we would have expected Mbnl1$^{-/-}$; ClC-1$^{\Delta E7a}$ mice to also display decreased performance and moreover not perform equivalent to wildtype.

To address this comment, we have edited the statement in question to provide more clear and accurate conclusions, as included below (Pg. 14-15).

"Overall *in vivo* muscle function was assessed by rotarod testing with a linear acceleration protocol. This approach is expected to integrate the effects of ClC-1 restoration on overall mobility, as previously utilized to assess Mbnl knockout models (Fig. 4g)(Lee, Li et al. 2013). Across the four days of trials, WT, ClC-1$^{\Delta E7a}$, and Mbnl1$^{-/-}$; ClC-1$^{\Delta E7a}$ mice significantly improved in their latency to fall, remaining on the rotarod for a significantly extended duration compared to myotonic Mbnl1$^{-/-}$ mice. The Mbnl1$^{-/-}$ mice failed to improve and showed a latency to fall that was significantly shorter than WT, ClC-1$^{\Delta E7a}$, and Mbnl1$^{-/-}$; ClC-1$^{\Delta E7a}$ mice (Fig. 4h), which highlights the functional impairment caused by myotonia in these animals. Notably, Mbnl1$^{-/-}$; ClC-1$^{\Delta E7a}$ mice performed equivalent to WT in this assay, indicating that deficits seen in Mbnl1$^{-/-}$ mice and the consequent improvement in Mbnl1$^{-/-}$; ClC-1$^{\Delta E7a}$ mice were due to changes in intrinsic muscle function by elimination of myotonia alone."

Reviewer #2 (Remarks to the Author):

The reviewer used an AI-based language tool to assist with English phrasing and clarity. The scientific content, interpretation, and opinions expressed are solely those of the reviewer.

Sipple et al. demonstrate that genomic deletion of a ClC-1 exon eliminates myotonia and ameliorates myopathy in a muscleblind knockout model of myotonic dystrophy. In addition to long-read sequencing analyses of CLCN1 mRNA splice patterns, the authors performed comprehensive transcriptome analyses including differential gene expression and alternative splicing. Functional improvements were assessed by measurements of muscle force, rotarod performance, and histopathological analyses. The experimental approach is methodologically sound, employs state-of-the-art techniques, and represents a thorough and carefully executed study. Importantly, the work carries potential clinical implications, as it provides initial evidence that long-term suppression of myotonia in myotonic dystrophy may mitigate aspects of progressive myopathy.

However, I would like to raise a concern regarding the interpretation of the reported improvement in muscle strength in the mouse model.

Muscle force was assessed using tetanic stimulation. It is well established that in myotonic muscle, repetitive stimulation can lead to a decrement in force generation. Therefore, the apparent improvement observed here may reflect amelioration of myotonia rather than a true enhancement of intrinsic contractile strength. Clarification of this distinction would strengthen the conclusions. Inclusion of comparative data from established myotonic mouse models (e.g., ADR mice), or additional measurements designed to assess intrinsic muscle force independent of myotonic phenomena, would help to more precisely interpret the functional outcomes.

We appreciate this critique that the specific force deficits seen in Mbnl1$^{-/-}$ and partial improved in Mbnl1$^{-/-}$; ClC-1$^{\Delta E7a}$ muscles in response to tetanic stimuli, could represent changes in force secondary to the presence or absence of myotonia, rather than true changes in intrinsic contractile efficiency. We completed force frequency protocols on all samples to obtain recordings of force generation from twitch to fused tetani. Across all stimulation frequencies, including isolated 0.2 ms twitch stimuli, we observed specific force deficits in Mbnl1$^{-/-}$ muscles and partial but significant recovery in force generation in Mbnl1$^{-/-}$; ClC-1$^{\Delta E7a}$ muscles, regardless of whether the stimulation protocol induced myotonia. Accordingly, we conclude that the changes in specific force generation we observed represent true changes in the efficiency of excitation contraction-coupling and a reduction in intrinsic muscle force and is not an artifact of myotonia.

To further clarify this finding, we have included representative traces across stimulation frequencies for the force-frequency results displayed in Fig. 4, included representative traces of twitch contractions and quantified peak twitch specific force output in Supplemental Fig. 5., and included further information on these findings in the main text on pg. 13 (included below).

Fig. 4

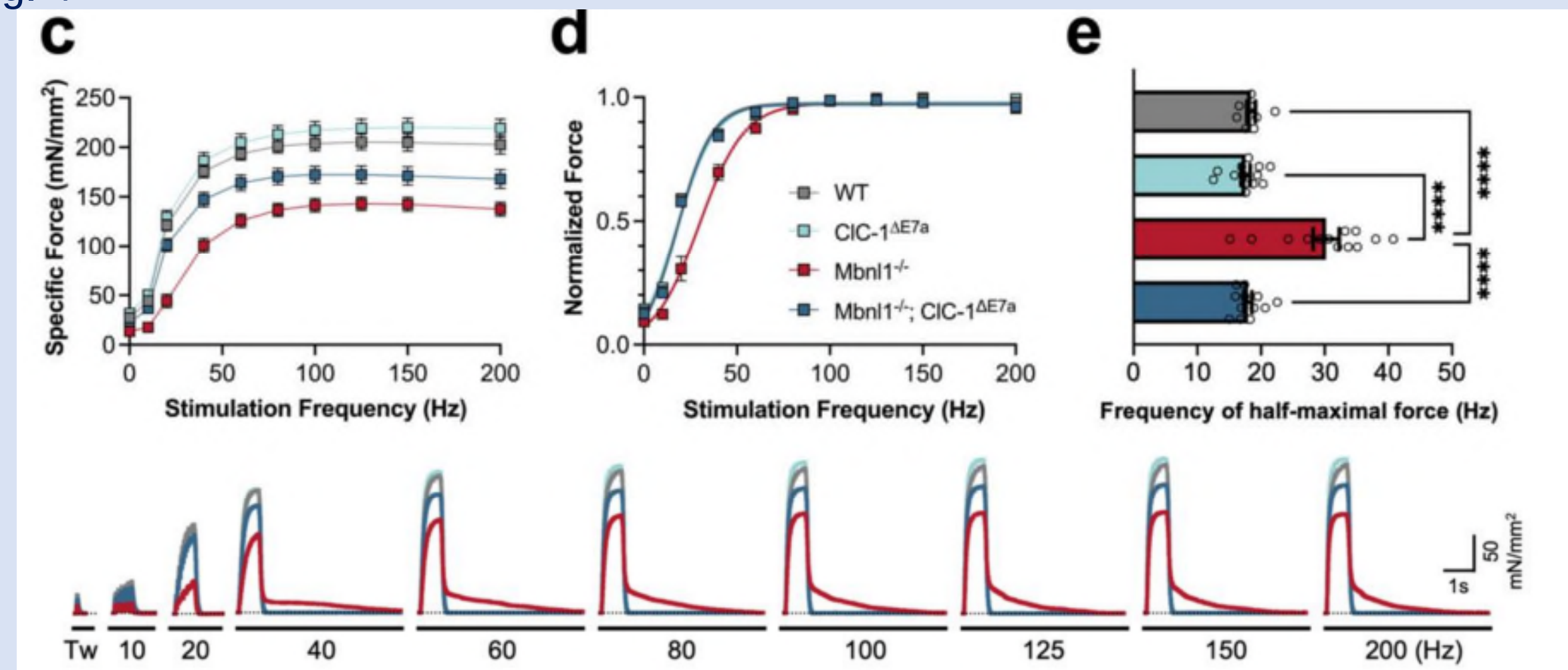

Supplemental Fig. 5

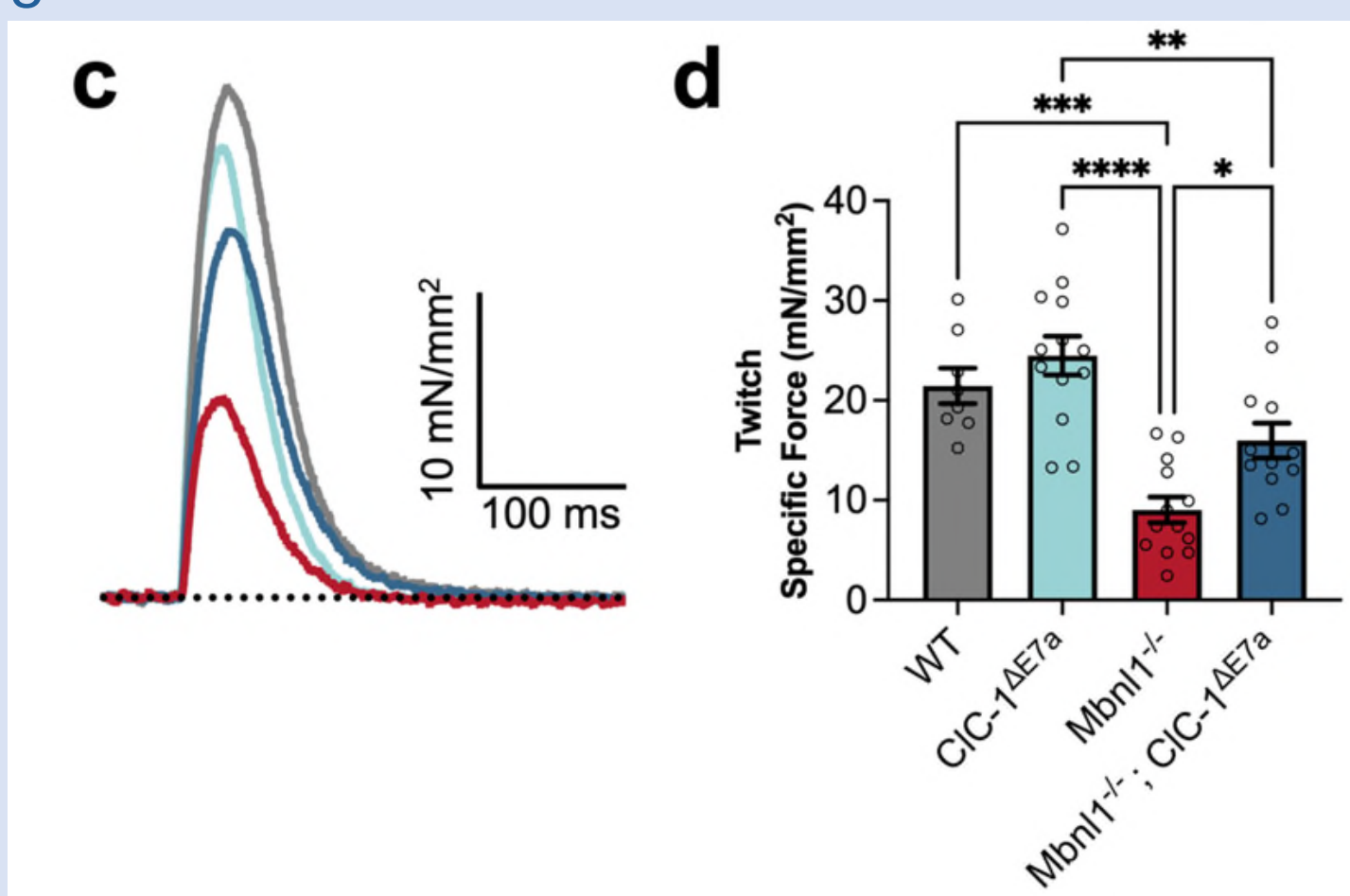

"Additionally, peak specific force for twitch recordings collected in response to a single 0.2 ms stimulus, displayed a similar reduction in specific force output for Mbnl1$^{-/-}$ muscles that was partially recovered Mbnl1$^{-/-}$; ClC-1$^{\Delta E7a}$ recordings (Fig. S5cd). This indicates that the partial recovery in specific force seen in Mbnl1$^{-/-}$; ClC-1$^{\Delta E7a}$ muscles is independent of the presence or absence of myotonia. Moreover, similar trends in specific force output were observed across stimulation frequencies, with a generalized reduction in Mbnl1$^{-/-}$ muscle compared to WT that was partially recovered in Mbnl1$^{-/-}$; ClC-1$^{\Delta E7a}$ muscle (Fig. 4c)."

Regarding the transcriptome analysis, PC2 is not presented in the main PCA figure but only in the supplemental material. The authors state that "PC2 was excluded as the top loading genes represented predominantly those localized to sex chromosomes." Further

clarification is warranted. Were the mice matched for sex and age? If so, the presence of substantial sex chromosome–associated variance may represent a biologically relevant finding rather than technical noise. If not, this potential confounder should be explicitly addressed.

We appreciate this comment regarding the utilization of PC1 and PC3 in the principal component analysis included in the main figure on the basis of PC2 featuring genes located on sex chromosomes as the predominant major loadings. For the completion of our RNAseq dataset we utilized mice of both sexes in all groups with even composition (2 male; 2 female) mice in the WT and ClC-$1^{\Delta E7a/\Delta E7a}$ samples, but unbalanced ratios in Mbnl$1^{-/-}$ (3:1) and Mbnl$1^{-/-}$; ClC-$1^{\Delta E7a}$ (1:3) samples limiting our ability to make direct claims on sexual-dimorphisms in the transcriptomic effects of myotonia in this study. Prior studies utilizing both sexes, have not demonstrated substantial transcriptomic differences across sexes in this DM1 model (Tanner, Tang et al. 2021); however, we understand this provides an additional confounding variable that limits the ability to assess any sex differences in our results. To address this, we clarified this point in the main text discussing these results and have provided additional information in the methods section.

Additional text in the results on pg. 17 and methods pg. 41 is included below,

"PC1 was plotted against PC3, as this provided the best clustering across our experimental groups, as sex distributions were unbalanced in Mbnl$1^{-/-}$ and Mbnl$1^{-/-}$; ClC-$1^{\Delta E7a/\Delta E7a}$ groups and PC2 was predominantly loaded with genes on sex chromosomes (Fig. 6a, Fig. S8c,d,e)."

"RNA was isolated from quadriceps muscle from 4–6-month-old WT (2:2), ClC-$1^{\Delta E7a/\Delta E7a}$ (2:2), Mbnl$1^{-/-}$ (3:1), and Mbnl$1^{-/-}$; ClC-$1^{\Delta E7a/\Delta E7a}$ (1:3, male to female) mice as described above (RNA isolation)."

Finally, the authors have previously investigated Cacna1S in the context of DM1 pathophysiology. It would be important to clarify whether normalization of CLCN1 splicing in the present study also resulted in correction of splicing abnormalities in Cacna1S. Given the functional importance of CaV1.1 in skeletal muscle excitation–contraction coupling, this point may have mechanistic relevance.

We appreciate this comment and have addressed this critique, as per the response to Reviewer #1's similar remark above.

For ease of review, the prior response is included below:

We appreciate this suggestion to link the findings in this paper with our group's prior published work establishing the role of altered CaV1.1 exon 29 splicing in modulating myopathy (Cisco *et al.* 2024). However, in the Mbnl$1^{-/-}$ line, there is minimal mis-splicing of CaV1.1 exon 29 at baseline (>90% percent spliced-in); therefore, this limited the ability to assay improvements in this splicing biomarker. As a single splicing event is unlikely to account for myopathy in DM1, these findings likely display the multifactorial

nature of muscle weakness in DM1 that will be further discussed in the manuscript, as below. Furthermore, as part of our preliminary screen of RNA samples extracted from a, we completed RT-PCR experiments on RNA extracted from adult quadriceps muscle to assess CaV1.1 exon 29 splicing, shown below.

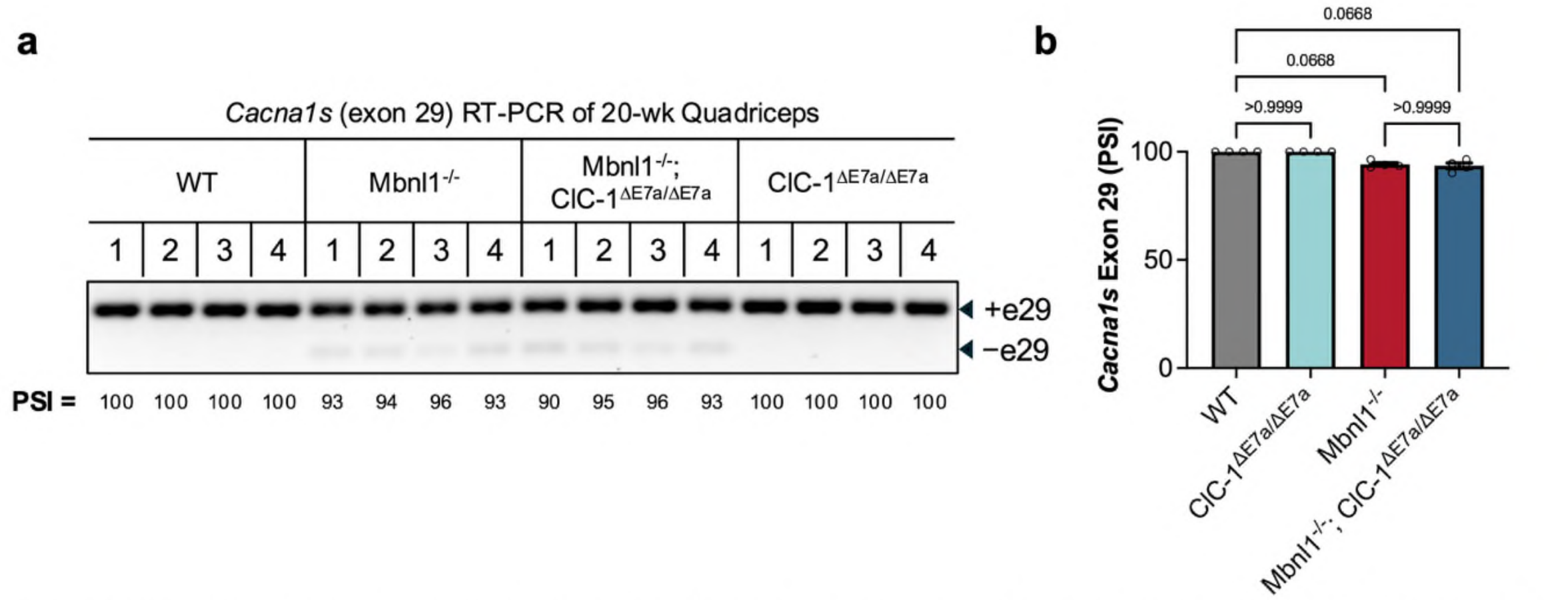

Ca$_V$1.1 splicing of exon 29 in myotonic and non-myotonic Mbnl1$^{-/-}$ mice. (a) Agarose gel of RT-PCR products generated utilizing primers flanking exon 29 in *Cacna1s* from RNA isolated from quadriceps muscle of 4–6-month-old mice (n = 4 mice/genotype). Percent spliced-in (PSI) displayed below. (b) Quantification of Ca$_V$1.1 exon 29 PSI. Comparisons with one-way ANOVA with post-hoc comparisons with p-value displayed.

Accordingly, to address this comment, this data was included in Supplemental Figure 9 and discussed in the text on page 19, included below.

"Despite our previous work demonstrating synthetic effects of myotonia with Ca$_V$1.1 exon 29 exclusion, Mbnl1$^{-/-}$ muscle displays minimal mis-splicing of *Cacna1s* (>90% PSI); thus, limiting our ability to probe the role of myotonia on this specific splice event (Supplemental Fig. 9). However, unsupervised clustering of these events further supports the concept that Mbnl1$^{-/-}$; ClC-1$^{\Delta E7a/\Delta E7a}$ mice represent an intermediate state between myotonic Mbnl1$^{-/-}$ and WT mice (Fig. 7c)."

Reviewer #3 (Remarks to the Author):

Sipple et al. investigate the role of myotonia in skeletal muscle pathology in myotonic dystrophy (DM1). Muscle weakness and wasting is the primary cause of early death in the disease. The molecular hallmark and cause of at least some manifestations of the disease is misregulated alternative splicing due to sequestration of the MBNL RNA binding protein on the expanded CUG repeat RNA transcribed from the mutant allele. A large number of genes undergo misregulated splicing and aberrant inclusion of exon 7a (E7a) of Clcn1 causes loss of the chloride channel in muscle resulting in the myotonia of myotonic dystrophy. This group previously showed that the combinatorial effects of loss of Clcn1 combined with misregulated splicing of the CACNA1S calcium channel caused severe muscle wasting in mice.

In this study the authors test the hypothesis that myotonia drives myopathy in DM1 and

is a potential therapeutic target. They use Mbnl-/- mice that reproduce mild to moderate DM1-like myopathy. They performed rigourous analysis demonstrating inclusion of E7a resulted in loss of Clcn1 mRNA relative to the pre-mRNA and association of other alternative splicing events with loss of E7a. Genomic deletion of E7a produced full recovery of channel function and loss of myotonia in Mbnl1-/- mice. They show that Mbnl1-/- /ΔE7a mice improved force production and reversed transcriptome changes. Interestingly, loss of myotonia reversed misregulated splicing changes demonstrating that these are not directly regulated by loss of MBNL1.

The manuscript presents a rigorous analysis of physiological, histological, and molecular changes associated with a main feature of DM1 skeletal muscle. The results are a significant addition to the understanding of mechanisms of myopathy, specifically the previously underappreciated role of myotonia, and potential therapeutic target.

Comments address to minor concerns.
- Have the authors considered the impact of fiber type changes on the transcriptome analysis?

We appreciate this comment, as alterations in fiber-type was seen both physiologically through changes in contractile properties and histologically through immunofluorescence of myosin heavy-chain isotypes. Indeed, this likely underlies a portion of the transcriptomic changes assessed through bulk RNAseq. Accordingly, we have commented on this in the manuscript quoted below (Pg. 16)…

"The top biological process in genes that were downregulated in Mbnl1$^{-/-}$ muscle compared to Mbnl1$^{-/-}$; ClC-1$^{\Delta E7a/\Delta E7a}$ was related to skeletal muscle contraction. The main driver of this alteration was reduced expression in myotonic muscle of genes that are markers for the type 1 fiber program, comprised mainly of myosin heavy or light chains and other components of the contractile apparatus (*Myh6, Myh7, Myh7b, Myl2, Tnnc1, Tnnt1, Tnni1, Tpm3*)(Petrany, Swoboda et al. 2020, Ham, Lin et al. 2025). Notably, Mbnl1$^{-/-}$ muscle showed concurrent upregulation of the 2x fiber marker, *Myh1*), which was also improved in Mbnl1$^{-/-}$; ClC-1$^{\Delta E7a/\Delta E7a}$ mice. Taken together with results of fiber typing above, these findings support the concept that myotonia drives calcium-mediated remodeling of muscle fibers and fit with the previous observation in ADR mice that reprogramming of muscle by myotonia is not simply a unidirectional shift from fast to slow fiber types but rather a convergence on the 2a/2x program from both ends of the fiber type spectrum (type 1 and type 2b)(Wu and Olson 2002)."

- Therapeutic possibilities were raised in the abstract and elsewhere but not addressed sufficiently as practical option.

We appreciate this comment regarding our speculation but limited practical consideration to possible therapeutics included in the original manuscript. Accordingly, to address this we have included/modified the following statements in the manuscript.

Discussion (pg. 24-25):

“Overall, the work presented here lays the foundation for establishing a pathologic role of myotonia in the development of myopathy in myotonic dystrophy (type 1 and 2) and provide initial indications that long-term removal of myotonia from myotonic dystrophy may prevent some of the progressive myopathy seen in the disease. Accordingly, further pre-clinical studies utilizing clinically available anti-myotonic agents or even clinical trials with longer-term follow-up are needed to fully ascertain whether these changes will hold true in settings more tangible to clinical practice. Several sodium-channel blockers, including mexiletine and ranolazine, have been utilized clinically across myotonic disorders, as they are generally well tolerated and provide significant improvements in myotonia(Arnold, Kline et al. 2017, Lorusso, Kline et al. 2019, Heatwole, Luebbe et al. 2021, Spillane, Trip et al. 2025). However, patients with DM1 were the least likely to have trialed anti-myotonic therapies across the genetic myotonias surveyed, with only 27% of DM1 patients surveyed having attempted treatment with any anti-myotonic agent (Meyer, Roggenbuck et al. 2020). Although, this study did not provide direct evidence to account for this discrepancy, it remains plausible that if a disease modifying role of these medications is established, further use of these medications could provide added benefits to DM1 patients.”

References

Arnold, W. D., D. Kline, A. Sanderson, A. A. Hawash, A. Bartlett, K. R. Novak, M. M. Rich and J. T. Kissel (2017). "Open-label trial of ranolazine for the treatment of myotonia congenita." Neurology **89**(7): 710-713.
Barchi, R. L. (1975). "Myotonia. An evaluation of the chloride hypothesis." Arch Neurol **32**(3): 175-180.
Bryant, S. H. and A. Morales-Aguilera (1971). "Chloride conductance in normal and myotonic muscle fibres and the action of monocarboxylic aromatic acids." J Physiol **219**(2): 367-383.
Cisco, L. A., M. T. Sipple, K. M. Edwards, C. A. Thornton and J. D. Lueck (2024). "Verapamil mitigates chloride and calcium bi-channelopathy in a myotonic dystrophy mouse model." The Journal of Clinical Investigation **134**(1).
Colding-Jørgensen, E. (2005). "Phenotypic variability in myotonia congenita." Muscle & Nerve **32**(1): 19-34.
Davenport, M. L., A. Fong, G. Montoya-Vazquez, M. F. A. de Moura, J. L. Bubenik and M. S. Swanson (2025). "Differential pathology and susceptibility to MBNL loss across muscles in myotonic dystrophy mouse models." JCI Insight **10**(18).
Ham, A. S., S. Lin, A. Tse, M. Thürkauf, T. J. McGowan, L. Jörin, F. Oliveri and M. A. Rüegg (2025). "Single-nuclei sequencing of skeletal muscle reveals subsynaptic-specific transcripts involved in neuromuscular junction maintenance." Nat Commun **16**(1): 2220.
Heatwole, C., E. Luebbe, S. Rosero, K. Eichinger, W. Martens, J. Hilbert, J. Dekdebrun, N. Dilek, C. Zizzi, N. Johnson, A. Puwanant, R. Tawil, G. Schifitto, C. A. Beck, J. F. Richeson, W. Zareba, C. Thornton, M. P. McDermott and R. Moxley, 3rd (2021). "Mexiletine in Myotonic Dystrophy Type 1: A Randomized, Double-Blind, Placebo-Controlled Trial." Neurology **96**(2): e228-e240.
Lee, K. Y., M. Li, M. Manchanda, R. Batra, K. Charizanis, A. Mohan, S. A. Warren, C. M. Chamberlain, D. Finn, H. Hong, H. Ashraf, H. Kasahara, L. P. Ranum and M. S. Swanson (2013). "Compound loss of muscleblind-like function in myotonic dystrophy." EMBO Mol Med **5**(12): 1887-1900.
Lorusso, S., D. Kline, A. Bartlett, M. Freimer, J. Agriesti, A. A. Hawash, M. M. Rich, J. T. Kissel and W. David Arnold (2019). "Open-label trial of ranolazine for the treatment of paramyotonia congenita." Muscle Nerve **59**(2): 240-243.
Meyer, A. P., J. Roggenbuck, S. LoRusso, J. Kissel, R. M. Smith, D. Kline and W. D. Arnold (2020). "Genotype-Phenotype Correlations and Characterization of Medication Use in Inherited Myotonic Disorders." Frontiers in Neurology **11**.
Petrany, M. J., C. O. Swoboda, C. Sun, K. Chetal, X. Chen, M. T. Weirauch, N. Salomonis and D. P. Millay (2020). "Single-nucleus RNA-seq identifies transcriptional heterogeneity in multinucleated skeletal myofibers." Nat Commun **11**(1): 6374.
Rüdel, R. and F. Lehmann-Horn (1985). "Membrane changes in cells from myotonia patients." Physiol Rev **65**(2): 310-356.
Spillane, J., J. Trip, G. Drost, C. G. Faber, M. G. Hanna, S. J. Nevitt and V. Vivekanandam (2025). "Drug treatment for myotonia." Cochrane Database Syst Rev **4**(4): Cd004762.

Tanner, M. K., Z. Tang and C. A. Thornton (2021). "Targeted splice sequencing reveals RNA toxicity and therapeutic response in myotonic dystrophy." Nucleic Acids Research **49**(4): 2240-2254.
Wu, H. and E. N. Olson (2002). "Activation of the MEF2 transcription factor in skeletal muscles from myotonic mice." J Clin Invest **109**(10): 1327-1333.

Response to REVIEWERS' COMMENTS

We greatly appreciate the thorough review of our manuscript. The suggestions provided have significantly improved the quality the study. Below are our point-by-point responses to the second round of reviewer comments in blue.

Reviewer #1 (Remarks to the Author):

In the revised manuscript, the authors have addressed each of the specific concerns raised by the 3 reviewers. The manuscript is improved. Importantly the revisions provide additional data (Supplemental Fig. 5 d, f, g) and more clearly state the limitations for functional assessment of myopathy in the Mbnl1-/- mouse model of DM1 (myopathic changes are mild in the mouse model but may be severe in human DM1; muscle CSA is increased in the mouse model, but decreased in human DM1 which makes the interpretation of specific force per CSA problematic). These revisions enable the reader to be more discerning in deciding whether the data showing improved muscle performance and histology in the setting of ClC-1 delE7a are derived from suppression of myotonia alone or from suppression of myotonia-aggravated DM myopathy as asserted by the authors.

Reviewer comments are provided in reply to some of the authors’ responses to specific concerns:

1) The reviewer suggestion to use the Mbnl1-/-;Mbnl2+/- background as a more severe DM phenotype, and thereby have more prominent myopathy as readout, is impractical as nicely summarized in the response.

We appreciate this comment and no further changes have been made to the manuscript regarding this point.

2) The suggestion to use the ADR mouse as a control for “myotonia only” in the specific force and CSA measurements was dismissed by the authors. Two reasons were given: (1) reduced specific force of the peak tetanic contraction was already reported for the ADR mouse in their prior publication (Cisco et al., 2024), and (2) “genetic correction of the ADR” would be required to use this line as a control. This reviewer disagrees. The prior publication did not provide data on the CSA histogram and so there is no basis to know the relative contribution of decreased force or increased CSA as a cause for the reduction in specific force. Moreover, the well-established fiber type switching (IIb -> IIA) in the setting of chronic myotonia is the major driver for reduced specific force (i.e. fiber type dependent) because in ADR muscle the fiber CSA is decreased (not increased as in the Mbnl1-/- mouse). Moreover, the impact of fiber type switching on overall macroscopic contractile properties will be muscle dependent (EDL is very different from soleus). And, with regard to the second point from the author, there is no need to correct the ADR defect by crossing with another mouse line.

We appreciate this response to our statement regarding the decision not to include ADR mice in our physiologic and histologic assays. While inclusion of ADR samples in this study would provide an additional comparison between murine models of DM1 and myotonia congenita (MC), these additional studies would not provide evidence on the role of myotonia in DM1 pathogenesis, which is the main goal of this study. We agree that these studies could provide

information on the role of fiber type switching observed in myotonic muscle, and in general on force production, as healthy mouse soleus is comprised of type 1 and 2a fibers (PMID: 9614104).

Prior studies have shown that ADR mice, despite displaying robust myotonia, do not have increased central nucleation and exhibit reduced body size and muscle mass in contrast to patients with MC (PMID: 21356096). Further, our prior work we have observed weakness in these mice discordant with patients with MC (Cisco *et al*., 2024). Although severe muscle weakness was directly quantified in the EDL and diaphragm, severe weakness was likely occurring in all skeletal muscle, albeit to varying severities, as indicated by the reduced time of right reflexes which relies on trunk and other limb muscle groups. Therefore, when completing the present study, we did not feel that allocating additional ADR mice (and controls) for the purpose of assessing characteristics in soleus muscle was warranted. In the end, we believe our study provides a comprehensive physiologic assessment of the impact of myotonia on muscle function in DM1 mouse models.

3) With regard to interrogating the effect of suppressing myotonia on the DM splicing defect of CaV1.1 (exon 29 skipping), the authors added supplemental Fig. 9 to show that, unlike the case for human DM1, the Mbnl1-/- mouse has barely detectable drop out of exon 29. With this being the case, then why do the authors retain this statement in the Results on page 12 “Soleus was selected for these experiments because it exhibits high levels of mis-regulated splicing for CaV1.1 calcium channel in Mbnl1-/- mice, recreating the deleterious CaV1.1/ClC-1 bi-channelopathy that exists in human DM1 and have more severe myotonia than other hindlimb muscles in mouse models of MC”

We appreciate this response to our statement regarding the use of the soleus in this manuscript. We have modified this statement to align specifically with the findings presented in the manuscript. The new statement is included below for ease of review… (Pg. 12)

“Soleus was selected for these experiments, as it has been shown to exhibit more severe myotonia than other hindlimb muscles in the absence of ClC-1[36,37]”

Reviewer #2 (Remarks to the Author):

The revised manuscript has been substantially improved, and the authors have satisfactorily addressed the reviewer’s primary concerns.

We appreciate the reviewer’s comment and thorough review of our manuscript. No changes have been made regarding this point.

Reviewer #3 (Remarks to the Author):

The authors have adequately addressed this reviewer's concerns

We appreciate the reviewer’s comment and thorough review of our manuscript. No changes have been made regarding this point.
